# The MAGA movement and political violence in 2024: findings from a nationally representative survey

**DOI:** 10.1186/s40621-025-00633-6

**Published:** 2025-11-19

**Authors:** Garen J. Wintemute, Bradley Velasquez, Sonia L. Robinson, Elizabeth A. Tomsich, Mona A. Wright, Aaron B. Shev

**Affiliations:** 1 Department of Emergency Medicine, School of Medicine, UC Davis, Sacramento, CA USA; 2 Centers for Violence Prevention, University of California Davis, Sacramento, CA USA; 3 Department of Emergency Medicine, Centers for Violence Prevention, UC Davis, 2315 Stockton Blvd., Sacramento, CA 95814 USA

**Keywords:** Political violence, MAGA republican, MAGA movement, Domestic violent extremism, Democracy, Authoritarianism, Political parties, Political ideology, Firearms, Firearm violence, Violence and society, Racism, Hostile sexism, Homonegativity, Homophobia, Transphobia, Xenophobia, Antisemitism, Islamophobia, QAnon, Christian nationalism, Trait aggression, Conspiracism, Intimate partner violence

## Abstract

**Background:**

Too little is known about the distribution of risk for committing political violence, a serious concern for the United States. This study explores the association between affiliation with the “Make America Great Again” (MAGA) movement and support for and willingness to engage in political violence.

**Methods:**

Findings are from Wave 3 of a nationally representative annual longitudinal survey of members of the Ipsos KnowledgePanel, conducted May 23-June 14, 2024. All KnowledgePanel members who responded to prior waves were invited to participate. Political party and MAGA affiliations were reported by respondents; the principal comparison is between MAGA Republicans and non-MAGA non-Republicans. Outcomes are expressed as weighted proportions and adjusted prevalence differences (aPDs, reported as percentage point (pp) differences), with p-values adjusted for the false discovery rate.

**Results:**

The completion rate was 88.4%; there were 8896 respondents. MAGA Republicans were more likely than non-MAGA non-Republicans to endorse violence to effect sociopolitical change and to consider violence usually or always justified to advance at least 1 of 21 specific political objectives (MAGA Republicans, 55.9% (95% CI 52.3%, 59.4%); non-MAGA non-Republicans, 25.5% (95% CI 23.7%, 27.2%); aPD 30.1pp (95% CI 26.0pp, 34.2pp), q < 0.001). They were not more willing to commit political violence. Similarly, while MAGA Republicans more frequently predicted that they would be armed in a setting where they considered political violence justified, they were not more likely to shoot someone (very or extremely likely: MAGA Republicans, 2.1% (95% CI 0.8%, 3.4%); non-MAGA non-Republicans, 1.6% (95% CI 1.0%, 2.1%); aPD 1.5pp (95% CI -0.1pp, 3.0pp), q = 0.43). Prevalences for other Republicans generally fell between those for MAGA Republicans and non-MAGA non-Republicans. In secondary analyses, MAGA Republicans endorsed attributes associated with political violence—racism, hostile sexism, homonegativity, transphobia, xenophobia, and Islamophobia; support for the QAnon movement and Christian nationalism; conspiracism; trait aggression; and authoritarianism—more frequently than did non-MAGA non-Republicans.

**Conclusions:**

In 2024, MAGA Republicans were more likely than others to endorse political violence and attributes associated with political violence. They were not more willing to commit political violence themselves; their endorsement may increase the risk that political violence will occur.

**Supplementary Information:**

The online version contains supplementary material available at 10.1186/s40621-025-00633-6.

## Background

For several years, researchers and other experts in the field have expressed concern that political violence might erupt in the United States (US) [1–15]. In mid-2022, we initiated the annual, nationally representative, longitudinal, Life in America Survey on support for and willingness to commit political violence [16]. The research proceeds from the knowledge that violence is a health problem and that evidence-based prevention is possible. The overarching aim of the research has been to identify characteristics of respondents that are associated with support for and willingness to commit political violence, so as to facilitate prevention efforts.

Several studies [2, 17–19], but not all [20, 21], have found greater support for political violence among Republicans than among Democrats. Data from 2022’s Wave 1 of our survey revealed that “Make America Great Again” (MAGA) Republicans—defined by us as Republicans who voted for Donald Trump in 2020 and agreed strongly or very strongly with the statement that “the 2020 election was stolen from Donald Trump, and Joe Biden is an illegitimate president”—were more likely than others to support political violence [22]. For example, 58.2% (95% confidence interval (CI) 55.0%, 61.4%) of MAGA Republicans and 25.1% (95% CI 23.6%, 26.7%) of non-Republicans considered violence justified to advance at least 1 of 17 specified political objectives. MAGA Republicans were not more willing than others to commit political violence themselves, however [22]. Three other surveys [23–25] and a qualitative investigation [26] have also found links between MAGAism and support for political violence.

MAGAism has become one of the largest and most influential sociopolitical movements in the US [26]—and, due to its influence here, one of the most important in the world. In our 2022 survey [22], 33.6% of Republicans (15.0% of respondents) were classified as MAGA Republicans. In public opinion polls conducted since then, at least 40% of Republicans have identified themselves as MAGA Republicans or endorsed the MAGA movement [27–30]. MAGAism’s size and influence, and the relationship between MAGA affiliation and political violence, were of particular interest in 2024—a federal election year in the US.

Existing data on the association between MAGA affiliation and political violence are insufficient. In our earlier study [22], MAGA affiliation was determined by the investigators. Of the 3 other surveys, 2 reported only very limited findings [23, 25] and the other relied on a small, non-probability sample [24].

We designed 2024’s Wave 3 of the survey in part to revisit and expand our knowledge of the relationship between MAGA affiliation and political violence. In Wave 3, MAGA affiliation—as a “MAGA Republican” or “supporter of the MAGA movement”—was reported by respondents themselves. Our primary objective for this analysis was to describe associations between MAGA affiliation and a wide array of measures of support for and willingness to commit political violence. We also explored associations between MAGA affiliation and other respondent characteristics that our survey or research by others has found to be linked to political violence. Beyond sociodemographics [16], these characteristics include political party affiliation and ideology [17]; racism and other forms of fear, hatred, and enmity toward others [31]; support for extremist organizations and social movements [312]; firearm ownership and recent firearm purchase [33]; trait aggression [34]; and conspiracism [35]. The study’s aim is to contribute to a body of evidence that can underlie effective political violence prevention efforts.

## Methods

Methods for Wave 3 of this longitudinal survey closely followed those for Waves 1 and 2 [16, 36]. Wave 3 was designed by the authors. Sampling and data collection were conducted by the survey research firm Ipsos [37], who administered the survey online in English and Spanish from May 23 to June 14, 2024. The study was reviewed by the University of California Davis Institutional Review Board (protocol 187125: exempt from full review, category 2, survey research). The IRB waived a requirement for written or verbal consent. Before participants accessed the questionnaire, they were provided informed consent language that concluded, “[by] continuing, you are agreeing to participate in this study.” The study is reported following American Association for Public Opinion Research guidelines [38].

### Participants

Participants for Wave 1 were drawn from the Ipsos KnowledgePanel, an online research panel that has been widely used in population-based research on violence and firearm ownership [39–44]. To establish a nationally representative panel, KnowledgePanel members are recruited on an ongoing basis through address-based probability sampling using data from the US Postal Service’s Delivery Sequence File [45, 46]. Recruitment into KnowledgePanel involves repeated contact attempts, if necessary, by mail and telephone. Recruited adults in households without internet access are provided a web-enabled device and free internet service, and a modest, primarily points-based incentive program seeks to encourage participation and promote participants’ retention in KnowledgePanel over time [45, 46].

A probability-proportional-to-size procedure was used to select a study-specific sample for Wave 1. All panel members who were aged 18 years and older were eligible for selection. Invitations were sent by e-mail; automatic reminders were delivered to non-respondents by e-mail and telephone beginning 3 days later [45, 46].

The Wave 1 survey was conducted May 13 to June 2, 2022. It included a main sample, which had a completion rate of 53% and provided the study population for our initial report [16], and oversamples of firearm owners, transgender people, combat veterans, and California residents that were recruited to ensure adequate statistical power for planned analyses. Compared with main sample nonrespondents, main sample respondents were older and more frequently white, non-Hispanic; were more often married; had higher education and income; and were less likely to be working [16].

The survey cohort’s participation history across Waves 1–3 is presented in Figure S1. Including the main sample and oversamples, Wave 1 comprised 12,947 respondents. Of those respondents, 11,140 (86.0%) remained active members of KnowledgePanel on Wave 2’s launch date and were invited to participate in Wave 2. (The remaining 1807 Wave 1 respondents had left the cohort through normal attrition.)

Wave 2 had 9385 respondents (completion rate of 84%), of whom 8932 (95.2%) remained active members of KnowledgePanel on Wave 3’s launch date and were invited to participate in Wave 3. (Another 453 Wave 2 respondents had left the cohort through normal attrition.) Invitations to participate in Wave 3 were also sent to 1132 Wave 1 respondents who had not participated in Wave 2 and remained active members of KnowledgePanel on Wave 3’s launch date. (Another 716 Wave 1 respondents who did not respond to Wave 2 had left the cohort through normal attrition.)

A final Wave 3 survey weight variable provided by Ipsos adjusted for the initial probability of selection into KnowledgePanel and for survey-specific nonresponse and over- or under-coverage using design weights with post-stratification raking ratio adjustments. As with prior samples, the weighted 2024 sample is designed to be statistically representative of the noninstitutionalized adult population of the US as reflected in the 2021 March supplement of the Current Population Survey [45, 46].

### Measures

Sociodemographic data were collected by Ipsos from profiles created and maintained by KnowledgePanel members. Our exposure of primary interest concerned respondents’ self-reported status as MAGA Republicans or as supporters of the MAGA movement. These terms were not defined in the questionnaire. Participants were asked, “Generally speaking, do you think of yourself as…” with response options “Republican,” “Democrat,” “independent,” and “something else.” Those who chose Republican or Democrat were asked, “Would you call yourself a…” with response options “strong Republican” and “not very strong Republican” or “strong Democrat” and “not very strong Democrat.” Those who chose “independent” or “something else” for the first question were asked, “Do you think of yourself as closer to the…” with response options “Republican Party,” “Democratic Party,” and “do not lean either way.” Respondents who identified as Republican or closer to the Republican Party were asked, “Do you think of yourself as a MAGA Republican?” with response options “yes” and “no.” Those who responded no, along with those who identified as Democrat or closer to the Democratic Party, were asked, “Do you think of yourself as a supporter of the MAGA movement?” with response options “yes” and “no.” Respondents were then classified as MAGA Republicans, Republican MAGA supporters, non-MAGA Republicans, non-Republican MAGA supporters, and non-MAGA non-Republicans.

Our outcomes of primary interest concerned respondents’ support for and willingness to commit political violence. Violence was represented in the questionnaire by the phrase “force or violence,” defined as “physical force strong enough that it could cause pain or injury to a person.” “Force or violence to advance an important political objective that you support” was used to represent political violence.

Respondents were asked about the extent to which they considered political violence to be justified “in general” and then about justification for its use to advance each of 21 specified political objectives. Example objectives include “to stop people who do not share my beliefs from voting,” “to preserve the American way of life l believe in,” and “to support the right to life.”

Respondents who considered political violence to be at least sometimes justified to advance at least 1 objective were asked about their personal willingness to commit political violence: by type of violence (to “damage property,” “threaten or intimidate a person,” “injure a person,” and “kill a person”), by target population (examples: “an elected federal or state government official,” “a police officer,” and “a person who does not share your religion”), by social context (examples: “on your own” and “as part of a group”), and to ensure that their preferred candidate for President took office following the 2024 election.

All respondents were asked about the likelihood of their future use of firearms in a situation where they considered political violence to be justified (examples: “I will be armed with a gun” and “I will shoot someone with a gun”).

All respondents were also presented several items addressing their views on the potential need for violence to effect social change and on the likelihood of and need for civil war in the United States.

Details on the construction of measures for the additional characteristics listed in the introduction have been reported previously [16, 17, 22, 31, 33] and are described in the Supplement (see Additional File 1).

All questionnaire items that provided data for this analysis are in the Supplement (see Additional File 1). All data for principal exposures and outcome measures were collected in 2024. Data for some additional variables were collected in 2023 or 2022 (see the Supplement, Additional File 1).

### Implementation

Ipsos translated the questionnaire into Spanish, and interpreting services staff at UC Davis Medical Center reviewed the translation. Twenty-three KnowledgePanel members participated in a pretest of the English language version that was administered May 10–14, 2024.

Respondents were randomized 1:1 to receive response options in order from either negative to positive valence (example: from ‘do not agree’ to ‘strongly agree’) or the reverse throughout the questionnaire. Where a question presented multiple statements for respondents to consider, the order in which those statements were presented was randomized unless ordering was necessary. Logic-driving questions (those to which responses might invoke a skip pattern) included non-response prompts.

We employed unipolar response arrays without a neutral midpoint (for example, do not agree, somewhat agree, strongly agree, very strongly agree). The literature is not in agreement on whether such midpoints should be included [47, 48]. We were persuaded by the studies reviewed by Chyung et al. [47], which suggest that such midpoints allow respondents to choose “a minimally acceptable response as soon as it is found, instead of putting effort to find an optimal response,” a behavior known as satisficing. According to those authors, satisficing is particularly common when respondents are uncomfortable with the topics of the survey or under social desirability pressures; both conditions apply here. Our analyses focus on responses above the “somewhat” or “sometimes” level to minimize the impact of potential satisficing on the results.

### Statistical analysis

Analyses were conducted in R version 4.3.0. Information on procedures used to classify respondents on the violence-associated characteristics listed previously is given in the Supplement (see Additional File 1). To generate prevalence estimates, we calculated weighted percentages and 95% CIs using the *survey* and *srvyr* packages. We employed linear regression models to compute adjusted weighted prevalence differences (aPDs) and their 95% CIs, defining outcomes dichotomously and employing robust standard errors to correct for design effects and heteroskedasticity in binary outcomes. aPDs are absolute percentage point (pp) differences between estimates; for example, an aPD of 30pp might reflect the difference between prevalences of 10% and 40%. Variables were chosen for inclusion in the models based on concordance with theory and findings from prior research and included age, race and ethnicity, gender, education, income, Census division, rurality, military history, number of non-traffic arrests, and total number of drinks per week (see Supplement, Additional File 1).

All reported p-values were adjusted for multiple comparisons by controlling the false discovery rate (FDR) using the Benjamini-Hochberg method [49]. The resulting values are known as FDR-adjusted (or FDR-corrected) p-values or as q-values [50]; we employ the latter term here. Q values represent the probability that the given difference would be a false discovery; they represent the expected proportion of “false positives” that would be seen among the collection of all differences whose q-values were at or below the given q-value.

The survey was in the field when Donald Trump was convicted on 34 felony charges in New York State Supreme Court at approximately 5 PM Eastern Daylight Time on May 30, 2024 [51]. In a sensitivity analysis, we added a term for the interaction between MAGA affiliation and timing of questionnaire completion (pre- versus post-conviction) to the models used to calculate aPDs for political violence questionnaire items and assessed the significance of the interaction term, again adjusting p-values for multiple comparisons.

## Results

The primary analytic sample comprised 8896 respondents; the overall completion rate for Wave 3 was 88.4%. (Of 8932 Wave 2 respondents who were invited to participate, 8185 completed the survey; their completion rate was 91.6%. Of 1132 Wave 1 respondents who had not responded to Wave 2 and were invited to participate, 711 completed the survey, yielding a completion rate of 62.8%.) The median survey completion time was 22 min (interquartile range, 15.7 min). Item non-response in this analysis ranged from 0.01% to 3.4%; only 3 of 122 items had non-response percentages above 3.0% (see Supplement, Additional File 1).

After weighting, half of the respondents (50.9%, 95% CI 49.5%, 52.3%) were female; 62.7% (95% CI 61.2%, 64.2%) were white, non-Hispanic (Table S1). The weighted mean (SD) respondent age was 48.5 (24.9) years. Compared with nonrespondents, respondents were older; more frequently male, non-Hispanic white, and married; and less frequently working full-time (Table S2).

MAGA Republicans accounted for 11.8% of respondents (*n* = 1346, 95% CI 11.0%, 12.6%), other Republican supporters of the MAGA movement for 5.2% (*n* = 646, 95% CI 4.7%, 5.8%), non-MAGA Republicans for 18.1% (*n* = 1722, 95% CI 17.1%, 19.2%), non-Republican supporters of the MAGA movement for 2.7%% (*n* = 222, 95% CI 2.2%, 3.2%), and non-MAGA non-Republicans for 58.7% (*n* = 4731, 95% CI 57.3%, 60.1%). Another 3.5% (*n* = 229, 95% CI 2.9%, 4.0%) of respondents could not be classified (Table S3).

The primary comparison for this analysis is between MAGA Republicans and non-MAGA non-Republicans. Compared with non-MAGA non-Republicans, MAGA Republicans were older; more frequently male, white non-Hispanic, and married; less likely to have completed 4 years of college or obtained an advanced degree; less likely to be employed; more likely to live in the South Atlantic states; and less likely to live on the Pacific Coast (Table S3).

### Political violence

#### Violence to effect social change and civil war

By wide margins (Table 1), MAGA Republicans were more likely than non-MAGA non-Republicans to agree strongly or very strongly that force or violence might be justified under three sets of circumstances: (1) to “protect American democracy…if elected leaders will not,” (MAGA Republicans, 21.1% (95% CI 18.1%, 24.1%); non-MAGA non-Republicans, 7.3% (95% CI 6.2%, 8.4%); aPD 15.7pp (95% CI 12.3pp, 19.2pp), q < 0.001), (2) to save “our American way of life” that is “disappearing so fast” (MAGA Republicans, 27.2% (95% CI 23.9%, 30.6%); non-MAGA non-Republicans, 7.9% (95% CI 6.8%, 9.0%); aPD 20.6pp (95% CI 16.9pp, 24.2pp), q < 0.001), and (3) to “save our country” because “things have gotten so far off track,” a task for “true American patriots” (MAGA Republicans, 17.0% (95% CI 14.2%, 19.8%); non-MAGA non-Republicans, 5.4% (95% CI 4.5%, 6.4%); aPD 13.6pp (95% CI 10.4pp, 16.7pp) q < 0.001).

**Table 1:** MAGA affiliation and beliefs concerning violence to effect social change and civil war

Statement	Non-MAGA, Non-Republican		MAGA Republican				MAGA Supporter, Republican			
	Unweighted *n*	Weighted % (95% CI)	Unweighted *n*		Weighted % (95% CI)		Unweighted *n*		Weighted % (95% CI)	
If elected leaders will not protect American democracy, the people must do it themselves, even if it requires taking violent actions										
Do not agree	3450	70.1 (68.2,71.9)	534		41.6 (38.0,45.2)		290		48.4 (43.1,53.8)	
Somewhat agree	977	21.3 (19.7,23.0)	506		36.0 (32.6,39.4)		256		33.0 (28.3,37.6)	
Strongly or very strongly agree	268	7.3 (6.2,8.4)	296		21.1 (18.1,24.1)		92		16.1 (11.8,20.3)	
aPD (95% CI; q-value)	Referent		15.7 (12.3,19.2; <0.001)				10.5 (6.1,14.9; <0.001)			
Our American way of life is disappearing so fast that we may have to use force to save it										
Do not agree	3645	73.5 (71.7,75.3)	428		31.1 (27.8,34.4)		282		42.7 (37.4,48.0)	
Somewhat agree	763	17.5 (15.9,19.0)	545		40.7 (37.1,44.2)		269		40.1 (35.0,45.2)	
Strongly or very strongly agree	289	7.9 (6.8,9.0)	364		27.2 (23.9,30.6)		89		15.7 (11.4,20.1)	
aPD (95% CI; q-value)	Referent		20.6 (16.9,24.2; <0.001)				10.8 (6.4,15.2; <0.001)			
Because things have gotten so far off track, true American patriots may have to resort to violence in order to save our country										
Do not agree	4037	80.7 (79.1,82.4)	639		47.7 (44.1,51.3)		393		59.5 (54.2,64.8)	
Somewhat agree	485	12.7 (11.3,14.1)	457		34.1 (30.6,37.6)		193		27.9 (23.4,32.4)	
Strongly or very strongly agree	175	5.4 (4.5,6.4)	238		17.0 (14.2,19.8)		51		10.0 (6.2,13.8)	
aPD (95% CI; q-value)	Referent		13.6 (10.4,16.7; <0.001)				7.1 (3.2,11.0; 0.002)			
In the next few years, there will be civil war in the United States										
Do not agree	3178	65.3 (63.4,67.1)	712		52.1 (48.5,55.8)		438		63.8 (58.3,69.3)	
Somewhat agree	1324	28.1 (26.4,29.9)	496		36.3 (32.8,39.7)		174		29.2 (23.9,34.5)	
Strongly or very strongly agree	190	5.4 (4.5,6.4)	124		10.4 (7.9,12.9)		30		5.8 (3.2,8.4)	
aPD (95% CI; q-value)	Referent		6.8 (4.0,9.6; <0.001)				2.7 (−0.3,5.6; 0.25)			
The United States needs a civil war to set things right										
Do not agree	4411	90.5 (89.2,91.7)	967		72.1 (68.9,75.3)		543		80.9 (76.1,85.7)	
Somewhat agree	218	6.1 (5.1,7.2)	272		19.2 (16.5,21.9)		80		14.4 (10.1,18.7)	
Strongly or very strongly agree	68	2.4 (1.7,3.0)	96		7.8 (5.7,10.0)		15		2.4 (1.0,3.9)	
aPD (95% CI; q-value)	Referent		7.0 (4.6,9.4; <0.001)				1.7 (−0.1,3.4; 0.20)			

Statement	MAGA Supporter, Non-Republican		Non-MAGA, Republican				Unclassified				
	Unweighted *n*	Weighted % (95% CI)		Unweighted *n*		Weighted % (95% CI)		Unweighted *n*		Weighted % (95% CI)	
If elected leaders will not protect American democracy, the people must do it themselves, even if it requires taking violent actions											
Do not agree	81	37.8 (28.9,46.6)		1075		62.2 (59.0,65.3)		116		44.3 (36.0,52.6)	
Somewhat agree	80	33.6 (25.2,42.0)		457		26.4 (23.5,29.3)		34		14.7 (8.7,20.8)	
Strongly or very strongly agree	59	28.0 (19.2,36.8)		171		9.5 (7.6,11.4)		15		6.5 (2.5,10.6)	
aPD (95% CI; q-value)	19.1 (10.4,27.8; <0.001)			4.0 (1.7,6.3; 0.004)				1.8 (−4.2,7.9; 0.70)			
Our American way of life is disappearing so fast that we may have to use force to save it											
Do not agree	90	42.9 (33.7,52.0)		1050		58.7 (55.6,61.9)		88		31.2 (23.9,38.5)	
Somewhat agree	70	28.4 (20.5,36.2)		479		27.7 (24.8,30.5)		58		23.1 (16.1,30.1)	
Strongly or very strongly agree	61	28.4 (19.7,37.0)		181		12.2 (9.9,14.5)		21		12.5 (6.0,19.0)	
aPD (95% CI; q-value)	18.2 (9.5,27.0; <0.001)			6.8 (4.1,9.5; <0.001)				8.6 (−0.9,18.0; 0.19)			
Because things have gotten so far off track, true American patriots may have to resort to violence in order to save our country											
Do not agree	116	51.8 (42.6,61.0)		1272		71.5 (68.5,74.5)		121		46.0 (37.7,54.4)	
Somewhat agree	53	19.7 (13.1,26.3)		320		18.8 (16.3,21.4)		31		14.2 (8.0,20.4)	
Strongly or very strongly agree	52	28.1 (19.2,36.9)		114		8.2 (6.1,10.2)		12		4.7 (1.5,7.9)	
aPD (95% CI; q-value)	21.1 (12.2,29.9; <0.001)			4.5 (2.2,6.8; <0.001)				−0.0 (−4.7,4.7; 0.99)			
In the next few years, there will be civil war in the United States											
Do not agree	122	49.6 (40.4,58.8)		1221		66.1 (63.0,69.2)		97		46.0 (37.7,54.4)	
Somewhat agree	54	24.4 (16.6,32.2)		423		27.4 (24.5,30.3)		53		14.2 (8.0,20.4)	
Strongly or very strongly agree	43	25.1 (16.5,33.7)		69		5.7 (4.1,7.4)		9		4.7 (1.5,7.9)	
aPD (95% CI; q-value)	17.6 (9.1,26.2; <0.001)			2.1 (0.0,4.1; 0.20)				−1.6 (−6.6,3.5; 0.78)			
The United States needs a civil war to set things right											
Do not agree	155	67.2 (58.3,76.2)		1528		85.2 (82.7,87.7)		141		52.9 (44.4,61.5)	
Somewhat agree	34	13.3 (7.4,19.3)		147		10.8 (8.6,13.1)		15		6.4 (2.1,10.7)	
Strongly or very strongly agree	31	18.7 (10.6,26.8)		38		3.1 (1.8,4.3)		6		2.9 (0.1,5.8)	
aPD (95% CI; q-value)	14.6 (6.8,22.4; 0.003)			2.1 (0.5,3.6; 0.08)				1.7 (−3.0,6.3; 0.71)			

Adjusted prevalence differences (aPDs) are absolute percentage point (pp) differences for “strongly or very strongly agree” responses and are adjusted for age, race and ethnicity, gender, education, income, Census division, rurality, military history, number of non-traffic arrests, and total number of drinks per week. Q-values, also known as FDR-adjusted (or FDR-corrected) p-values, represent the probability that the given difference would be a false discovery; they represent the expected proportion of “false positives” that would be seen among the collection of all differences whose q-values were at or below the given q-value. Item non-responses are not reported in the tables but are included in the prevalence calculations

They were also more likely to agree strongly or very strongly (Table 1) that “in the next few years, there will be civil war in the United States” (MAGA Republicans, 10.4% (95% CI 7.9%, 12.9%); non-MAGA non-Republicans, 5.4% (95% CI 4.5%, 6.4%); aPD 6.8pp (95% CI 4.0pp, 9.6pp), q < 0.001) and that “the United States needs a civil war to set things right” (MAGA Republicans, 7.8% (95% CI 5.7%, 10.0%); non-MAGA non-Republicans, 2.4% (95% CI 1.7%, 3.0%); aPD 7.0pp (95% CI 4.6pp, 9.4pp), q < 0.001).

#### Political violence in general and to advance specific objectives

MAGA Republicans were substantially more likely than non-MAGA non-Republicans to consider violence to be usually or always justified for at least 1 of 21 specific political objectives (MAGA Republicans, 55.9% (95% CI 52.3%, 59.4%); non-MAGA non-Republicans, 25.5% (95% CI 23.7%, 27.2%); aPD 30.1pp (95% CI 26.0pp, 34.2pp), q < 0.001) and for 14 of those 21 objectives considered individually (Table 2, Table S4). For those 14 objectives, the prevalence of usually/always justification for violence among MAGA Republicans ranged from 7.1% (95% CI 4.8%, 9.3%) for “to oppose the government when it does not share my beliefs” to 30.4% (95% CI 27.2%, 33.6%) for “to stop illegal immigration.”

**Table 2:** MAGA affiliation and justification for political violence “in general” and to advance specific political objectives

What do you think about the use of force or violence in the following situations?	Non-MAGA, Non-Republican		MAGA Republican				MAGA Supporter, Republican			
	Unweighted *n*	Weighted % (95% CI)	Unweighted *n*		Weighted % (95% CI)		Unweighted *n*		Weighted % (95% CI)	
In general…to advance an important political objective that you support										
Never justified	3916	79.0 (77.3,80.7)	997		71.9 (68.4,75.3)		478		73.2 (68.3,78.1)	
Sometimes justified	749	18.6 (17.0,20.2)	322		25.0 (21.7,28.3)		161		25.7 (20.9,30.6)	
Usually or always justified	52	2.0 (1.3,2.6)	26		3.0 (1.5,4.6)		5		0.8 (−0.2,1.8)	
aPD (95% CI; q-value)	Referent		1.4 (0.0,2.9; 0.25)				−0.1 (−1.4,1.3; 0.95)			
Usually or always justified to advance at least 1 of 21 objectives	1151	25.5 (23.7,27.2)	771		55.9 (52.3,59.4)		313		45.7 (40.3,51.0)	
aPD (95% CI; q-value)	Referent		30.1 (26.0,34.2; <0.001)				21.6 (15.9,27.4; <0.001)			
Number of objectives for which violence is usually or always justified (mean, SD)	4.5 (4.2,4.7)		7.5 (7.0,8.0)				6.5 (5.9,7.1)			
To return Donald Trump to the presidency this year [in 2024]										
Never justified	4490	93.2 (92.1,94.3)	1042		74.5 (71.1,77.9)		565		84.7 (80.2,89.3)	
Sometimes justified	131	4.2 (3.3,5.2)	122		11.6 (8.8,14.3)		40		7.9 (4.4,11.4)	
Usually or always justified	77	2.0 (1.4,2.5)	170		12.9 (10.4,15.4)		38		6.5 (3.6,9.5)	
aPD (95% CI; q-value)	Referent		10.9 (8.3,13.5; <0.001)				6.0 (2.8,9.1; 0.003)			
To stop an election from being stolen										
Never justified	3958	83.0 (81.5,84.5)	758		57.0 (53.4,60.6)		409		63.0 (57.8,68.2)	
Sometimes justified	541	12.0 (10.7,13.3)	359		25.4 (22.2,28.6)		178		27.1 (22.5,31.6)	
Usually or always justified	199	4.5 (3.7,5.3)	219		16.4 (13.6,19.3)		56		9.1 (5.6,12.7)	
aPD (95% CI; q-value)	Referent		12.0 (9.0,15.0; <0.001)				5.8 (2.2,9.4; 0.02)			
To stop people who do not share my beliefs from voting										
Never justified	4536	93.8 (92.7,94.9)	1242		89.6 (86.8,92.4)		624		95.8 (93.3,98.3)	
Sometimes justified	112	3.7 (2.8,4.5)	60		5.7 (3.6,7.8)		16		3.2 (1.2,5.2)	
Usually or always justified	56	2.1 (1.4,2.7)	36		4.2 (2.2,6.1)		3		0.2 (−0.1,0.4)	
aPD (95% CI; q-value)	Referent		2.4 (0.6,4.3; 0.08)				−0.4 (−1.1,0.2; 0.36)			
To prevent discrimination based on race or ethnicity										
Never justified	3485	71.1 (69.3,72.9)	995		71.8 (68.4,75.3)		487		74.1 (69.1,79.1)	
Sometimes justified	948	21.4 (19.8,23.0)	242		19.3 (16.3,22.3)		126		21.1 (16.4,25.8)	
Usually or always justified	266	7.0 (6.0,8.1)	102		8.4 (6.1,10.7)		28		3.8 (2.0,5.6)	
aPD (95% CI; q-value)	Referent		3.0 (0.5,5.5; 0.09)				−0.9 (−3.1,1.4; 0.74)			
To preserve an American way of life based on Western European traditions										
Never justified	4069	85.5 (84.0,86.9)	735		57.9 (54.3,61.4)		388		61.2 (56.0,66.5)	
Sometimes justified	514	11.1 (9.8,12.4)	441		30.5 (27.2,33.9)		199		28.2 (23.7,32.8)	
Usually or always justified	110	2.7 (2.0,3.4)	163		11.1 (8.8,13.3)		55		9.5 (5.9,13.2)	
aPD (95% CI; q-value)	Referent		8.0 (5.8,10.2; <0.001)				7.5 (3.9,11.1; <0.001)			
To preserve the American way of life l believe in										
Never justified	3623	77.3 (75.7,78.9)	584		46.9 (43.3,50.6)		322		53.0 (47.7,58.4)	
Sometimes justified	860	17.2 (15.7,18.7)	521		35.3 (32.0,38.7)		250		35.6 (30.6,40.6)	
Usually or always justified	213	4.9 (4.1,5.7)	234		17.0 (14.3,19.8)		69		10.3 (7.1,13.4)	
aPD (95% CI; q-value)	Referent		12.1 (9.2,15.0; <0.001)				7.3 (3.8,10.7; <0.001)			
To oppose Americans who do not share my beliefs										
Never justified	4414	90.7 (89.4,91.9)	1185		86.1 (83.3,88.9)		595		91.3 (88.1,94.4)	
Sometimes justified	215	6.5 (5.4,7.6)	113		9.5 (7.0,12.0)		41		6.4 (3.9,8.8)	
Usually or always justified	71	2.3 (1.7,3.0)	43		3.9 (2.5,5.2)		6		1.4 (0.0,2.9)	
aPD (95% CI; q-value)	Referent		2.8 (1.1,4.4; 0.03)				0.7 (−1.1,2.4; 0.65)			
To oppose the government when it does not share my beliefs										
Never justified	4166	84.9 (83.3,86.4)	987		71.0 (67.5,74.5)		519		77.5 (72.6,82.3)	
Sometimes justified	453	12.5 (11.0,13.9)	277		21.0 (17.9,24.0)		110		18.5 (14.1,22.8)	
Usually or always justified	82	2.2 (1.6,2.8)	70		7.1 (4.8,9.3)		14		3.3 (1.0,5.6)	
aPD (95% CI; q-value)	Referent		5.6 (3.3,7.8; <0.001)				2.2 (−0.3,4.8; 0.35)			
To oppose the government when it tries to take private land for public purposes										
Never justified	3530	72.4 (70.7,74.2)	620		45.3 (41.7,48.9)		320		50.7 (45.4,56.1)	
Sometimes justified	961	21.4 (19.8,23.1)	486		35.4 (32.0,38.9)		245		35.0 (30.0,40.0)	
Usually or always justified	207	5.6 (4.6,6.5)	230		18.4 (15.4,21.5)		78		13.4 (9.4,17.5)	
aPD (95% CI; q-value)	Referent		14.0 (10.8,17.1; <0.001)				9.9 (5.7,14.1; <0.001)			

What do you think about the use of force or violence in the following situations?	MAGA Supporter, Non-Republican		Non-MAGA, Republican				Unclassified				
	Unweighted *n*	Weighted % (95% CI)		Unweighted *n*		Weighted % (95% CI)		Unweighted *n*		Weighted % (95% CI)	
In general…to advance an important political objective that you support											
Never justified	148	64.9 (56.0,73.8)		1435		80.6 (77.9,83.2)		173		66.3 (57.9,74.7)	
Sometimes justified	57	24.6 (16.8,32.3)		274		18.0 (15.4,20.5)		20		10.6 (5.3,15.9)	
Usually or always justified	16	10.1 (3.7,16.5)		8		0.8 (0.2,1.5)		1		1.2 (−1.1,3.6)	
aPD (95% CI; q-value)	7.4 (1.0,13.7; 0.21)			−0.3 (−1.3,0.8; 0.81)				−0.7 (−4.1,2.7; 0.83)			
Usually or always justified to advance at least 1 of 21 objectives	122	52.1 (42.9,61.3)		585		33.6 (30.6,36.7)		75		23.8 (17.3,30.3)	
aPD (95% CI; q-value)	23.4 (13.8,33.0; <0.001)			10.3 (6.8,13.9; <0.001)				−2.6 (−10.0,4.9; 0.57)			
Number of objectives for which violence is usually or always justified (mean, SD)	7.5 (6.1,9.0)			5.3 (4.9,5.7)				3.9 (2.9,4.8)			
To return Donald Trump to the presidency this year [in 2024]											
Never justified	176	76.2 (68.3,84.1)		1553		87.6 (85.3,89.9)		151		76.6 (68.2,84.9)	
Sometimes justified	16	9.1 (4.3,13.9)		75		5.9 (4.2,7.6)		11		8.0 (2.0,14.0)	
Usually or always justified	28	14.1 (7.1,21.0)		79		5.6 (4.0,7.2)		8		3.4 (0.5,6.4)	
aPD (95% CI; q-value)	9.5 (2.7,16.4; 0.04)			4.6 (2.9,6.3; <0.001)				1.0 (−2.5,4.6; 0.85)			
To stop an election from being stolen											
Never justified	136	65.7 (57.1,74.2)		1322		76.8 (74.1,79.5)		139		70.0 (61.2,78.9)	
Sometimes justified	51	17.4 (10.5,24.2)		284		16.7 (14.4,19.1)		20		11.2 (4.6,17.7)	
Usually or always justified	33	16.3 (10.0,22.6)		106		6.1 (4.6,7.6)		11		6.5 (1.9,11.0)	
aPD (95% CI; q-value)	9.4 (3.2,15.6; 0.02)			2.6 (0.9,4.3; 0.02)				2.4 (−3.1,7.9; 0.56)			
To stop people who do not share my beliefs from voting											
Never justified	195	83.7 (76.1,91.2)		1652		94.4 (92.6,96.1)		166		82.0 (74.3,89.7)	
Sometimes justified	8	6.9 (0.7,13.1)		43		3.5 (2.1,4.9)		5		5.0 (0.2,9.7)	
Usually or always justified	17	8.8 (3.9,13.6)		18		1.9 (0.9,3.0)		3		1.3 (−0.3,3.0)	
aPD (95% CI; q-value)	5.0 (0.1,9.8; 0.17)			0.9 (−0.3,2.1; 0.26)				−1.3 (−3.5,0.9; 0.41)			
To prevent discrimination based on race or ethnicity											
Never justified	143	62.2 (53.3,71.1)		1327		75.6 (72.8,78.4)		143		70.5 (61.7,79.2)	
Sometimes justified	48	16.9 (11.2,22.5)		304		18.8 (16.2,21.4)		23		14.9 (7.9,21.9)	
Usually or always justified	29	20.3 (12.1,28.4)		82		5.3 (3.9,6.8)		6		3.4 (0.4,6.3)	
aPD (95% CI; q-value)	10.4 (2.4,18.5; 0.06)			0.3 (−1.5,2.1; 0.83)				−3.0 (−6.8,0.8; 0.39)			
To preserve an American way of life based on Western European traditions											
Never justified	136	64.5 (55.7,73.2)		1252		74.8 (72.0,77.5)		143		73.6 (65.3,82.0)	
Sometimes justified	58	23.8 (15.9,31.7)		372		19.3 (16.9,21.7)		24		13.1 (6.6,19.5)	
Usually or always justified	25	11.0 (5.6,16.3)		85		5.4 (3.8,7.0)		5		2.1 (0.1,4.1)	
aPD (95% CI; q-value)	6.8 (1.5,12.1; 0.06)			3.4 (1.6,5.2; 0.002)				−1.1 (−3.6,1.4; 0.63)			
To preserve the American way of life l believe in											
Never justified	113	54.1 (44.9,63.2)		1121		67.3 (64.3,70.3)		113		61.8 (52.8,70.8)	
Sometimes justified	65	23.1 (16.2,30.1)		453		24.2 (21.5,26.9)		39		17.0 (10.0,23.9)	
Usually or always justified	42	22.7 (14.5,30.9)		135		8.0 (6.2,9.8)		21		10.3 (5.2,15.4)	
aPD (95% CI; q-value)	15.5 (7.2,23.9; 0.002)			4.4 (2.4,6.4; <0.001)				5.2 (−0.8,11.2; 0.23)			
To oppose Americans who do not share my beliefs											
Never justified	185	79.2 (71.2,87.1)		1594		91.0 (89.0,93.0)		162		82.6 (75.2,90.0)	
Sometimes justified	18	9.0 (3.8,14.3)		88		6.0 (4.5,7.6)		12		7.1 (2.0,12.2)	
Usually or always justified	17	11.7 (5.0,18.4)		29		2.6 (1.4,3.9)		4		1.9 (−0.1,4.0)	
aPD (95% CI; q-value)	6.8 (0.2,13.3; 0.26)			1.7 (0.2,3.2; 0.20)				−0.5 (−3.1,2.0; 0.86)			
To oppose the government when it does not share my beliefs											
Never justified	156	71.7 (63.3,80.0)		1474		85.1 (82.7,87.4)		157		78.7 (70.7,86.8)	
Sometimes justified	43	14.1 (8.3,19.8)		199		11.3 (9.3,13.3)		13		10.0 (3.7,16.4)	
Usually or always justified	20	13.5 (6.4,20.5)		40		3.4 (2.0,4.9)		4		1.9 (−0.2,4.0)	
aPD (95% CI; q-value)	9.8 (2.9,16.7; 0.07)			2.4 (0.7,4.0; 0.07)				0.0 (−2.7,2.7; 0.99)			
To oppose the government when it tries to take private land for public purposes											
Never justified	112	53.0 (43.8,62.2)		1091		63.7 (60.6,66.8)		122		64.3 (55.4,73.3)	
Sometimes justified	62	22.1 (15.0,29.3)		499		27.7 (24.9,30.5)		42		19.8 (12.5,27.1)	
Usually or always justified	46	24.2 (16.1,32.3)		122		8.4 (6.5,10.2)		10		5.1 (1.1,9.1)	
aPD (95% CI; q-value)	16.9 (8.6,25.2; <0.001)			4.2 (2.1,6.4; <0.001)				0.3 (−4.6,5.2; 0.98)			

Adjusted prevalence differences (aPDs) are absolute percentage point (pp) differences for “usually or always justified” responses and are adjusted for age, race and ethnicity, gender, education, income, Census division, rurality, military history, number of non-traffic arrests, and total number of drinks per week. Q-values, also known as FDR-adjusted (or FDR-corrected) p-values, represent the probability that the given difference would be a false discovery; they represent the expected proportion of “false positives” that would be seen among the collection of all differences whose q-values were at or below the given q-value. Item non-responses are not reported in the tables but are included in the prevalence calculations

MAGA Republicans also considered violence usually or always justified for a greater average number of specific political objectives than non-MAGA non-Republicans did: MAGA Republicans, 7.5 (95% CI 7.0, 8.0); non-MAGA non-Republicans, 4.5 (95% CI 4.2, 4.7) (Table 2).

#### Personal willingness to commit political violence

Among respondents who considered violence usually or always justified to advance 1 or more political objectives, MAGA Republicans were not more likely than non-MAGA non-Republicans to report that they would be very or completely willing personally to damage property or threaten, injure, or kill a person to advance a political objective (Table 3), to commit political violence against any of 9 specified target populations (Table S5), or to commit political violence as an organizer or member of a group (Table S6). They were, however, more often very or completely willing to commit political violence on their own (MAGA Republicans, 6.9% (95% CI 4.8%, 9.0%); non-MAGA non-Republicans, 2.8% (95% CI 2.1%, 3.5%); aPD 5.7pp (95% CI 3.4pp, 8.1pp), q < 0.001) (Table S6) and to “make sure your preferred candidate for President takes office” in 2024 if “another candidate is declared the winner, and you believe the election is being stolen” (MAGA Republicans, 4.2% (95% CI 2.5%, 5.9%); non-MAGA non-Republicans, 1.3% (95% CI 0.9%, 1.8%); aPD 3.9pp (95% CI 2.1pp, 5.7pp), q = 0.001) (Table S7).

**Table 3:** MAGA affiliation and personal willingness to commit political violence, by type of violence

In a situation where you think force or violence is justified to advance an important political objective…How willing would *you personally* be to use force or violence in each of these ways?	Non-MAGA, Non-Republican			MAGA Republican				MAGA Supporter, Republican			
	Unweighted *n*	Weighted % (95% CI)		Unweighted *n*		Weighted % (95% CI)		Unweighted *n*		Weighted % (95% CI)	
To damage property											
Not asked the question	1423	33.0 (31.1,34.8)		128		11.6 (9.2,14.1)		65		12.4 (8.6,16.1)	
Not willing	2837	54.8 (52.9,56.8)		1093		77.2 (73.9,80.5)		520		77.5 (72.9,82.1)	
Somewhat willing	362	9.1 (7.9,10.3)		84		7.2 (4.9,9.5)		48		7.9 (5.2,10.7)	
Very or completely willing	96	2.7 (2.0,3.4)		31		2.7 (1.4,4.1)		10		1.1 (0.3,1.9)	
aPD (95% CI; q-value)	Referent			0.6 (−0.9,2.2; 0.80)				−1.0 (−2.1,0.0; 0.23)			
To threaten or intimidate a person											
Not asked the question	1423	33.0 (31.1,34.8)		128		11.6 (9.2,14.1)		65		12.4 (8.6,16.1)	
Not willing	2925	57.3 (55.4,59.2)		1032		74.4 (71.2,77.6)		496		72.5 (67.3,77.6)	
Somewhat willing	295	7.2 (6.2,8.3)		143		10.1 (7.9,12.3)		71		11.8 (7.9,15.7)	
Very or completely willing	71	2.1 (1.4,2.7)		32		2.6 (1.5,3.7)		10		1.9 (0.3,3.5)	
aPD (95% CI; q-value)	Referent			1.6 (0.2,2.9; 0.18)				0.9 (−0.9,2.8; 0.59)			
To injure a person											
Not asked the question	1423	33.0 (31.1,34.8)		128		11.6 (9.2,14.1)		65		12.4 (8.6,16.1)	
Not willing	3012	59.0 (57.1,60.9)		1085		77.2 (74.0,80.4)		511		75.0 (69.9,80.0)	
Somewhat willing	207	5.3 (4.4,6.3)		88		7.1 (5.0,9.1)		57		9.7 (6.0,13.4)	
Very or completely willing	72	2.2 (1.5,2.8)		33		2.8 (1.5,4.0)		9		1.6 (0.3,2.9)	
aPD (95% CI; q-value)	Referent			1.4 (−0.0,2.8; 0.23)				0.1 (−1.3,1.6; 0.90)			
To kill a person											
Not asked the question	1423	33.0 (31.1,34.8)		128		11.6 (9.2,14.1)		65		12.4 (8.6,16.1)	
Not willing	3101	61.3 (59.4,63.2)		1117		79.9 (76.8,83.0)		534		79.4 (74.9,84.0)	
Somewhat willing	133	3.4 (2.7,4.2)		62		4.8 (3.2,6.3)		34		4.6 (2.7,6.5)	
Very or completely willing	57	1.8 (1.2,2.4)		28		2.2 (0.8,3.5)		9		2.4 (0.4,4.3)	
aPD (95% CI; q-value)	Referent			1.0 (−0.5,2.4; 0.48)				1.1 (−1.1,3.3; 0.60)			

In a situation where you think force or violence is justified to advance an important political objective…How willing would *you personally* be to use force or violence in each of these ways?	MAGA Supporter, Non-Republican			Non-MAGA, Republican				Unclassified				
	Unweighted *n*		Weighted % (95% CI)		Unweighted *n*		Weighted % (95% CI)		Unweighted *n*		Weighted % (95% CI)	
To damage property												
Not asked the question	42		25.0 (16.7,33.3)		325		23.2 (20.4,26.0)		88		47.2 (38.7,55.7)	
Not willing	145		54.2 (44.9,63.5)		1257		68.2 (65.1,71.2)		126		46.9 (38.5,55.3)	
Somewhat willing	13		8.9 (2.6,15.1)		106		6.6 (4.9,8.3)		6		2.5 (0.3,4.7)	
Very or completely willing	19		10.0 (4.5,15.6)		30		1.9 (0.9,2.8)		2		0.3 (−0.1,0.6)	
aPD (95% CI; q-value)	6.0 (0.2,11.8; 0.20)				−0.3 (−1.5,0.9; 0.94)				−2.5 (−3.5,−1.5; <0.001)			
To threaten or intimidate a person												
Not asked the question	42		25.0 (16.7,33.3)		325		23.2 (20.4,26.0)		88		47.2 (38.7,55.7)	
Not willing	137		52.5 (43.2,61.7)		1241		67.6 (64.5,70.7)		123		45.3 (37.0,53.7)	
Somewhat willing	21		10.4 (4.1,16.7)		126		6.9 (5.2,8.6)		7		2.9 (0.5,5.3)	
Very or completely willing	19		10.3 (4.7,15.8)		25		2.1 (1.0,3.2)		4		1.5 (−0.1,3.0)	
aPD (95% CI; q-value)	6.9 (1.1,12.7; 0.18)				0.9 (−0.4,2.2; 0.50)				−0.8 (−2.8,1.2; 0.64)			
To injure a person												
Not asked the question	42		25.0 (16.7,33.3)		325		23.2 (20.4,26.0)		88		47.2 (38.7,55.7)	
Not willing	144		56.3 (47.0,65.6)		1265		69.4 (66.4,72.5)		129		47.8 (39.4,56.2)	
Somewhat willing	14		7.4 (1.8,13.0)		105		5.9 (4.3,7.5)		3		1.5 (−0.4,3.3)	
Very or completely willing	18		9.3 (3.9,14.6)		21		1.3 (0.5,2.1)		2		0.4 (−0.2,1.1)	
aPD (95% CI; q-value)	5.7 (0.1,11.3; 0.23)				−0.3 (−1.3,0.7; 0.80)				−2.2 (−3.5,−1.0; 0.01)			
To kill a person												
Not asked the question	42		25.0 (16.7,33.3)		325		23.2 (20.4,26.0)		88		47.2 (38.7,55.7)	
Not willing	148		57.3 (48.0,66.6)		1289		70.8 (67.8,73.8)		128		47.4 (39.0,55.8)	
Somewhat willing	14		9.6 (3.1,16.1)		72		3.7 (2.5,5.0)		3		0.6 (−0.2,1.3)	
Very or completely willing	14		5.8 (1.9,9.7)		28		2.0 (1.0,2.9)		.		. (.,.)	
aPD (95% CI; q-value)	3.0 (−1.0,7.1; 0.45)				0.7 (−0.5,1.9; 0.50)				−2.3 (−3.2,−1.4; <0.001)			

Adjusted prevalence differences (aPDs) are absolute percentage point (pp) differences for “very or completely willing” responses and are adjusted for age, race and ethnicity, gender, education, income, Census division, rurality, military history, number of non-traffic arrests, and total number of drinks per week. Q-values, also known as FDR-adjusted (or FDR-corrected) p-values, represent the probability that the given difference would be a false discovery; they represent the expected proportion of “false positives” that would be seen among the collection of all differences whose q-values were at or below the given q-value. Item non-responses are not reported in the tables but are included in the prevalence calculations

#### Possession and use of firearms in political violence

MAGA Republicans reported more often than did non-MAGA non-Republicans (Table 4) that, “in a [future] situation where you think force or violence is justified to advance an important political objective” it was very or extremely likely that they would be “armed with a gun” (MAGA Republicans, 19.8% (95% CI 17.0%, 22.6%); non-MAGA non-Republicans, 5.5% (95% CI 4.6%, 6.4%); aPD 16.4pp (95% CI 13.3pp, 19.5pp), q < 0.001) and would “carry a gun openly” (MAGA Republicans, 9.2% (95% CI 6.9%, 11.4%); non-MAGA non-Republicans, 3.3% (95% CI 2.5%, 4.2%); aPD 6.9pp (95% CI 4.5pp, 9.3pp), q < 0.001) but not that they would “threaten someone with a gun” or “shoot someone with a gun.”

**Table 4:** MAGA affiliation and future firearm possession and use when political violence is perceived as justified

Thinking now about the future and all the changes it might bring, how likely is it that you will use a gun in any of the following ways in the next few years—in a situation where you think force or violence is justified to advance an important political objective?	Non-MAGA, Non-Republican		MAGA Republican				MAGA Supporter, Republican			
	Unweighted *n*	Weighted % (95% CI)	Unweighted *n*		Weighted % (95% CI)		Unweighted *n*		Weighted % (95% CI)	
I will be armed with a gun										
Not likely	3970	84.2 (82.8,85.7)	774		59.6 (56.1,63.2)		411		65.0 (59.8,70.2)	
Somewhat likely	450	9.2 (8.1,10.4)	237		18.4 (15.5,21.4)		113		16.8 (12.6,21.0)	
Very or extremely likely	277	5.5 (4.6,6.4)	309		19.8 (17.0,22.6)		113		15.5 (11.9,19.0)	
aPD (95% CI; q-value)	Referent		16.4 (13.3,19.5; <0.001)				11.3 (7.5,15.1; <0.001)			
I will carry a gun openly, so that people know I am armed										
Not likely	4369	90.7 (89.4,92.0)	994		74.5 (71.2,77.8)		536		82.1 (77.6,86.6)	
Somewhat likely	208	4.8 (3.9,5.7)	194		14.2 (11.6,16.9)		64		9.6 (6.1,13.1)	
Very or extremely likely	117	3.3 (2.5,4.2)	132		9.2 (6.9,11.4)		37		5.5 (3.3,7.8)	
aPD (95% CI; q-value)	Referent		6.9 (4.5,9.3; <0.001)				3.8 (1.3,6.2; 0.02)			
I will threaten someone with a gun										
Not likely	4573	95.4 (94.4,96.3)	1266		92.8 (90.6,94.9)		623		95.2 (92.4,98.0)	
Somewhat likely	81	2.2 (1.6,2.9)	38		3.3 (1.9,4.7)		8		1.2 (0.3,2.1)	
Very or extremely likely	38	1.3 (0.8,1.9)	19		1.9 (0.5,3.3)		6		1.0 (−0.2,2.2)	
aPD (95% CI; q-value)	Referent		1.4 (−0.2,3.0; 0.38)				0.7 (−0.8,2.2; 0.63)			
I will shoot someone with a gun										
Not likely	4528	94.3 (93.4,95.3)	1226		90.5 (88.1,92.9)		613		94.0 (91.0,97.0)	
Somewhat likely	114	3.0 (2.3,3.7)	72		5.5 (3.6,7.4)		18		2.5 (1.0,4.0)	
Very or extremely likely	51	1.6 (1.0,2.1)	27		2.1 (0.8,3.4)		8		1.1 (−0.1,2.3)	
aPD (95% CI; q-value)	Referent		1.5 (−0.1,3.0; 0.43)				0.6 (−0.9,2.0; 0.82)			

Thinking now about the future and all the changes it might bring, how likely is it that you will use a gun in any of the following ways in the next few years—in a situation where you think force or violence is justified to advance an important political objective?	MAGA Supporter, Non-Republican		Non-MAGA, Republican				Unclassified				
	Unweighted *n*	Weighted % (95% CI)		Unweighted *n*		Weighted % 95% CI)		Unweighted *n*		Weighted % (95% CI)	
I will be armed with a gun											
Not likely	138	63.2 (54.0,72.3)		1245		76.2 (73.6,78.9)		150		58.8 (50.3,67.3)	
Somewhat likely	31	15.2 (7.6,22.7)		264		13.2 (11.1,15.3)		13		6.1 (1.7,10.5)	
Very or extremely likely	47	19.1 (11.9,26.3)		194		9.2 (7.4,10.9)		14		5.6 (1.7,9.4)	
aPD (95% CI; q-value)	12.1 (4.9,19.3; 0.005)			5.1 (3.1,7.1; <0.001)				3.4 (−2.1,9.0; 0.45)			
I will carry a gun openly, so that people know I am armed											
Not likely	164	75.6 (67.7,83.6)		1478		86.6 (84.4,88.8)		160		63.8 (55.4,72.2)	
Somewhat likely	22	8.7 (4.1,13.3)		162		8.4 (6.7,10.2)		9		3.5 (0.1,6.9)	
Very or extremely likely	30	13.1 (6.6,19.7)		65		3.4 (2.3,4.6)		9		3.4 (0.4,6.4)	
aPD (95% CI; q-value)	6.8 (0.4,13.3; 0.18)			1.5 (0.0,2.9; 0.19)				1.2 (−3.0,5.5; 0.78)			
I will threaten someone with a gun											
Not likely	200	88.5 (81.8,95.2)		1667		96.0 (94.6,97.5)		169		66.8 (58.5,75.0)	
Somewhat likely	6	2.6 (0.2,5.0)		26		1.6 (0.7,2.5)		6		2.2 (−0.1,4.5)	
Very or extremely likely	11	6.8 (1.0,12.6)		14		1.2 (0.3,2.2)		1		0.3 (−0.3,1.0)	
aPD (95% CI; q-value)	3.7 (−1.8,9.1; 0.49)			0.8 (−0.4,2.0; 0.49)				−0.9 (−2.2,0.3; 0.45)			
I will shoot someone with a gun											
Not likely	194	85.5 (78.1,92.9)		1643		95.1 (93.6,96.6)		168		67.7 (59.5,75.9)	
Somewhat likely	8	4.1 (0.1,8.2)		47		2.5 (1.5,3.5)		5		1.4 (−0.6,3.5)	
Very or extremely likely	15	8.3 (2.3,14.2)		17		1.2 (0.3,2.2)		2		0.9 (−0.4,2.1)	
aPD (95% CI; q-value)	5.1 (−0.7,10.9; 0.46)			0.6 (−0.6,1.8; 0.82)				−0.4 (−2.4,1.5; 0.86)			

Adjusted prevalence differences (aPDs) are absolute percentage point (pp) differences for “very or extremely likely” responses and are adjusted for age, race and ethnicity, gender, education, income, Census division, rurality, military history, number of non-traffic arrests, and total number of drinks per week. Q-values, also known as FDR-adjusted (or FDR-corrected) p-values, represent the probability that the given difference would be a false discovery; they represent the expected proportion of “false positives” that would be seen among the collection of all differences whose q-values were at or below the given q-value. Item non-responses are not reported in the tables but are included in the prevalence calculations

### Characteristics associated with political violence

#### Democracy, authoritarianism, elections, and partisanship

In 2024, MAGA Republicans were more likely than non-MAGA non-Republicans (Table S8) to agree strongly or very strongly that “having a strong leader for America is more important than having a democracy” (MAGA Republicans, 28.1% (95% CI 24.8%, 31.4%); non-MAGA non-Republicans, 11.9% (95% CI 10.6%, 13.2%); aPD 16.8pp (95% CI 13.1pp, 20.4pp), q < 0.001) and that “we should suspend Congress for a few years so a strong leader can clean up the mess made by politicians in Washington” (MAGA Republicans, 26.0% (95% CI 22.7%, 29.2%); non-MAGA non-Republicans, 11.4% (95% CI 10.0%, 12.7%); aPD 15.7pp (95% CI 12.2pp, 19.3pp), q < 0.001).

Regarding elections, MAGA Republicans were far less likely than non-MAGA non-Republicans (Table S9) to view “making sure that everyone who wants to vote can do so” as more important than “making sure that no one votes who is not eligible to vote” (MAGA Republicans, 9.4% (95% CI 6.8%, 12.1%); non-MAGA non-Republicans, 69.4% (95% CI 67.6%, 71.1%); aPD − 57.7pp (95% CI −61.1pp, −54.2pp), q < 0.001). They were more likely to agree strongly or very strongly that “armed citizens should patrol polling places at election time” (MAGA Republicans, 17.4% (95% CI 14.3%, 20.4%); non-MAGA non-Republicans, 3.9% (95% CI 3.0%, 4.7%); aPD 15.3pp (95% CI 12.0pp, 18.6pp), q < 0.001). They were also more likely to agree strongly or very strongly that members of the other political party were “enemies—that is, if they win, your life or your entire way of life may be threatened” (MAGA Republicans, 55.9% (95% CI 52.2%, 59.5%); non-MAGA non-Republicans, 40.0% (95% CI 37.8%, 42.1%); aPD 15.5pp (95% CI 11.0pp, 19.9pp), q < 0.001).

#### Fear, hatred, and enmity toward others

MAGA Republicans were more likely than non-MAGA non-Republicans (Table S10) to rank in strong agreement on scales measuring homonegativity, racism, transphobia, xenophobia, hostile sexism, and Islamophobia. Prevalences of strong agreement among MAGA Republicans ranged from 16.3% (95% CI 13.3%, 19.2%) for hostile sexism to 69.9% (95% CI 66.5%, 73.4%) for homonegativity; aPDs exceeded 50pp for homonegativity and racism, and 30pp for transphobia and xenophobia. There was no difference in the prevalence of strong agreement on the scale measuring antisemitism (MAGA Republicans, 4.6% (95% CI 2.8%, 6.4%); non-MAGA non-Republicans, 3.7% (95% CI 2.9%, 4.5%); aPD 2.2pp (95% CI 0.2pp, 4.2pp), q = 0.10).

#### QAnon and Christian nationalism

MAGA Republicans were more likely than non-MAGA non-Republicans (Table S11) to agree strongly or very strongly with the central elements of the QAnon mythology, such as that US institutions “are controlled by a group of Satan-worshipping pedophiles who run a global child sex trafficking operation” (MAGA Republicans, 20.1% (95% CI 17.1%, 23.2%); non-MAGA non-Republicans, 6.8% (95% CI 5.6%, 7.9%); aPD 15.3pp (95% CI 12.0pp, 18.6pp), q < 0.001). They were also more likely than to agree strongly or very strongly with statements of Christian nationalist belief, such as “the U.S. government should declare America a Christian nation” (MAGA Republicans, 44.8% (95% CI 41.3%, 48.4%); non-MAGA non-Republicans, 9.0% (95% CI 7.8%, 10.2%); aPD 35.6pp (95% CI 31.7pp, 39.5pp), q < 0.001).

#### Conspiracism

MAGA Republicans were more likely than non-MAGA non-Republicans (Table S12) to agree strongly or very strongly with a wide range of conspiracy allegations, such as that “the government permits or perpetrates acts of terrorism on its own soil, disguising its involvement” (MAGA Republicans, 33.3% (95% CI 29.8%, 36.8%); non-MAGA non-Republicans, 10.6% (95% CI 9.2%, 11.9%); aPD 25.8pp (95% CI 21.8pp, 29.8pp), q < 0.001) and that “the spread of certain viruses and/or diseases is the result of the deliberate, concealed efforts of some organization” (MAGA Republicans, 40.2% (95% CI 36.4%, 43.9%); non-MAGA non-Republicans, 9.6% (95% CI 8.3%, 10.9%); aPD 33.2pp (95% CI 29.2pp, 37.3pp), q < 0.001).

#### Trait aggression

MAGA Republicans were somewhat more likely than non-MAGA non-Republicans (Table S13) to endorse statements of a propensity for violence, such as that “given enough provocation, I may hit another person” (MAGA Republicans, 12.2% (95% CI 9.9%, 14.6%); non-MAGA non-Republicans, 8.7% (95% CI 7.5%, 10.0%); aPD 5.7pp (95% CI 2.9pp, 8.5pp), q < 0.001) and that “there are people who pushed me so far that we came to blows” (MAGA Republicans, 7.6% (95% CI 5.4%, 9.8%); non-MAGA non-Republicans, 5.8% (95% CI 4.7%, 6.9%); aPD 3.4pp (95% CI 0.9pp, 5.8pp), q = 0.03). They were not more likely to agree strongly or very strongly with statements of grievance.

#### Non-political violence

MAGA Republicans were more likely than non-MAGA non-Republicans (Table S14) to view violence as usually or always justified in circumstances where that view was common (in self-defense, to prevent another person from injuring themselves or someone else, and to prevent damage to property) but not in circumstances where that view was uncommon (to win an argument, in response to an insult, or to get respect).

They were also more likely (Table S15) to agree strongly or very strongly that intimate partner violence was justified under a wide array of circumstances, including in response to actual or threatened violence by the partner, but not when “a partner forces you to have sex with him or her” (MAGA Republicans, 35.2% (95% CI 31.7%, 38.6%); non-MAGA non-Republicans, 36.6% (95% CI 34.8%, 38.5%); aPD − 0.3pp (95% CI −4.3pp, 3.7pp), q = 0.94).

#### Firearm ownership

MAGA Republicans were more likely than non-MAGA non-Republicans (Table S16) to own firearms (MAGA Republicans, 44.1% (95% CI 40.7%, 47.6%); non-MAGA non-Republicans, 17.1% (95% CI 15.9%, 18.2%); aPD 22.4pp (95% CI 18.8pp, 26.0pp), q < 0.001). Among firearm owners, MAGA Republicans were more likely to have purchased firearms in 2020 or later (MAGA Republicans, 45.0% (95% CI 40.6%, 49.3%); non-MAGA non-Republicans, 31.3% (95% CI 28.0%, 34.6%); aPD 18.4pp (95% CI 13.1pp, 23.8pp), q < 0.001).

### Other categories of respondents

Prevalences for other Republicans generally fell between those for MAGA Republicans and non-MAGA non-Republicans. We identified a small, demographically distinct group of non-Republican MAGA supporters (*n* = 222; 2.7% (95% CI 2.2%, 3.2%) whose levels of support for and willingness to commit political violence often exceeded those of MAGA Republicans (Tables 1, 2, 3 and 4, S4-S7). Further details on that group are in the Supplement (see Additional File 1).

### Sensitivity analysis

In the sensitivity analysis, the interaction term between MAGA affiliation and timing of questionnaire completion (pre- versus post-conviction) was statistically significant in 4 of 230 comparisons, 1 each among non-MAGA non-Republicans, non-MAGA Republicans, MAGA Republicans, and unclassified respondents.

## Discussion

The principal findings of this analysis are that in 2024, MAGA Republicans were substantially more likely than non-MAGA non-Republicans to endorse violence as a means of effecting social change and to view political violence as justified, but they were not more willing to commit political violence themselves. These results are consistent with those we obtained in 2022 [22] and with the findings of investigations by others [24–26]. Not surprisingly, across all groups, the view that political violence was justified was more common than was willingness personally to engage in it.

MAGA Republicans demonstrated often strikingly high prevalences of characteristics that have been associated with violence, including political violence, in this cohort and in other studies [16, 20, 31–35]. These include beliefs such as in QAnon and Christian nationalism and forms of fear, hatred and enmity toward others such as racism, homophobia, and xenophobia. They were substantially more likely than non-MAGA non-Republicans to endorse statements of authoritarianism, including a call to “suspend Congress for a few years” and the assertion that “having a strong leader for America is more important than having a democracy.” In the view of many experts, that preference may have been realized via the democratic process in 2024, with the US facing a real risk of transition to authoritarianism in 2025 [52].

It is clear from the findings that there is more to MAGAism than MAGA Republicanism. While 11.8% of respondents identified specifically as MAGA Republicans in this study, another 5.2% of respondents identified as Republican supporters of the MAGA movement. These 2 groups accounted for 33.6% of all Republican respondents in our cohort, and public opinion polling has yielded even higher estimates since the 2024 elections [53, 54].

Also of interest is the small group of non-Republicans who identified as MAGA supporters. This group was quite distinct demographically from MAGA Republicans: much younger, and much more female. These respondents were generally more supportive of violence than MAGA Republicans were and appeared to be more willing to commit violence personally (the small number of respondents in this category resulted in most differences on willingness being statistically nonsignificant). Others have also recently found young people to be particularly supportive of political violence [52]. Further research on this group is a priority, as is a broader inquiry into what distinguishes MAGA affiliates who will participate in political violence from those who will not.

The mixed findings for MAGA Republicans have mixed implications for efforts to prevent political violence. On one hand, those who see violence as justified in principle but are unwilling to do violence themselves might serve as credible messengers in efforts to dissuade others in the MAGA movement for whom political violence is a personal option. Such efforts might focus on the messengers’ immediate families and friends; a separate analysis of 2024 data from this cohort found that of respondents who thought it likely that they would serve as a combatant if civil war broke out, 44.5% would change their minds if urged to do so by their families, 23.4% if urged by their friends [55].

At the same time, the existence of a large group of people with a shared political identity and a shared belief that political violence is justified creates a climate of acceptance (if not encouragement) for political violence and raises the risk that it will occur. This suggests the need for interventions directed at reducing support for political violence among MAGA Republicans and others who believe that it can be justified—a necessary but tricky approach, as 2025 begins a long period of celebration in the US for the 250th anniversary of the American Revolution, the act of political violence that brought the country into being.

A key piece of information that could inform these efforts remains a matter for future research: Do MAGA Republicans who justify but will not participate in violence take that latter position because they think that violence is wrong, or simply because they would rather others do the work (and take the risks) for them?

### Limitations

The findings are cross-sectional and, weighting notwithstanding, are subject to sampling error and bias due to nonresponse, social desirability and other factors. To the extent that respondents viewed personal willingness to commit violence as stigmatized behavior, our results may reflect under-reporting. Some crosstabulations produced response counts < 100, and weighted prevalences for some political violence measures are below 5%, but the large study sample results in relatively narrow confidence intervals in these cases. Our political violence outcomes are proxy measures; we did not ask participants to provide information on their acts of political violence. The exposures of interest here were political party and MAGA movement affiliation. A separate study will examine the association between approval of right- and left-wing extremist groups and movements (such as the Proud Boys, the white supremacy movement, and the antifa movement) and political violence and will assess change from mid-2024 to mid-2025—before and after the 2024 presidential election. The survey was in the field when Donald Trump’s convictions on 34 felony charges in New York State Supreme Court were announced [46], but a sensitivity analysis indicated that this did not introduce additional bias. Another survey on political violence was in the field slightly later, at the time of an assassination attempt on then-candidate Donald Trump; it also found no effect on findings for political violence [23].

## Conclusion

Findings from this large, nationally representative survey demonstrate that MAGA Republicans are more likely than non-MAGA non-Republicans to endorse violence as a means of effecting social change and to view political violence as justified but not more willing to commit such violence themselves. They are more likely to endorse a wide array of forms of fear, hatred, and enmity toward others, including racism and xenophobia. Individual MAGA Republicans who are unwilling to commit violence might be credible messengers in efforts to dissuade others. At the same time, support for violence by persons unwilling to participate in it might have violence-promoting effects; efforts to diminish that support are merited as well.

## Supplementary Information

Supplementary Material 1

## Data Availability

The datasets generated and/or analyzed during the current study are not publicly available as analyses are continuing but will be made available to qualified researchers subject to the terms of a data use agreement.

## Abbreviations

aPD: Adjusted prevalence difference, expressed in percentage points (see Pp below)
CI: Confidence interval
Pp: Percentage point (used to denote absolute differences in percentages)
SD: Standard deviation

## References

[1] Kleinfeld R. The rise of political violence in the United States. J Democ. 2021;32(4):160–76.

[2] Kalmoe N, Mason L. Radical American partisanship: mapping violent hostility, its causes, and the consequences for democracy. Chicago: University of Chicago Press; 2022.

[3] Walter BF. How civil wars start and how to stop them. New York: Crown; 2022.

[4] Kleinfeld R. There won’t be a civil war… but the far right is pushing politics toward a darker and more violent future. Persuasion. 2022 Jul 15. https://www.persuasion.community/p/there-wont-be-a-civil-war

[5] Walter BF, Marche S, Parker CS. ‘These are conditions ripe for political violence’: how close is the US to civil war? The Guardian. 2022 Nov 6. https://www.theguardian.com/us-news/2022/nov/06/how-close-is-the-us-to-civil-war-barbara-f-walter-stephen-march-christopher-parker

[6] Piazza JA. Drivers of political violence in the United States. J Public Policy Mark. 2023;42(1):11–4.

[7] Simon S, Stevenson J. The threat of civil breakdown is real. Politico. 2023 Apr 21. https://www.politico.com/news/magazine/2023/04/21/political-violence-2024-magazine-00093028

[8] Kleinfeld R. Polarization, democracy, and political violence in the United States: what the research says. Carnegie Endowment for International Peace. 2023 Sep 5. https://carnegieendowment.org/2023/09/05/polarization-democracy-and-political-violence-in-united-states-what-research-says-pub-90457

[9] Clapman A. How States can prevent election subversion in 2024 and beyond. Brennan Center for Justice. 2023 September 6. https://www.brennancenter.org/our-work/research-reports/how-states-can-prevent-election-subversion-2024-and-beyond

[10] Morales-Doyle S, Sanders R, Anderman A, Ojeda J. Guns and voting: how to protect elections after Bruen. Brennan Center for Justice, New York University School of Law; Giffords Law Center to Prevent Gun Violence. 2023 September 18. or https://www.brennancenter.org/our-work/policy-solutions/guns-and-votinghttps://giffords.org/report/guns-and-voting-how-to-protect-elections-after-bruen/

[11] Carey T, Roskam K, Horwitz J, Johns Hopkins Bloomberg School of Public Health. Defending democracy: addressing the dangers of armed insurrection. Center for Gun Violence Solutions,. 2023 December 5. https://publichealth.jhu.edu/2023/preventing-armed-insurrection-firearms-in-political-spaces-threaten-public-health-safety-and-democracy

[12] Ramachandran G, Lee C, Kornberg M, Peeler-Allen K, Edlin R, Fishman J, Park J, Short GY. Intimidation of state and local officeholders. Brennan Center for Justice, New York University School of Law. 2024. https://www.brennancenter.org/our-work/research-reports/intimidation-state-and-local-officeholders

[13] Armed Conflict Location & Event Data Project (ACLED). Will past US election turbulence strike again in 2024? 2024 Mar 14. https://acleddata.com/2024/03/14/will-past-us-election-turbulence-strike-again-in-2024/

[14] Ware J, Preventing. U.S. Election violence in 2024: contingency planning memorandum. Council Foreign Relations. 2024 Apr 17. https://www.cfr.org/report/preventing-us-election-violence-2024

[15] Armed Conflict Location & Event Data Project (ACLED). Beyond the blue wall: exploring the risks of political unrest in the 2024 presidential election. 2024 Apr 17. https://acleddata.com/2024/04/17/beyond-the-blue-wall-exploring-the-risks-of-political-unrest-in-the-2024-presidential-election/

[16] Wintemute GJ, Robinson SL, Crawford A, Tancredi D, Schleimer JP, Tomsich EA, et al. Views of democracy and society and support for political violence in the USA: findings from a nationally representative survey. Inj Epidemiol. 2023;10(1):45. 37770994 10.1186/s40621-023-00456-3 PMC10540371

[17] Wintemute GJ, Crawford A, Robinson A, Schleimer JP, Tomsich EA, Pear VA. Party affiliation, political ideology, views of American democracy and society, and support for political violence: findings from a nationwide population-representative survey. SocArXiv. 2022 Oct 21. 10.31235/osf.io/n9b36.

[18] Piazza JA. Political polarization and political violence. Secur Stud. 2023;32(3):476–504.

[19] Lorenzano KJ, Moon SJ, Borah P. Partisan selective exposure and politically polarized attitudes toward disruptive protest. Commun Public. 2023;8(3):191–205.

[20] Armaly MT, Enders AM. Who supports political violence? Perspect Polit. 2024;22(2):427–44.

[21] Holliday DE, Iyengar S, Lelkes Y, Westwood SJ. Uncommon and nonpartisan: antidemocratic attitudes in the American public. Proc Natl Acad Sci U S A. 2024;121(13):e2313013121. 38498713 10.1073/pnas.2313013121 PMC10990094

[22] Wintemute GJ, Robinson R, Tomsich EA. MAGA republicans’ views of American democracy and society and support for political violence: findings from a nationwide population-representative survey. PLoS ONE. 2024;19(1):e0295747. 38170700 10.1371/journal.pone.0295747 PMC10763974

[23] Holliday DE, Lelkes Y, Westwood SJ. The July 2024 Trump assassination attempt was followed by lower in-group support for partisan violence and increased group unity. Proc Natl Acad Sci U S A. 2024;121(49):e2414689121. 39589876 10.1073/pnas.2414689121 PMC11626138

[24] Blum RM, Parker CS. Panel study of the MAGA movement. University of Washington. Undated. https://sites.uw.edu/magastudy/. Accessed Jul 11, 2024.

[25] Center for Communication & Civic Renewal, University of Wisconsin-Madison. Civic Fracture & Renewal in Wisconsin: A Report on the Public’s Civic Attitudes and Behaviors. 2023 Mar 31. https://journalism.wisc.edu/wp-content/blogs.dir/41/files/2023/03/2023-CCCR-Report-Civic-Fracture-Renewal-in-Wisconsin-For-Release.pdf

[26] Johnston H. The MAGA movement’s big umbrella. Mobilization: Int Q. 2023;28(4):409–33.

[27] The Economist/YouGov, Poll. November 26–29, 2022. https://today.yougov.com/topics/economy/articles-reports/2022/11/30/economy-economist-yougov-poll-november-26-29-2022

[28] Trump gains on DeSantis. Monmouth University Poll. 2023 Mar 21. https://www.monmouth.edu/polling-institute/reports/monmouthpoll_us_032123_2/

[29] Frankovic K, Montgomery D. Most Americans like NATO and say the U.S. should defend NATO allies. YouGov. 2024 Feb23. https://today.yougov.com/politics/articles/48744-most-americans-like-nato-say-us-should-defend-allies

[30] Perry S. We polled thousands of voters during the GOP primary season. Here’s what we learned. NBC News. 2024 Mar 20. https://www.nbcnews.com/politics/2024-election/gop-primary-exit-polls-trump-rcna144248

[31] Wintemute GJ, Velasquez B, Tomsich EA, Reeping PM, Robinson SL, Tancredi D, Pear VA. Fear, loathing, and political violence in the United States: findings from a nationally representative survey. The Lancet Regional Health—Americas. 2025;51:101235.32 41019518 10.1016/j.lana.2025.101235 PMC12464958

[32] Wintemute GJ, Velasquez B, Li Y, Tomsich EA, Reeping PM, Robinson SL. Racist and pro-violence beliefs, approval of extreme right-wing political organizations and movements, and support for political violence in the United States. [Preprint.] SocArXiv. 2023. Online publication Dec 4. 10.31235/osf.io/c9vtr

[33] Wintemute GJ, Crawford A, Robinson S, Tomsich E, Reeping P, Schleimer J, et al. Firearm ownership and support for political violence in the United States. JAMA Netw Open. 2024;7(4):e243623. 38592725 10.1001/jamanetworkopen.2024.3623 PMC11004826

[34] Diamond PM, Magaletta PR. The short-form Buss-Perry aggression questionnaire (BPAQ-SF): a validation study with federal offenders. Assessment. 2006;13(3):227–40. 16880276 10.1177/1073191106287666

[35] Brotherton R, French CC, Pickering AD. Measuring belief in conspiracy theories: the generic conspiracist beliefs scale. Front Psychol. 2013;4:279. 23734136 10.3389/fpsyg.2013.00279 PMC3659314

[36] Wintemute GJ, Robinson SL, Crawford A, Tomsich EA, Reeping PM, Shev AB, et al. Single-year change in views of democracy and society and support for political violence in the USA: findings from a 2023 nationally representative survey. Inj Epidemiol. 2024;11(1):20. 38773542 10.1186/s40621-024-00503-7 PMC11110245

[37] Ipsos. https://www.ipsos.com/en

[38] American Association for Public Opinion Research. Code of professional ethics and practices. (April 2021 edition). https://www.aapor.org/Standards-Ethics/AAPOR-Code-of-Ethics.aspx

[39] Kravitz-Wirtz N, Aubel A, Schleimer J, Pallin R, Wintemute G. Public concern about violence, firearms, and the COVID-19 pandemic in California. JAMA Netw Open. 2021;4(1):e2033484. 33394004 10.1001/jamanetworkopen.2020.33484 PMC7783542

[40] Wintemute GJ, Aubel AJ, Pallin RS, Schleimer JP, Kravitz-Wirtz N. Experiences of violence in daily life among adults in California: a population-representative survey. Inj Epidemiol. 2022;9:1. 10.1186/s40621-021-00367-1. 34980276 10.1186/s40621-021-00367-1 PMC8721630

[41] Schleimer J, Kravitz-Wirtz N, Pallin R, Charbonneau A, Buggs S, Wintemute G. Firearm ownership in California: a latent class analysis. Inj Prev. 2020;26(5):456–62. 31601624 10.1136/injuryprev-2019-043412

[42] Miller M, Zhang W, Azrael D. Firearm purchasing during the Covid-19 pandemic: results from the 2021 National Firearm Survey. Ann Intern Med. 2022;175(2):219–25. 34928699 10.7326/M21-3423 PMC8697522

[43] Miller M, Azrael D. Firearm storage in US households with children: findings from the 2021 National Firearm Survey. JAMA Netw Open. 2022;5(2):e2148823. 35191973 10.1001/jamanetworkopen.2021.48823 PMC8864510

[44] Salhi C, Azrael D, Miller M. Patterns of gun owner beliefs about firearm risk in relation to firearm storage: a latent class analysis using the 2019 National Firearm Survey. Inj Prev. 2020 Jul 14;injuryprev-2019-043624. 10.1136/injuryprev-2019-043624 32665253

[45] Ipsos. KnowledgePanel: a methodological overview. https://www.ipsos.com/sites/default/files/ipsosknowledgepanelmethodology.pdf

[46] Ipsos. KnowledgePanel sampling and weighting methodology. https://www.ipsos.com/sites/default/files/kpsamplingandweighting.pdf

[47] Chyung SY, Roberts K, Swanson I, Hankinson A. Evidence-based survey design: the use of a midpoint on the likert scale. Performance Improvement. 2017;56(10):15–23.

[48] Westwood SJ, Grimmer J, Tyler M, Nall C. Current research overstates American support for political violence. Proc Natl Acad Sci U S A. 2022;119(12):e2116870119. 35302889 10.1073/pnas.2116870119 PMC8944847

[49] Benjamini Y, Hochberg Y. Controlling the false discovery rate: a practical and powerful approach to multiple testing. J R Stat Soc Series B Stat Methodol. 1995;57(1):289–300.

[50] Storey JD. The positive false discovery rate: a bayesian interpretation and the q-value. Ann Statist. 2003;31(6):2013–35.

[51] Gamio L, Yourish K, Haag M, Bromwich JE, Haberman M, Lai KKR. The Trump Manhattan criminal verdict, count by count. N Y Times. 2024 May 30.

[52] Bright Line Watch. Accelerated transgressions in the second Trump presidency. February 2025. https://brightlinewatch.org/accelerated-transgressions-in-the-second-trump-presidency/

[53] Kamisar B, NBC News. Polling shows growing number of Republicans identify with the MAGA movement. 2025 Apr 14. https://www.nbcnews.com/politics/trump-administration/polling-shows-growing-number-republicans-identify-maga-movement-rcna201071

[54] The Vanderbilt Project on Unity and American Democracy. Majority of Republicans nationally identify as MAGA for first time in Unity Poll. 2025 Feb 24. https://news.vanderbilt.edu/2025/02/24/majority-of-republicans-nationally-identify-as-maga-for-first-time-in-unity-poll/

[55] Wintemute GJ, Li Y, Wright MA, Crawford A, Tomsich EA. Public opinion on civil war in the USA as of mid-2024: findings from a nationally representative survey. Inj Epidemiol. 2025;12:36. 40611307 10.1186/s40621-025-00594-w PMC12225159

