# Supplementary material for: The MAGA movement and political violence in 2024: findings from a nationally representative survey
Source: Inj Epidemiol. 2025 Nov 19;12:78. doi: 10.1186/s40621-025-00633-6 (PMC12628544; doi:10.1186/s40621-025-00633-6)
Supplement: Supplementary file 1 — Supplementary Material 1 [file 40621_2025_633_MOESM1_ESM.pdf]

Supplement

The MAGA Movement and Political Violence in 2024:

Findings from a Nationally Representative Survey

Garen J. Wintemute, MD, MPH; Bradley Velasquez, MS; Sonia L. Robinson, PhD, MPH; Elizabeth A. Tomsich, PhD; Mona A. Wright, MPH; Aaron B. Shev, PhD

This supplement has been provided by the authors to give readers additional information about the work.

Page	Title
3	Questions that supplied data for this study
17	Additional methods text
20	Additional results text
23	References
25	Figure S1. Flowchart of survey assignment and completion counts for Waves 1-3 in 2022-2024
26	Table S1. Sociodemographic characteristics (weighted) of Wave 3 respondents
28	Table S2. Sociodemographic characteristics (unweighted) of respondents and non-respondents
30	Table S3. Sociodemographic characteristics (weighted) of respondents by MAGA affiliation
34	Table S4. MAGA affiliation and justification for violence to advance additional specific political objectives
39	Table S5. MAGA affiliation and personal willingness to engage in political violence, by target of violence
43	Table S6. MAGA affiliation and personal willingness to commit political violence, by social context of violence
45	Table S7. MAGA affiliation and personal willingness to commit election-year violence
47	Table S8. MAGA affiliation and beliefs concerning democracy and authoritarianism
52	Table S9. MAGA affiliation and beliefs concerning elections and partisanship
55	Table S10. MAGA affiliation and specified forms of hatred, fear, and enmity toward others
59	Table S11. MAGA affiliation and endorsement of QAnon and Christian nationalism
63	Table S12. MAGA affiliation and conspiracism
66	Table S13. MAGA affiliation and trait aggression
69	Table S14. MAGA affiliation and justification for violence in non-political circumstances

Page	Title
73	Table S15. MAGA affiliation and justification for intimate partner violence
78	Table S16. MAGA affiliation and firearm ownership

QUESTIONS THAT SUPPLIED DATA FOR THIS STUDY

Response options are presented here in order from negative to positive (e.g., “not important” to “extremely important”). Respondents were randomized 1:1 to receive responses in that order or the reverse.

In the list below, questions or items that were repeated or adapted from prior surveys contain citations to those surveys.

Unless specified otherwise, data were collected in 2024.

Political violence

Violence to effect social change

Q: People have different views about violence in the United States. How much do you agree or disagree with each of the following statements?

a. If elected leaders will not protect American democracy, the people must do it themselves, even if it requires taking violent actions.[1]
b. Because things have gotten so far off track, true American patriots may have to resort to violence in order to save our country.[2]
c. Our American way of life is disappearing so fast that we may have to use force to save it.[1]

1. Do not agree
2. Somewhat agree
3. Strongly agree
4. Very strongly agree

Q. How much do you agree or disagree with each of the following statements?

a. In the next few years, there will be civil war in the United States.
b. The United States needs a civil war to set things right.

1. Do not agree
2. Somewhat agree
3. Strongly agree
4. Very strongly agree

Violence to advance political objectives

Now we have a few questions about the use of force or violence. “Force or violence” means physical force strong enough that it could cause pain or injury to a person. A reminder: your responses will be kept confidential and anonymous.

Q: People sometimes talk about using force or violence to achieve political objectives. In general, what do you think about using force or violence to advance an important political objective that you support—is it…?

1. Never justified
2. Sometimes justified
3. Usually justified
4. Always justified

Q: Your view of the use of force or violence to advance an important political objective might depend on the specific objective that was involved. What do you think about the use of force or violence in the following situations—is it never justified, sometimes justified, usually justified, or always justified?

a. To return Donald Trump to the presidency this year
b. To stop an election from being stolen
c. To stop people who do not share my beliefs from voting
d. To prevent discrimination based on race or ethnicity
e. To preserve an American way of life based on Western European traditions
f. To oppose the government when it does not share my beliefs
g. To oppose the government when it tries to take private land for public purposes

1. Never justified
2. Sometimes justified
3. Usually justified
4. Always justified

Q: You said that in general, the use of force or violence was [response inserted] to advance an important political objective that you support. Your opinion might depend on the specific objective that was involved. What do you think about the use of force or violence in the following situations—is it never justified, sometimes justified, usually justified, or always justified?

a. To stop voter fraud
b. To stop voter intimidation
c. To stop police violence
d. To reinforce the police
g. To stop a protest or demonstration
h. To support a protest or demonstration
i. To preserve the American way of life I believe in
j. To oppose Americans who do not share my beliefs

1. Never justified
2. Sometimes justified

3. Usually justified
4. Always justified

Q: Here's another group of political objectives. What do you think about the use of force or violence in the following situations—is it never justified, sometimes justified, usually justified, or always justified?

a. To protect the environment or stop climate change
b. To protect the rights of animals
c. To stop illegal immigration
d. To keep our borders open
e. To support women's reproductive rights
f. To support the right to life

1. Never justified
2. Sometimes justified
3. Usually justified
4. Always justified

(Questions asked of respondents who endorsed at least 1 use of violence to achieve a specific political objective.)

The next questions are about your personal willingness to use force or violence.

Q: In a situation where you think force or violence is justified to advance an important political objective, how willing would <u>you</u> <u>personally</u> be to use force or violence in each of these ways?

a. To damage property
b. To threaten or intimidate a person
c. To injure a person
d. To kill a person

1. Not willing
2. Somewhat willing
3. Very willing
4. Completely willing

Q: In a situation where you think force or violence is justified to advance an important political objective, how willing would <u>you</u> <u>personally</u> be to use force or violence against a person <u>because</u> they are…

a. An elected federal or state government official
b. An elected local government official
c. A public health official

d. A member of the military or National Guard
e. A police officer
f. A person who does not share your race or ethnicity
g. A person who does not share your religion
h. An election worker, such as a poll worker or vote counter
i. A person who does not share your political beliefs
j. A member of the Democratic Party
k. A member of the Republican Party

1. Not willing
2. Somewhat willing
3. Very willing
4. Completely willing

Q: You agreed that the use of force or violence could be justified to advance [one/some] of the political objectives we just discussed. In [that situation/those situations], how willing would you personally be to…

a. Use force or violence as part of a group of people who share your beliefs
b. Use force or violence on your own, as an individual
c. Organize a group of people who share your beliefs to use force or violence

1. Not willing
2. Somewhat willing
3. Very willing
4. Completely willing

Q: This is an election year. How willing would you personally be to use force or violence to make sure your preferred candidate for President takes office in each of the following situations?

a. Another candidate is declared the winner, and you believe the election is being stolen.
b. Another candidate wins legitimately.

1. Not willing
2. Somewhat willing
3. Very willing
4. Completely willing

(Question asked of all respondents.)

Q: Thinking now about the future and all the changes it might bring, how likely is it that you will use a gun in any of the following ways in the next few years—in a situation where you think force or violence is justified to advance an important political objective?

a. I will be armed with a gun.
b. I will carry a gun openly, so that people know I am armed.
c. I will threaten someone with a gun.
d. I will shoot someone with a gun.

1. Not likely
2. Somewhat likely
3. Very likely
4. Extremely likely

Party affiliation, partisanship, and political ideology

Q: Generally speaking, do you think of yourself as...Select one answer only.

1. Republican
2. Democrat
3. Independent
6. Something else

(Asked if Republican)
Q: Would you call yourself a...Select one answer only.

1. Strong Republican
2. Not very strong Republican

(Asked if Democrat)
Q: Would you call yourself a...Select one answer only.

1. Strong Democrat
2. Not very strong Democrat

(Asked if Independent or Something else)
Q: Do you think of yourself as closer to the...Select one answer only.

1. Republican Party
2. Democratic Party
3. Do not lean either way

(Asked if Republican OR Leans Republican)
Q: Do you think of yourself as a MAGA Republican?

1. No

2. Yes

(Asked if Not MAGA Republican OR Democrat / Leans Democrat)
Q: Do you think of yourself as a supporter of the MAGA movement?

1. No
2. Yes

Q: In general, do you think of yourself as...

1. Extremely liberal
2. Liberal
3. Slightly liberal
4. Moderate/middle of the road
5. Slightly conservative
6. Conservative
7. Extremely conservative

Democracy, authoritarianism, and elections

Q: When thinking about democracy in the United States these days, do you believe…?[3]

1. There is a serious threat to our democracy.
2. There may be a threat to our democracy, but it is not serious.
3. There is no threat to our democracy.

Q: Please indicate whether you believe each of the following is a threat to democracy in the United States. Is it a serious threat; a threat, but not a serious threat; or not a threat?

a. The influence of big money in elections[4]
b. The influence of foreign governments in elections
c. The possibility of political violence[4]
d. The influence of white nationalist groups
e. The influence of groups like Black Lives Matter
f. Efforts to overturn the results of elections

1. A serious threat
2. A threat, but not a serious threat
3. Not a threat

Q: How important do you think it is for the United States to remain a democracy?[5]

1. Not important

2. Somewhat important
3. Very important
4. Extremely important

Q: How much do you agree or disagree with the following statements about democracy in the United States?

a. Democracy is the best form of government.[6]
b. These days, American democracy only serves the interests of the wealthy and powerful.[1]
c. Having a strong leader for America is more important than having a democracy.
d. The 2020 election was stolen from Donald Trump, and Joe Biden is an illegitimate president.
e. We should suspend Congress for a few years so a strong leader can clean up the mess made by politicians in Washington.[7]
f. I can trust the American people as a whole when it comes to making judgments under our democratic system about the issues facing our country.[4]

1. Do not agree
2. Somewhat agree
3. Strongly agree
4. Very strongly agree

Q: Which is more important to you…?[4]

1a. Having election outcomes determined democratically
OR
1b. Having political leaders I can trust to look out for my values and interests

2a. Allowing people the personal freedom to do as they please
OR
2b. Having people work together for a better country

3a. Making sure that everyone who wants to vote can do so
OR
3b. Making sure that no one votes who is not eligible to vote

(Asked if Republican OR Leans Republican)
Q: When you think about Democrats, do you tend to think of them as…[8]

a. Political opposition – that is, if they win, you just won't get the policies you want
OR
b. Enemies – that is, if they win, your life or your entire way of life may be threatened

(Asked if Democrat OR Leans Democrat)
Q: When you think about Republicans, do you tend to think of them as…[8]

a. Political opposition - that is, if they win, you just won't get the policies you want
OR
b. Enemies - that is, if they win, your life or your entire way of life may be threatened

(Asked in 2022)
Q: People have many different views about American society. How much do you agree or disagree with each of the following?

h. Armed citizens should patrol polling places at election time.

1. Do not agree
2. Somewhat agree
3. Strongly agree
4. Very strongly agree

(Asked in 2023) Hatred, fear, and enmity toward others

Homonegativity

Q: How much do you agree or disagree with each of the following statements about gay men and lesbian women in the United States today?[9]

a. Celebrations such as "Gay Pride Day" are ridiculous, because they assume that an individual's sexual orientation should constitute a source of pride.
b. Gay men and lesbian women should stop shoving their lifestyle down other people's throats.
c. Many gay men and lesbian women use their sexual orientation so that they can obtain special rights and privileges.
d. Gay men and lesbian women who are "out of the closet" should be admired for their courage.
e. In today's tough economic times, Americans' tax dollars shouldn't be used to support gay and lesbian organizations.
f. Gay men and lesbian women should stop complaining about the way they are treated in society, and simply get on with their lives.

1. Do not agree
2. Somewhat agree
3. Strongly agree
4. Very strongly agree

Racism

Q: How much do you agree or disagree with each of the following statements about people in the United States today?

a. White people benefit from advantages in society that Black people do not have.[10] (Reverse coded.)
b. Discrimination against whites is as big a problem as discrimination against Blacks and other minorities.[11]
c. A group of people in this country is trying to replace native-born Americans with immigrants and people of color who share their political views.
d. Having more Black Americans, Latinos, and Asian Americans is good for the country.[12] (Reverse coded.)

1. Do not agree
2. Somewhat agree
3. Strongly agree
4. Very strongly agree

Transphobia

Q: How much do you agree or disagree with each of the following statements about gender in the United States today?[13]

a. I think there is something wrong with a person who says that they are neither a man nor a woman.
b. I would be upset, if someone I'd known a long time revealed to me that they used to be another gender.
c. I avoid people on the street whose gender is unclear to me.
d. When I meet someone, it is important for me to be able to identify them as a man or a woman.
e. I believe that the male/female dichotomy is natural.
f. I believe that a person can never change their gender.

1. Do not agree
2. Somewhat agree
3. Strongly agree
4. Very strongly agree

Xenophobia

Q: How much do you agree or disagree with each of the following statements about people who have immigrated to the United States?[14]

a. Interacting with immigrants makes me uneasy.
b. Immigrants cause an increase in crime.
c. I enjoy interacting with immigrants.
d. I am afraid that our own culture will be lost with an increase in immigration.
e. I am afraid that in case of political tension, immigrants will be loyal to their country of origin.

1. Do not agree
2. Somewhat agree
3. Strongly agree
4. Very strongly agree

Hostile Sexism

Q: How much do you agree or disagree with each of the following statements about women in the United States today?[15]

a. Women seek to gain power by getting control over men.
b. Women exaggerate problems they have at work.
c. Once a woman gets a man to commit to her, she usually tries to put him on a tight leash.
d. When women lose to men in a fair competition, they typically complain about being discriminated against.
e. Many women get a kick out of teasing men by seeming sexually available and then refusing male advances.
f. Feminists are making unreasonable demands of men.

1. Do not agree
2. Somewhat agree
3. Strongly agree
4. Very strongly agree

Islamophobia

Q: How much do you agree or disagree with each of the following statements about people's religious beliefs in the United States today?[16]

a. Most Muslims living in the United States are more prone to violence than other people.
b. Most Muslims living in the United States discriminate against women.
c. Most Muslims living in the United States are hostile to the United States.
d. Most Muslims living in the United States are less civilized than other people.

1. Do not agree
2. Somewhat agree
3. Strongly agree
4. Very strongly agree

Antisemitism

Q: How much do you agree or disagree with each of the following statements about people's religious beliefs in the United States today?[17]

a. Jewish people can be trusted just as much as other Americans in business.
b. Jewish people are just as loyal to the United States as other Americans.
c. Compared to other groups, Jewish people have too much power in the media.
d. Jewish people talk about the Holocaust just to further their political agenda.
e. Jewish people chase money more than other people do.

1. Do not agree
2. Somewhat agree
3. Strongly agree
4. Very strongly agree

QAnon and Christian nationalism

Q: People have many different views about society in the United States. How much do you agree or disagree with each of the following?

a. The government, media, and financial worlds in the U.S. are controlled by a group of Satan-worshipping pedophiles who run a global child sex trafficking operation.[2]
b. There is a storm coming soon that will sweep away the elites in power and restore the rightful leaders.[2]
c. The chaos in America today is evidence that we are living in what the Bible calls "the end times."[18]

1. Do not agree
2. Somewhat agree
3. Strongly agree
4. Very strongly agree

Q: How much do you agree or disagree with each of the following statements about people's religious beliefs in the United States today?[19]

a. The U.S. government should declare America a Christian nation.
b. U.S. laws should be based on Christian values.
c. If the U.S. moves away from our Christian foundations, we will not have a country anymore.
d. Being Christian is an important part of being truly American.
e. God has called Christians to exercise dominion over all areas of American society.

1. Do not agree

2. Somewhat agree
3. Strongly agree
4. Very strongly agree

(Asked in 2023) Conspiracism

Q: There is often debate about whether the public is told the whole truth about various important issues. How much do you agree or disagree with the following statements?[20]

a. The government is involved in the murder of innocent citizens and/or well-known public figures and keeps this a secret.
b. The spread of certain viruses and/or diseases is the result of the deliberate, concealed efforts of some organization.
c. The government permits or perpetrates acts of terrorism on its own soil, disguising its involvement.
d. Technology with mind-control capacities is used on people without their knowledge.
e. The government falsely blames innocent people to hide its involvement in criminal activity.
f. Experiments involving new drugs or technologies are routinely carried out on the public without their knowledge or consent.

1. Do not agree
2. Somewhat agree
3. Strongly agree
4. Very strongly agree

(Asked in 2023) Trait aggression

Q: Sometimes disagreements and conflicts occur in our lives. The next items are about ways you might respond to some of those things. How much do you agree or disagree with each of the following statements?[21]

a. Given enough provocation, I may hit another person.
b. There are people who pushed me so far that we came to blows.
c. I have threatened people I know.
d. At times I feel I have gotten a raw deal out of life.
e. Other people always seem to get the breaks.
f. I wonder why sometimes I feel so bitter about things.

1. Do not agree
2. Somewhat agree
3. Strongly agree
4. Very strongly agree

(Asked in 2022) Violence in specific nonpolitical circumstances

Now we have a few questions about the use of force or violence. "Force or violence" means physical force strong enough that it could cause pain or injury to a person. A reminder: your responses will be kept confidential and anonymous.

Q: In general, what do you think about the use of force or violence in the following situations—is it never justified, sometimes justified, usually justified, or always justified? "Force or violence" means physical force strong enough that it could cause pain or injury to a person.

a. In self defense
b. To prevent someone from injuring or killing another person
c. To prevent someone from injuring or killing themselves
d. To prevent harm or damage to property
e. To win an argument
f. In response to an insult
g. To get respect

1. Never justified
2. Sometimes justified
3. Usually justified
4. Always justified

Intimate partner violence

Most people agree that getting physical (hitting, slapping, etc.) with a partner is not a good idea, but sometimes this happens. How much do you agree or disagree with the following reasons why it might be understandable that someone could hit a partner?[22]

a. A partner hurts your child, either physically or emotionally.
b. A partner hits you first.
c. A partner cheats on you.
d. A partner steals from you.
e. A partner is drunk or using drugs.
f. A partner embarrassed or belittled you in front of others.
g. A partner continually nags you.
h. A partner threatened to hit you.
i. A partner tried to keep you from doing something.
j. A partner forces you to have sex with him or her.

1. Do not agree
2. Somewhat agree
3. Strongly agree

4. Very strongly agree

Firearm ownership and purchase

Q: Do you happen to keep any guns in your home or garage?

1. Yes
2. No

(Asked if the response to the prior question was "yes.")
Q: Do any of these guns personally belong to you?

1. Yes
2. No

Q: Have you gotten a gun since you completed our survey in [IF XPAST=1, INSERT: May or June of 2022; IF XPAST=2, INSERT: May or June of 2023]?

1. Yes
2. No

(Asked in 2022)
Q: Did you buy any guns this year, in 2022?

1. Yes
2. No

Q: Did you buy any guns in 2021?

1. Yes
2. No

Q: Did you buy any guns in 2020?

1. Yes
2. No

ADDITIONAL METHODS TEXT

Measures

The text of all questionnaire items included in this analysis is in this Supplement.

Data for party affiliation, MAGA status, and political violence measures were collected in 2024. Data for other characteristics were also collected in 2024 except as follows: 7 forms of hatred, fear, and enmity toward others, 2023; conspiracism and trait aggression, 2023; nonpolitical violence, 2022; armed citizens patrolling voting places, 2022. Data for the covariates military history and arrest history were collected in 2022.

Survey items that supplied data for the 7 forms of hatred, fear, and enmity toward others were obtained from validated scales in all but 1 case.[9,13-17] These scales were selected based on recency of development, extent of validation, and applicability to estimating associations between the phobias they measured and political violence. Questionnaire length limitations precluded the use of complete scales; we selected items for inclusion based on their direct relevance to violence. Each of the abbreviated scales had a Cronbach's α (a measure of internal consistency among items in a scale) of at least 0.754 when applied to 2023 data from this survey.[23] An abbreviated validated scale for racism[24] was included in the questionnaire, but the selected items did not have an acceptable Cronbach's α.[25] Analyses for racism relied instead on 4 items regarding racism that had a Cronbach's α of 0.81 in our 2022 survey.[26]

Questions on Christian nationalism, conspiracism, trait aggression, and intimate partner violence were also obtained from validated scales.[19-22]

Respondents were categorized as personal firearm owners, non-owners with firearms at home, and non-owners without firearms at home.

Information on political violence measures is given in the main article text.

Statistical analysis

Our approach to calculating scores for 7 forms of fear, hatred, and enmity toward others has been reported previously.[23] Approximately 4.3% of respondents were missing more than half of the items for at least 1 individual phobia, and approximately 7.1% of respondents were missing at least 1 item. These missing values were imputed using Multiple Imputation by Chained Equations (MICE). Individual item responses for the 7 phobias were then coded ordinally (e.g., do not agree = 0, somewhat agree = 1, strongly agree = 2, very strongly agree = 3) and summed for each respondent for each phobia. Summed scores were normalized to a range from 0 to 1, with 0 and 1 representing the minimum and maximum theoretically possible scores. Normalized scores were then categorized according to their position on that range (e.g., strong agreement, normalized score > 0.66… and ≤ 1; moderate agreement, normalized score > 0.33… and ≤ 0.66; weak agreement, normalized score > 0 and ≤ 0.33…; non-agreement, normalized score = 0).

In prior analyses,[23,26,27] we examined the first 3 models below and selected Model 3 as the final model, based on concordance with theory and findings from prior research. Additional testing here found that military history, number of non-traffic arrests, and total number of drinks per week were associated with MAGA status and with violence risk. They were added to create Model 4 below. Model 4 was selected for this analysis. Findings from Model 4 appear in the 'Adjusted prevalence difference" rows in tables. Model 4 was also used to produce q-values.

Model 0: unadjusted;

Model 1: adjusted for age (numerical), race and ethnicity (White, Non-Hispanic; Black, Non-Hispanic; Other, Non-Hispanic; Hispanic; 2+ Races, Non-Hispanic), and gender (Male, Female);

Model 2: additionally adjusted for income (Less than $10,000, $10,000 to $24,999, $25,000 to $49,999, $50,000 to $74,999, $75,000 to $99,999, $100,000 to $149,999, $150,000 or more), education (No high school diploma or GED, High school graduate (high school diploma or the equivalent GED), Some college or Associate's degree, Bachelor's degree, Master’s degree or higher), and Census division (New England, Mid-Atlantic, East-North Central, West-North Central, South Atlantic, East-South Central, West-South Central, Mountain, Pacific);

Model 3: additionally adjusted for rurality (Urban, Rural; derived from Rural-Urban Commuting Codes matched to census tracts (https://www.ers.usda.gov/data-products/rural-urban-commuting-area-codes/).

Model 4: additionally adjusted for military history, number of non-traffic arrests, and total number of drinks per week.

ADDITIONAL RESULTS TEXT

Item nonresponse

Three items included had nonresponse percentages above 3.0%:

Q. Which is more important to you…?

1a. Having election outcomes determined democratically

OR

1b. Having political leaders I can trust to look out for my values and interests

Nonresponse n = 302, 3.4%

2a. Allowing people the personal freedom to do as they please

OR

2b. Having people work together for a better country

Nonresponse n = 279, 3.1%

3a. Making sure that everyone who wants to vote can do so

OR

3b. Making sure that no one votes who is not eligible to vote

Nonresponse n = 301, 3.4%

Findings for non-Republican MAGA supporters

All respondents who did not identify as MAGA Republicans, regardless of party affiliation, were asked if they identified as "a supporter of the MAGA movement," and 222 respondents (2.7%, 95% CI 2.2%, 3.2%) identified as non-Republican supporters of the MAGA movement (Table S1). Just over half (53.6%) of this group identified as Independent and 33.4% as Democrat; 34.7% identified as conservative and 20.2% as liberal (Table S3). The demographics of this small group were quite different from those of MAGA Republicans. Most

were young adults (54.1% were ages 18-44), female (52.8%), and white, non-Hispanic (56.9%); comparable percentages for MAGA Republicans were 26.6%, 45.3%, and 81.7%, respectively.

When comparing this group with MAGA Republicans, we inferred that differences were statistically significant when 95% CIs for their weighted prevalence estimates did not overlap.

Non-Republican MAGA supporters were more likely than MAGA Republicans to agree strongly or very strongly that civil war was coming and that it was needed (Table 1). They tended to be more supportive of political violence, but many differences were not statistically significant. For example, non-Republican MAGA supporters were more likely to endorse violence to effect social change in 2 of 3 cases (Table 1) and political violence “in general” and for 18 of 21 specific objectives (Table 2, Table S4). Only 9 of those 18 differences were by more than 5 percentage points, however, and only the differences on violence “to prevent discrimination based on race or ethnicity” and “to support women’s reproductive rights” were statistically significant. They were more willing to damage property, threaten a person, injure a person, and kill a person (Table 3), and the first 3 differences were statistically significant. They were more willing to commit violence against all 9 target populations (Table S5), but only 3 of those differences (for violence against elected federal or state officials, election workers, and members of the military or National Guard) were statistically significant. They were more frequently willing to commit violence “to make sure [their] preferred candidate for President takes office,” whether they believed the election was being stolen or another candidate won legitimately (Table S7); these differences were also not statistically significant. They more frequently expected that they would carry a gun openly, threaten someone with a gun, and

shoot someone in a future situation in which they considered political violence justified (Table 4); again, these differences were not statistically significant.

REFERENCES

1. Survey Center on American Life. January 2021 American Perspectives Survey topline questionnaire. https://www.americansurveycenter.org/wp-content/uploads/2021/03/January-2021-APS-Topline-Questionnaire.pdf.
2. Public Religion Research Institute. The persistence of Q-Anon in the post-Trump era: an analysis of who believes the conspiracies. 2022 Feb 24. https://www.prri.org/research/the-persistence-of-qanon-in-the-post-trump-era-an-analysis-of-who-believes-the-conspiracies/.
3. NPR/PBS NewsHour/Marist National Poll. Trust in elections, threat to democracy, November 2021. 2021 November 1. https://maristpoll.marist.edu/polls/npr-pbs-newshour-marist-national-poll-trust-in-elections-threat-to-democracy-biden-approval-november-2021/.
4. Hart Research Associates. The New Republic Democracy Survey (Study #14230). 2022 March. https://newrepublic.com/article/166027/democracy-poll.
5. Grinnell College National Poll. 52% of Americans believe democracy facing "major threat." Study #2243. 2021 October 20. https://www.grinnell.edu/news/52-americans-believe-democracy-facing-major-threat.
6. The Economist/YouGov Poll. 2021 June 13-16. https://docs.cdn.yougov.com/uagnfc262c/econTabReport.pdf.
7. Democracy Fund Voter Study Group. Guide to views of The Electorate Research Survey. 2021 December. https://www.voterstudygroup.org/data/voter-survey.
8. De Pinto J, Backus F, Salvanto A. CBS News Battleground Tracker: What do voters in each party want in a candidate? 2022 Aug 2. https://www.cbsnews.com/news/what-voters-want-in-candidate-opinion-poll-2022-08-02/.
9. Morrison MA, Morrison TG. 2011 Sexual orientation bias toward gay men and lesbian women: modern homonegative attitudes and their association with discriminatory behavioral intentions. J Appl Soc Psychol. 2011;41(11):2573-2599.
10. Pew Research Center. Deep divisions in Americans' views of nation's racial history – and how to address it. 2021 August. https://www.pewresearch.org/politics/2021/08/12/deep-divisions-in-americans-views-of-nations-racial-history-and-how-to-address-it/.
11. Cox D, Lienesch R, Jones RP. Beyond economics: fears of cultural displacement pushed the white working class to Trump | PRRI/The Atlantic Report. Public Religion Research Institute. 2019 May 17. https://www.prri.org/research/white-working-class-attitudes-economy-trade-immigration-election-donald-trump/.
12. Pew Research Center. Americans see advantages and challenges in country's growing racial and ethnic diversity. 2019 May. https://www.pewresearch.org/social-trends/2019/05/08/americans-see-advantages-and-challenges-in-countrys-growing-racial-and-ethnic-diversity/.
13. Nagoshi JL, Adams KA, Terrell HK, Hill ED, Brzuzy S, Nagoshi CT. Gender differences in correlates of homophobia and transphobia. Sex Roles. 2008;59:521-531.
14. Maloni MJ, Gligor DM, Blumentritt T, Gligor N. Fear or competition? Antecedents to U.S. business student immigration attitudes. J Manag Educ. 2022;46(4):715-750.

15. Glick P, Fiske ST. Hostile and benevolent sexism: measuring ambivalent sexist attitudes toward women. Psychol Women Q. 1997;21:119-135.
16. Institute for Social Policy and Understanding. The National American Islamophobia Index. Undated. https://www.ispu.org/islamophobia-index/.
17. Allington D, Hirsch D, Katz L. The generalised antisemitism (geas) scale: validity and factor structure. J Contemp Antisemitism. 2022;5(2):1-28.
18. IFYC – PRRI Survey on Religion & COVID-19 Vaccine Trust. 2021 March. https://www.prri.org/wp-content/uploads/2021/05/Topline-IFYC-PRRI-Survey-on-Religion-and-COVID-19-Vaccine-Trust-v2_final.pdf.
19. Public Religion Research Institute and The Brookings Institution. A Christian nation? Understanding the threat of Christian nationalism to American democracy and culture. 2023 Feb 8. https://www.prri.org/research/a-christian-nation-understanding-the-threat-of-christian-nationalism-to-american-democracy-and-culture/.
20. Brotherton R, French CC, Pickering AD. Measuring belief in conspiracy theories: the generic conspiracist beliefs scale. Front Psychol. 2013;4:279.
21. Diamond PM, Magaletta PR. The Short-Form Buss-Perry Aggression Questionnaire (BPAQ-SF): a validation study with federal offenders. Assessment. 2006;13(3):227-240.
22. Copp JE, Giordano PC, Longmore MA, Manning WD. The development of attitudes toward intimate partner violence: an examination of key correlates among a sample of young adults. J Interpers Violence. 2019;34(7):1357-1387.
23. Wintemute GJ, Velasquez B, Tomsich EA, Reeping PM, Robinson SL, Tancredi D, Pear VA. Fear, loathing, and political violence in the United States: findings from a nationally representative survey. [Preprint.] SocArXiv. 2024. Online publication May 24. DOI: 10.31235/osf.io/vjdk6.
24. Uhlmann EL, Brescoll VL, Machery E. The motives underlying stereotype-based discrimination against members of stigmatized groups. Soc Justice Res 2010;23(1): 1-16.
25. Cronbach LJ. Coefficient alpha and the internal structure of tests. Psychometrika 1951;16(3):297-334.
26. Wintemute GJ, Velasquez B, Li Y, Tomsich EA, Reeping PM, Robinson SL. Racist and pro-violence beliefs, approval of extreme right-wing political organizations and movements, and support for political violence in the United States. [Preprint.] SocArXiv. 2023. Online publication Dec 4. DOI: 10.31235/osf.io/c9vtr.
27. Wintemute GJ, Li Y, Velasquez B, Crawford A, Reeping PM, Tomsich EA. Expectations of and perceived need for civil war in the USA: findings from a 2023 nationally representative survey. Inj Epidemiol. 2024;11(1):40.

Figure S1. Flowchart of survey assignment and completion counts for Waves 1-3 in 2022-2024

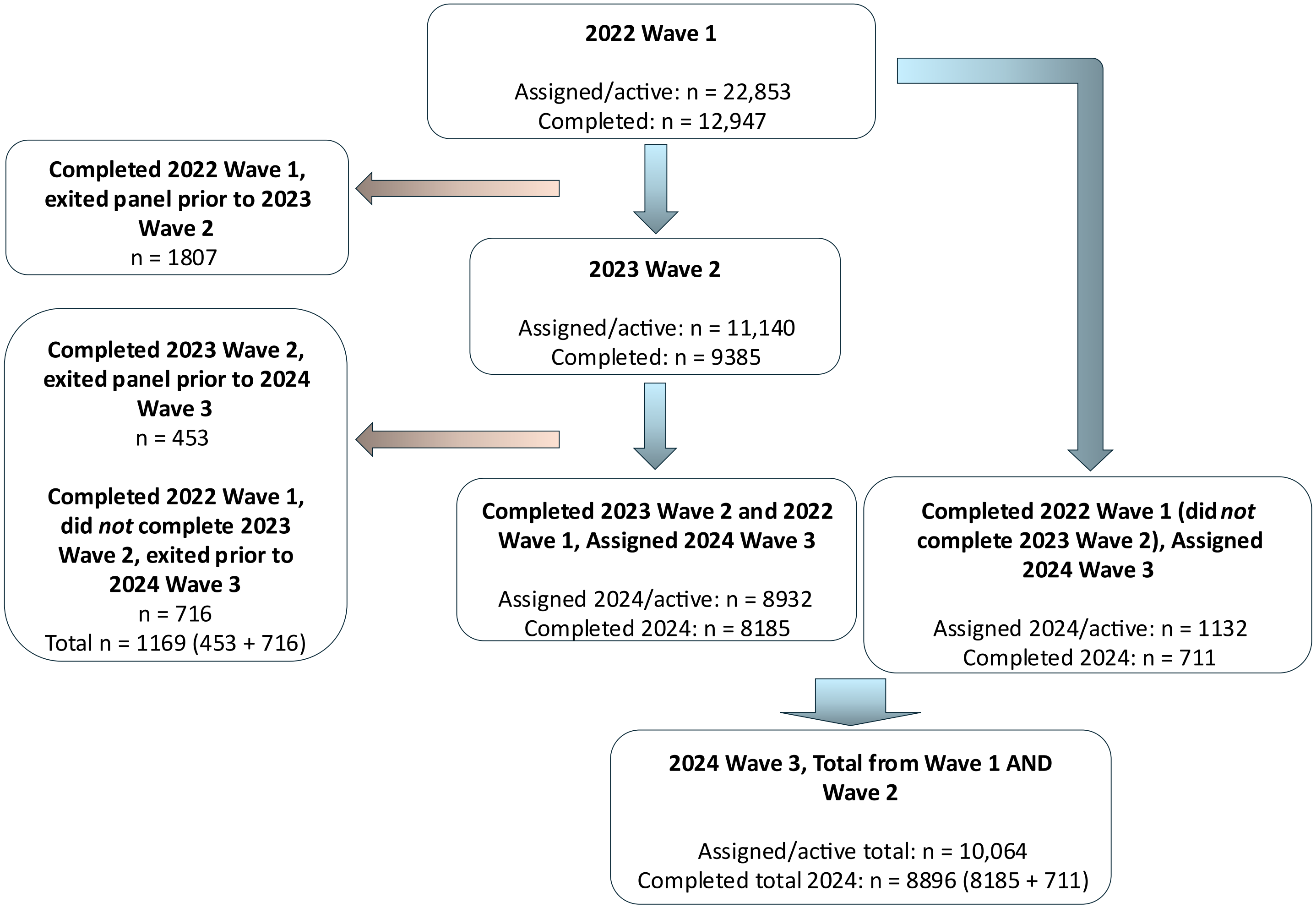

This figure previously appeared as Figure S1 in Wintemute GJ, Crawford A, Tomsich EA, Pear VA. Trends in Views of Democracy and Society and support for political violence in the USA, 2022-2024: findings from a nationally representative survey. Inj Epidemiol 2025;12(1):4.

Table S1. Sociodemographic characteristics (weighted) of Wave 3 respondents

Characteristic	2024 Respondents* (n=8896)	
	**Unweighted n**	**Weighted % (95% CI)**
Age		
18-24	176	6.6 (5.7, 7.7)
25-34	753	17.4 (16.1, 18.7)
35-44	1094	16.9 (15.9, 18.1)
45-54	1150	14.9 (13.9, 15.9)
55-64	1827	18.4 (17.4, 19.4)
65-74	2249	15.1 (14.3, 15.8)
75+	1647	10.8 (10.1, 11.5)
Non-response	0	0.0 (0.0, 0.0)
Gender		
Female	3667	50.9 (49.5, 52.3)
Male	5055	47.5 (46.1, 48.9)
Transgender	46	0.5 (0.3, 0.8)
Non-binary	58	0.8 (0.6, 1.1)
Other	20	0.3 (0.2, 0.5)
Non-response	0	0.0 (0.0, 0.0)
Race/Ethnicity		
White, Non-Hispanic	6663	62.7 (61.2, 64.2)
Black, Non-Hispanic	720	12.0 (11.0, 13.1)
Hispanic, any race	940	16.9 (15.7, 18.2)
American Indian or Alaska Native, Non-Hispanic	44	1.1 (0.7, 1.6)
Asian American or Pacific Islander, non-Hispanic	261	5.4 (4.7, 6.3)
Some other race, Non-Hispanic	18	0.1 (0.1, 0.2)
2+ Races, Non-Hispanic	250	1.8 (1.4, 2.2)
Non-response	0	0.0 (0.0, 0.0)
Marital status		
Now married	5655	57.0 (55.6, 58.5)
Widowed	630	4.7 (4.2, 5.2)
Divorced	979	8.5 (7.9, 9.2)
Separated	127	1.8 (1.4, 2.3)
Never married	1505	27.9 (26.5, 29.4)
Non-response	0	0.0 (0.0, 0.0)

Table S1, continued.

Characteristic	2024 Respondents* (n=8896)	
	Unweighted n	Weighted % (95% CI)
Education		
No high school diploma or GED	330	7.4 (6.5, 8.4)
High school graduate	1784	26.9 (25.5, 28.2)
Some college or Associate's degree	2691	28.7 (27.5, 30.0)
Bachelor's degree	2257	21.0 (19.9, 22.1)
Master's degree or higher	1834	16.0 (15.2, 17.0)
Non-response	0	0.0 (0.0, 0.0)
Household Income		
Less than $10,000	265	4.9 (4.2, 5.7)
$10,000 to $24,999	609	7.9 (7.1, 8.7)
$25,000 to $49,999	1446	17.1 (16.0, 18.3)
$50,000 to $74,999	1424	15.3 (14.3, 16.3)
$75,000 to $99,999	1367	13.3 (12.4, 14.3)
$100,000 to $149,999	1799	18.5 (17.5, 19.6)
$150,000 or more	1986	23.0 (21.9, 24.2)
Non-response	0	0.0 (0.0, 0.0)
Employment		
Working - as a paid employee	4134	53.2 (51.8, 54.7)
Working - self-employed	669	7.2 (6.6, 8.0)
Not working - on temporary layoff	37	0.6 (0.4, 0.9)
Not working - looking for work	251	4.9 (4.2, 5.7)
Not working - retired	3154	21.4 (20.5, 22.4)
Not working - disabled	258	4.4 (3.8, 5.1)
Not working - other	393	8.2 (7.3, 9.1)
Non-response	0	0.0 (0.0, 0.0)
Census division		
New England	362	4.6 (4.1, 5.3)
Mid-Atlantic	960	12.4 (11.5, 13.4)
East-North Central	1306	14.3 (13.4, 15.3)
West-North Central	647	6.4 (5.8, 7.1)
South Atlantic	1754	20.6 (19.4, 21.8)
East-South Central	514	5.9 (5.3, 6.6)
West-South Central	902	11.7 (10.8, 12.7)
Mountain	796	7.7 (7.0, 8.5)
Pacific	1655	16.3 (15.3, 17.4)
Non-response	0	0.0 (0.0, 0.0)

* Most values are as of 2022; census division values were updated for 2024, but other demographics were not.

This table previously appeared as part of Table 1 in Wintemute GJ, Crawford A, Tomsich EA, Pear VA. Trends in Views of Democracy and Society and support for political violence in the USA, 2022-2024: findings from a nationally representative survey. Inj Epidemiol 2025;12(1):4.

Table S2. Sociodemographic characteristics (unweighted) of respondents and non-respondents

Characteristic	Respondents* (n=8896)		Non-respondents (n = 1086)	
	Unweighted n	Unweighted %	Unweighted n	Unweighted %
**Age**				
18-24	176	2.0	58	5.3
25-34	753	8.5	129	11.9
35-44	1094	12.3	178	16.4
45-54	1150	12.9	154	14.2
55-64	1827	20.5	220	20.3
65-74	2249	25.3	223	20.5
75+	1647	18.5	124	11.4
Non-response	0	0.0	0	0.0
**Gender**				
Male	5147	57.9	529	48.7
Female	3749	42.1	557	51.3
Non-response	0	0.0	0	0.0
**Race/Ethnicity**				
Black, non-Hispanic	702	7.9	116	10.7
Hispanic, any race	959	10.8	162	14.9
White, non-Hispanic	6673	75.0	737	67.9
Other, non-Hispanic	316	3.6	44	4.1
2+races, non-Hispanic	246	2.8	27	2.5
Non-response	0	0.0	0	0.0
**Marital status**				
Now married	5655	63.6	623	57.4
Widowed	630	7.1	81	7.5
Divorced	979	11.0	108	9.9
Separated	127	1.4	17	1.6
Never married	1505	16.9	257	23.7
Non-response	0	0.0	0	0.0

Table S2, continued.

Characteristic	Respondents* (n=8896)		Non-respondents (n = 1086)	
	Unweighted n	Unweighted %	Unweighted n	Unweighted %
**Education**				
No high school diploma or GED	330	3.7	65	6.0
High school graduate (diploma, G	1784	20.1	231	21.3
Some college or Associate's degr	2691	30.2	331	30.5
Bachelor's degree	2257	25.4	266	24.5
Master's degree or higher	1834	20.6	193	17.8
Non-response	0	0.0	0	0.0
**Household Income**				
Less than $10,000	265	3	37	3.4
$10,000 to $24,999	609	6.8	126	11.6
$25,000 to $49,999	1446	16.3	203	18.7
$50,000 to $74,999	1424	16	200	18.4
$75,000 to $99,999	1367	15.4	151	13.9
$100,000 to $149,999	1799	20.2	193	17.8
$150,000 or more	1986	22.3	176	16.2
Non-response	0	0.0	0	0
**Employment**				
Working full-time	3593	40.4	484	44.6
Working part-time	1038	11.7	150	13.8
Not working	4265	47.9	452	41.6
Non-response	0	0.0	0	0.0
**Census division**				
New England	362	4.1	35	3.2
Mid-Atlantic	960	10.8	134	12.3
East-North Central	1306	14.7	171	15.7
West-North Central	647	7.3	71	6.5
South Atlantic	1754	19.7	221	20.3
East-South Central	514	5.8	58	5.3
West-South Central	902	10.1	112	10.3
Mountain	796	8.9	78	7.2
Pacific	1655	18.6	206	19.0
Non-response	0	0.0	0	0.0

* Most values are as of 2022; census division values were updated for 2024, but other demographics were not.

This table previously appeared as part of Table S1 in Wintemute GJ, Crawford A, Tomsich EA, Pear VA. Trends in Views of Democracy and Society and support for political violence in the USA, 2022-2024: findings from a nationally representative survey. Inj Epidemiol 2025;12(1):4.

Table S3. Sociodemographic characteristics (weighted) of respondents by MAGA affiliation

Characteristic	Non-MAGA, Non-Republican		MAGA Republican		MAGA Supporter, Republican	
	Unweighted n	Weighted % (95% CI)	Unweighted n	Weighted % (95% CI)	Unweighted n	Weighted % (95% CI)
Age						
18-24	111	7.4 (6.1,8.8)	15	3.9 (1.8,5.9)	11	8.5 (3.6,13.3)
25-34	477	19.2 (17.4,21.0)	62	11.7 (8.6,14.9)	35	10.6 (6.7,14.4)
35-44	647	18.1 (16.6,19.6)	99	11.0 (8.5,13.6)	62	12.4 (9.0,15.8)
45-54	605	14.5 (13.2,15.8)	166	15.0 (12.5,17.5)	82	15.4 (11.3,19.5)
55-64	905	17.1 (15.8,18.4)	294	20.6 (18.0,23.2)	138	19.9 (16.3,23.6)
65-74	1191	14.5 (13.5,15.5)	372	19.3 (17.0,21.6)	185	19.6 (16.2,22.9)
75+	795	9.2 (8.4,10.0)	338	18.4 (15.8,21.0)	133	13.7 (10.6,16.7)
Gender						
Female	2174	53.4 (51.5,55.3)	485	45.3 (41.7,49.0)	181	37.9 (32.6,43.2)
Male	2557	46.6 (44.7,48.5)	861	54.7 (51.0,58.3)	465	62.1 (56.8,67.4)
Race and Ethnicity						
White, Non-Hispanic	3153	53.8 (51.9,55.8)	1183	81.7 (78.2,85.2)	570	83.8 (79.1,88.6)
Hispanic	618	20.6 (18.9,22.4)	90	10.3 (7.6,13.0)	31	5.3 (3.1,7.4)
Black, Non-Hispanic	609	16.7 (15.2,18.3)	12	1.4 (0.4,2.5)	7	1.4 (0.3,2.6)
Other, Non-Hispanic	214	7.3 (6.1,8.4)	29	4.7 (2.3,7.0)	14	5.4 (1.9,8.9)
2+ Races, Non-Hispanic	137	1.6 (1.2,1.9)	32	1.9 (1.0,2.9)	24	4.1 (1.1,7.1)
Marital Status						
Now married	2808	52.4 (50.5,54.4)	895	62.5 (58.9,66.1)	477	66.8 (61.2,72.3)
Never married	1010	33.4 (31.4,35.4)	141	17.5 (14.2,20.8)	66	19.6 (14.1,25.1)
Divorced or Separated	584	9.8 (8.8,10.9)	195	13.3 (11.0,15.5)	74	10.5 (7.7,13.3)
Widowed	329	4.3 (3.7,4.9)	115	6.8 (5.0,8.5)	29	3.2 (1.6,4.7)
Education						
No high school diploma or GED	191	8.0 (6.7,9.3)	42	5.6 (3.6,7.7)	11	3.4 (1.0,5.8)
High school graduate	850	25.1 (23.3,26.9)	350	35.6 (32.0,39.3)	117	25.0 (19.7,30.4)
Some college or Associate's degree	1324	27.3 (25.6,29.0)	505	35.1 (31.7,38.4)	196	32.9 (27.9,37.8)
Bachelor's degree	1198	21.1 (19.6,22.5)	289	15.4 (13.0,17.7)	191	23.0 (19.1,27.0)
Master's degree or higher	1168	18.5 (17.3,19.8)	160	8.3 (6.7,10.0)	131	15.6 (12.3,18.9)

Table S3, continued.

Characteristic	Non-MAGA, Non-Republican		MAGA Republican		MAGA Supporter, Republican	
	Unweighted n	Weighted % (95% CI)	Unweighted n	Weighted % (95% CI)	Unweighted n	Weighted % (95% CI)
Income						
Less than $10,000	164	5.5 (4.5,6.5)	30	3.9 (2.1,5.6)	10	2.9 (0.8,4.9)
$10,000 to $24,999	343	7.9 (6.8,8.9)	94	9.4 (6.8,12.0)	19	3.4 (1.5,5.2)
$25,000 to $49,999	764	17.8 (16.2,19.4)	263	18.9 (16.2,21.6)	85	13.8 (9.6,18.1)
$50,000 to $74,999	760	15.8 (14.4,17.2)	237	15.9 (13.4,18.5)	108	15.2 (11.5,19.0)
$75,000 to $99,999	722	13.4 (12.1,14.6)	199	12.4 (10.3,14.5)	111	14.9 (11.5,18.3)
$100,000 to $149,999	899	16.8 (15.5,18.2)	296	20.9 (18.0,23.8)	128	19.7 (15.4,24.1)
$150,000 or more	1079	22.8 (21.3,24.3)	227	18.6 (15.7,21.5)	185	30.1 (25.3,34.9)
Employment						
Not working	2236	38.8 (37.0,40.7)	743	48.1 (44.5,51.7)	308	38.9 (33.7,44.0)
Working part-time	573	13.3 (12.0,14.7)	122	9.3 (7.1,11.6)	73	13.5 (9.6,17.5)
Working full-time	1922	47.8 (45.9,49.7)	481	42.6 (39.0,46.2)	265	47.6 (42.2,52.9)
Census Division						
East-North Central	685	14.1 (12.8,15.4)	184	12.3 (10.0,14.6)	93	13.2 (9.9,16.5)
East-South Central	235	5.0 (4.2,5.9)	97	7.4 (5.5,9.2)	49	9.8 (5.8,13.8)
Mid-Atlantic	495	12.2 (11.0,13.5)	142	11.1 (8.9,13.2)	79	15.4 (11.4,19.4)
Mountain	393	7.0 (6.1,8.0)	133	8.0 (6.2,9.9)	60	10.2 (6.5,13.9)
New England	229	5.5 (4.6,6.4)	41	3.7 (2.3,5.0)	23	3.9 (2.0,5.8)
Pacific	1062	18.9 (17.5,20.4)	170	11.2 (9.0,13.3)	91	10.3 (7.3,13.3)
South Atlantic	890	20.2 (18.7,21.8)	323	26.6 (23.2,30.0)	127	18.0 (14.0,21.9)
West-North Central	316	5.7 (4.9,6.5)	100	7.6 (5.8,9.5)	47	6.6 (4.1,9.0)
West-South Central	426	11.2 (9.9,12.6)	156	12.2 (9.8,14.6)	77	12.6 (9.4,15.8)
Ideology						
Extremely conservative	35	1.1 (0.7,1.6)	281	19.8 (17.1,22.6)	53	8.7 (5.6,11.8)
Conservative	194	4.8 (3.9,5.7)	762	51.3 (47.7,55.0)	342	45.9 (40.6,51.2)
Slightly conservative	299	5.4 (4.6,6.3)	140	11.6 (9.1,14.1)	140	22.5 (17.9,27.1)
Moderate/middle of the road	1914	41.9 (40.0,43.8)	125	13.7 (10.9,16.6)	94	18.6 (14.2,23.0)
Slightly liberal	714	14.3 (13.0,15.6)	14	1.6 (0.3,2.9)	6	2.4 (0.2,4.6)
Liberal	1137	22.9 (21.4,24.5)	8	0.8 (0.0,1.6)	3	0.4 (-0.1,1.0)
Extremely liberal	331	7.5 (6.5,8.5)	1	0.1 (-0.1,0.3)	2	0.5 (-0.2,1.2)
Skipped	62	2.1 (1.4,2.7)	6	1.0 (-0.1,2.1)	4	0.9 (-0.1,1.9)

Table S3, continued.

Characteristic	MAGA Supporter, Non-Republican		Non-MAGA, Republican		Unclassified	
	Unweighted n	Weighted % (95% CI)	Unweighted n	Weighted % (95% CI)	Unweighted n	Weighted % (95% CI)
Age						
18-24	3	3.7 (-0.6,7.9)	30	5.3 (3.3,7.2)	6	7.8 (1.7,13.9)
25-34	34	30.1 (20.7,39.6)	124	14.9 (12.1,17.7)	21	19.6 (11.8,27.4)
35-44	28	20.3 (12.5,28.1)	228	17.5 (15.0,19.9)	30	18.9 (12.1,25.7)
45-54	28	13.3 (7.6,19.1)	246	16.5 (14.2,18.7)	23	13.2 (7.5,18.9)
55-64	38	12.9 (8.0,17.7)	420	22.4 (19.9,24.9)	32	12.9 (8.0,17.8)
65-74	50	10.7 (6.4,14.9)	380	13.4 (11.7,15.0)	71	15.6 (11.2,20.0)
75+	41	9.0 (5.2,12.9)	294	10.2 (8.6,11.8)	46	12.0 (7.4,16.6)
Gender						
Female	88	52.8 (43.7,62.0)	687	51.0 (47.9,54.2)	134	63.1 (55.0,71.2)
Male	134	47.2 (38.0,56.3)	1035	49.0 (45.8,52.1)	95	36.9 (28.8,45.0)
Race and Ethnicity						
White, Non-Hispanic	152	56.9 (47.5,66.4)	1461	77.5 (74.5,80.6)	154	53.4 (44.8,62.0)
Hispanic	33	16.6 (9.9,23.2)	151	12.8 (10.4,15.3)	36	21.6 (14.2,28.9)
Black, Non-Hispanic	17	18.7 (9.5,27.9)	33	2.7 (1.5,3.8)	24	14.1 (8.0,20.1)
Other, Non-Hispanic	7	5.1 (0.7,9.4)	42	5.6 (3.6,7.6)	10	9.3 (3.1,15.5)
2+ Races, Non-Hispanic	13	2.7 (0.8,4.7)	35	1.4 (0.7,2.0)	5	1.7 (-0.5,3.8)
Marital Status						
Now married	125	43.0 (34.2,51.8)	1217	69.0 (65.9,72.0)	133	50.3 (41.8,58.8)
Never married	44	33.6 (24.1,43.1)	199	17.3 (14.5,20.1)	45	33.6 (24.9,42.3)
Divorced or Separated	42	19.0 (11.7,26.3)	188	9.4 (7.8,11.1)	23	7.1 (3.7,10.6)
Widowed	11	4.3 (1.0,7.7)	118	4.3 (3.2,5.4)	28	9.0 (5.0,13.0)
Education						
No high school diploma or GED	16	14.0 (6.3,21.7)	49	5.7 (3.8,7.6)	21	12.8 (6.8,18.9)
High school graduate	48	27.4 (18.9,35.8)	334	24.2 (21.3,27.0)	85	42.6 (34.2,51.1)
Some college or Associate's degree	79	30.7 (22.3,39.0)	518	28.9 (26.0,31.7)	69	22.7 (16.3,29.2)
Bachelor's degree	52	20.1 (13.3,26.8)	495	25.8 (23.1,28.4)	32	11.5 (5.9,17.2)
Master's degree or higher	27	7.9 (4.2,11.5)	326	15.5 (13.3,17.6)	22	10.2 (5.2,15.3)

Table S3, continued.

Characteristic	MAGA Supporter, Non-Republican		Non-MAGA, Republican		Unclassified	
	Unweighted n	Weighted % (95% CI)	Unweighted n	Weighted % (95% CI)	Unweighted n	Weighted % (95% CI)
Income						
Less than $10,000	19	14.7 (7.1,22.3)	26	2.1 (1.0,3.1)	16	8.6 (3.8,13.4)
$10,000 to $24,999	21	11.4 (5.6,17.1)	105	7.0 (5.2,8.8)	27	11.0 (5.7,16.4)
$25,000 to $49,999	34	13.1 (7.3,18.9)	235	13.9 (11.6,16.2)	65	24.0 (17.1,30.9)
$50,000 to $74,999	35	13.6 (7.5,19.6)	245	13.4 (11.2,15.5)	39	15.2 (9.1,21.3)
$75,000 to $99,999	43	11.9 (7.1,16.8)	271	13.7 (11.6,15.8)	21	12.1 (6.2,18.1)
$100,000 to $149,999	42	20.0 (12.8,27.2)	398	22.5 (20.0,25.1)	36	15.9 (9.8,22.0)
$150,000 or more	28	15.3 (8.2,22.5)	442	27.5 (24.6,30.3)	25	13.1 (7.2,19.0)
Employment						
Not working	117	46.6 (37.5,55.8)	731	34.8 (31.9,37.8)	130	45.4 (37.1,53.7)
Working part-time	28	15.9 (9.0,22.8)	213	13.3 (11.2,15.5)	29	15.0 (8.2,21.8)
Working full-time	77	37.5 (28.5,46.5)	778	51.8 (48.6,55.0)	70	39.6 (31.2,48.1)
Census Division						
East-North Central	36	13.4 (6.8,20.1)	268	16.0 (13.7,18.3)	40	18.0 (11.5,24.6)
East-South Central	14	7.9 (2.6,13.2)	105	6.5 (4.9,8.1)	14	4.8 (1.2,8.4)
Mid-Atlantic	30	16.2 (9.7,22.7)	186	12.8 (10.6,15.0)	28	10.9 (6.3,15.6)
Mountain	13	3.5 (0.6,6.4)	181	9.7 (7.8,11.6)	16	6.5 (2.4,10.6)
New England	7	3.3 (0.4,6.3)	55	3.1 (2.1,4.1)	7	4.2 (-0.1,8.5)
Pacific	42	18.6 (11.7,25.6)	241	12.1 (10.0,14.1)	49	19.1 (12.7,25.5)
South Atlantic	47	21.6 (13.7,29.6)	332	18.4 (16.0,20.8)	35	20.1 (13.2,26.9)
West-North Central	12	4.5 (0.7,8.3)	157	8.5 (6.8,10.3)	15	5.3 (1.9,8.7)
West-South Central	21	10.8 (5.4,16.2)	197	12.8 (10.6,15.1)	25	11.1 (5.4,16.8)
Ideology						
Extremely conservative	10	3.8 (0.7,6.8)	86	5.1 (3.7,6.6)	9	3.6 (0.6,6.6)
Conservative	43	14.2 (8.1,20.3)	631	32.4 (29.5,35.3)	53	16.2 (10.6,21.7)
Slightly conservative	31	16.7 (9.2,24.3)	459	25.0 (22.4,27.7)	17	5.6 (2.4,8.7)
Moderate/middle of the road	96	41.8 (32.8,50.7)	455	30.7 (27.7,33.7)	88	41.5 (33.1,50.0)
Slightly liberal	14	6.6 (2.2,11.1)	33	2.3 (1.3,3.3)	6	3.5 (0.4,6.6)
Liberal	13	8.7 (2.6,14.8)	24	2.6 (1.2,4.0)	18	8.5 (3.9,13.1)
Extremely liberal	10	4.9 (1.3,8.5)	7	1.0 (0.1,2.0)	2	2.5 (-1.6,6.5)
Skipped	3	3.4 (-0.9,7.7)	11	0.9 (0.2,1.6)	34	18.7 (11.9,25.4)

Table S4. MAGA affiliation and justification for violence to advance additional specific political objectives

What do you think about the use of force or violence in the following situations?	Non-MAGA, Non-Republican		MAGA Republican		MAGA Supporter, Republican	
	**Unweighted n**	**Weighted % (95% CI)**	**Unweighted n**	**Weighted % (95% CI)**	**Unweighted n**	**Weighted % (95% CI)**
To stop voter fraud						
Never justified	3972	83.2 (81.7,84.7)	806	60.5 (56.9,64.1)	414	66.2 (61.1,71.3)
Sometimes justified	489	10.6 (9.4,11.8)	321	23.9 (20.7,27.1)	183	27.8 (22.9,32.7)
Usually or always justified	242	5.7 (4.8,6.7)	217	15.5 (12.9,18.2)	45	5.1 (3.4,6.8)
aPD (95% CI; q-value)	Referent		9.8 (7.1,12.6; <0.001)		1.1 (-1.0,3.1; 0.51)	
To stop voter intimidation						
Never justified	3302	70.5 (68.7,72.3)	805	61.7 (58.1,65.2)	399	63.3 (58.1,68.6)
Sometimes justified	1103	22.5 (20.9,24.1)	363	25.5 (22.4,28.6)	203	29.2 (24.4,33.9)
Usually or always justified	295	6.6 (5.6,7.5)	174	12.6 (10.0,15.2)	38	6.2 (2.9,9.5)
aPD (95% CI; q-value)	Referent		6.6 (3.9,9.4; <0.001)		1.7 (-1.7,5.2; 0.65)	
To reinforce the police						
Never justified	2985	68.0 (66.2,69.7)	351	30.8 (27.3,34.2)	174	31.9 (26.8,37.0)
Sometimes justified	1371	24.7 (23.0,26.3)	599	43.1 (39.5,46.7)	330	50.4 (45.0,55.7)
Usually or always justified	343	6.9 (5.9,7.8)	390	25.6 (22.5,28.8)	138	17.5 (13.9,21.0)
aPD (95% CI; q-value)	Referent		17.4 (14.1,20.7; <0.001)		10.9 (7.1,14.6; <0.001)	
To stop police violence						
Never justified	2619	55.6 (53.7,57.5)	738	53.0 (49.4,56.7)	367	55.1 (49.7,60.5)
Sometimes justified	1673	33.6 (31.8,35.4)	456	33.7 (30.3,37.2)	234	37.3 (32.0,42.6)
Usually or always justified	407	10.3 (9.1,11.6)	149	13.0 (10.3,15.7)	41	6.6 (4.2,9.0)
aPD (95% CI; q-value)	Referent		4.3 (1.4,7.2; 0.02)		-1.2 (-4.1,1.7; 0.66)	
To stop illegal immigration						
Never justified	3613	76.9 (75.2,78.5)	380	32.6 (29.1,36.2)	195	33.1 (27.9,38.3)
Sometimes justified	807	16.4 (15.0,17.9)	498	36.5 (33.0,39.9)	254	37.3 (32.2,42.3)
Usually or always justified	280	6.1 (5.2,7.1)	464	30.4 (27.2,33.6)	193	28.6 (23.7,33.4)
aPD (95% CI; q-value)	Referent		24.4 (21.0,27.9; <0.001)		23.7 (18.7,28.8; <0.001)	
To keep our borders open						
Never justified	3815	79.6 (78.1,81.2)	1074	78.1 (74.9,81.3)	537	82.0 (77.7,86.3)
Sometimes justified	688	14.8 (13.5,16.2)	146	12.2 (9.5,14.8)	63	9.8 (6.7,12.9)
Usually or always justified	190	4.8 (3.9,5.7)	120	9.0 (6.9,11.1)	43	7.4 (4.5,10.3)
aPD (95% CI; q-value)	Referent		4.8 (2.4,7.2; 0.003)		4.3 (1.1,7.5; 0.09)	

Table S4, continued.

What do you think about the use of force or violence in the following situations?	Non-MAGA, Non-Republican		MAGA Republican		MAGA Supporter, Republican	
	Unweighted n	Weighted % (95% CI)	Unweighted n	Weighted % (95% CI)	Unweighted n	Weighted % (95% CI)
To stop a protest or demonstration						
Never justified	3551	75.4 (73.7,77.0)	655	49.5 (45.9,53.2)	313	51.3 (46.0,56.7)
Sometimes justified	1006	20.4 (18.8,21.9)	532	38.8 (35.3,42.4)	287	40.7 (35.5,45.8)
Usually or always justified	146	3.8 (3.0,4.6)	153	11.1 (8.9,13.4)	42	7.1 (4.4,9.8)
aPD (95% CI; q-value)	Referent		7.9 (5.4,10.4; <0.001)		4.8 (1.8,7.8; 0.02)	
To support a protest or demonstration						
Never justified	3953	80.5 (78.8,82.1)	1098	78.0 (74.7,81.3)	552	85.7 (82.1,89.4)
Sometimes justified	622	15.6 (14.1,17.1)	185	16.4 (13.4,19.4)	82	12.2 (9.0,15.3)
Usually or always justified	127	3.5 (2.7,4.3)	59	5.2 (3.4,7.1)	8	1.2 (-0.0,2.4)
aPD (95% CI; q-value)	Referent		2.8 (0.7,4.8; 0.15)		-1.0 (-2.6,0.6; 0.56)	
To support women's reproductive rights						
Never justified	3607	74.3 (72.5,76.0)	1106	80.6 (77.5,83.7)	572	86.2 (81.7,90.6)
Sometimes justified	717	16.5 (15.0,17.9)	145	11.3 (8.8,13.8)	45	7.5 (4.2,10.8)
Usually or always justified	376	8.7 (7.6,9.8)	88	7.3 (5.2,9.4)	25	5.4 (2.5,8.4)
aPD (95% CI; q-value)	Referent		-0.7 (-3.2,1.8; 0.77)		-1.2 (-4.5,2.1; 0.75)	
To support the right to life						
Never justified	3909	80.1 (78.5,81.7)	857	62.3 (58.7,65.9)	472	72.7 (67.7,77.7)
Sometimes justified	495	11.7 (10.4,13.0)	282	21.4 (18.3,24.5)	113	17.0 (13.0,20.9)
Usually or always justified	295	7.6 (6.5,8.7)	200	15.6 (12.8,18.4)	58	9.5 (5.8,13.1)
aPD (95% CI; q-value)	Referent		8.3 (5.4,11.3; <0.001)		4.1 (0.4,7.7; 0.09)	
To protect the environment or stop climate change						
Never justified	3691	75.6 (73.8,77.3)	1148	83.5 (80.6,86.5)	579	85.2 (80.4,89.9)
Sometimes justified	708	16.2 (14.7,17.7)	129	10.4 (8.0,12.9)	55	11.9 (7.5,16.3)
Usually or always justified	299	7.6 (6.5,8.6)	63	5.4 (3.6,7.1)	9	2.1 (0.4,3.8)
aPD (95% CI; q-value)	Referent		-0.8 (-3.1,1.4; 0.68)		-3.2 (-5.4,-1.0; 0.05)	
To protect the rights of animals						
Never justified	3377	69.9 (68.1,71.7)	873	64.8 (61.3,68.3)	460	67.9 (62.7,73.2)
Sometimes justified	960	20.3 (18.7,21.8)	295	20.2 (17.3,23.1)	131	22.6 (17.8,27.5)
Usually or always justified	359	9.1 (7.9,10.3)	172	14.4 (11.6,17.1)	51	8.3 (5.4,11.2)
aPD (95% CI; q-value)	Referent		5.6 (2.6,8.6; 0.006)		1.4 (-1.9,4.8; 0.65)	

Table S4, continued.

What do you think about the use of force or violence in the following situations?	MAGA Supporter, Non-Republican		Non-MAGA, Republican		Unclassified	
	Unweighted n	Weighted % (95% CI)	Unweighted n	Weighted % (95% CI)	Unweighted n	Weighted % (95% CI)
To stop voter fraud						
Never justified	139	60.8 (51.6,70.0)	1308	75.9 (73.2,78.7)	123	66.7 (58.1,75.4)
Sometimes justified	38	16.7 (9.8,23.7)	274	16.5 (14.0,18.9)	23	11.7 (5.3,18.1)
Usually or always justified	43	22.3 (14.2,30.5)	129	7.2 (5.6,8.8)	27	10.9 (6.3,15.5)
aPD (95% CI; q-value)	13.9 (5.4,22.4; 0.008)		2.9 (1.1,4.7; 0.01)		4.4 (-0.9,9.6; 0.23)	
To stop voter intimidation						
Never justified	139	62.0 (53.1,71.0)	1227	71.4 (68.4,74.3)	125	66.0 (57.1,74.8)
Sometimes justified	51	23.1 (15.1,31.0)	376	21.6 (18.9,24.3)	33	16.9 (9.8,24.1)
Usually or always justified	30	14.8 (8.6,20.9)	105	6.4 (4.8,8.1)	16	6.7 (3.1,10.3)
aPD (95% CI; q-value)	5.3 (-1.0,11.7; 0.37)		1.7 (-0.4,3.7; 0.37)		-0.5 (-4.6,3.7; 0.92)	
To reinforce the police						
Never justified	88	52.2 (43.0,61.3)	668	45.3 (42.1,48.5)	80	45.3 (36.1,54.5)
Sometimes justified	86	32.7 (24.5,40.9)	786	40.7 (37.6,43.8)	66	30.8 (22.6,39.1)
Usually or always justified	46	15.0 (9.2,20.8)	254	13.4 (11.2,15.5)	29	14.0 (7.6,20.3)
aPD (95% CI; q-value)	5.3 (-0.2,10.8; 0.13)		7.3 (4.9,9.7; <0.001)		6.7 (-0.1,13.4; 0.13)	
To stop police violence						
Never justified	102	47.2 (38.0,56.5)	1013	58.7 (55.6,61.9)	95	46.1 (36.9,55.2)
Sometimes justified	84	36.6 (27.7,45.5)	561	33.1 (30.0,36.1)	56	33.3 (24.2,42.3)
Usually or always justified	33	15.9 (9.6,22.2)	133	7.6 (6.0,9.2)	23	10.3 (5.5,15.1)
aPD (95% CI; q-value)	1.8 (-4.7,8.3; 0.77)		-0.5 (-2.5,1.6; 0.80)		0.3 (-5.3,6.0; 0.96)	
To stop illegal immigration						
Never justified	86	44.6 (35.4,53.9)	835	52.6 (49.4,55.8)	95	52.7 (43.5,62.0)
Sometimes justified	62	24.1 (16.3,31.8)	579	29.9 (27.1,32.7)	49	25.6 (17.6,33.6)
Usually or always justified	72	31.2 (22.8,39.5)	296	16.9 (14.4,19.3)	34	12.7 (7.3,18.1)
aPD (95% CI; q-value)	23.5 (14.8,32.1; <0.001)		11.8 (9.1,14.5; <0.001)		5.7 (-0.1,11.4; 0.12)	
To keep our borders open						
Never justified	159	72.0 (63.5,80.6)	1396	80.5 (77.9,83.1)	126	65.1 (56.1,74.0)
Sometimes justified	30	14.6 (7.4,21.8)	218	12.5 (10.4,14.7)	42	20.2 (12.7,27.7)
Usually or always justified	31	13.2 (7.1,19.4)	96	6.2 (4.7,7.8)	11	4.9 (1.8,8.0)
aPD (95% CI; q-value)	6.4 (0.0,12.8; 0.18)		2.9 (1.1,4.7; 0.02)		-0.1 (-4.0,3.7; 0.97)	

Table S4, continued.

What do you think about the use of force or violence in the following situations?	MAGA Supporter, Non-Republican		Non-MAGA, Republican		Unclassified	
	Unweighted n	Weighted % (95% CI)	Unweighted n	Weighted % (95% CI)	Unweighted n	Weighted % (95% CI)
To stop a protest or demonstration						
Never justified	129	60.2 (51.1,69.2)	995	59.7 (56.6,62.9)	106	50.7 (41.5,59.9)
Sometimes justified	67	26.3 (18.6,34.1)	613	33.5 (30.6,36.5)	52	31.4 (22.6,40.3)
Usually or always justified	23	13.2 (6.4,20.1)	102	6.3 (4.7,7.9)	19	8.0 (3.6,12.4)
aPD (95% CI; q-value)	6.7 (0.0,13.3; 0.20)		3.4 (1.6,5.3; 0.005)		3.2 (-1.8,8.2; 0.45)	
To support a protest or demonstration						
Never justified	159	69.3 (60.7,77.8)	1480	83.9 (81.4,86.3)	147	76.4 (68.5,84.4)
Sometimes justified	41	20.2 (12.5,27.9)	189	12.6 (10.3,14.8)	23	10.8 (5.6,16.0)
Usually or always justified	20	10.4 (5.1,15.6)	40	3.0 (1.9,4.2)	6	3.1 (-0.3,6.5)
aPD (95% CI; q-value)	4.7 (-0.4,9.9; 0.33)		0.8 (-0.6,2.2; 0.56)		-1.3 (-5.1,2.6; 0.74)	
To support women's reproductive rights						
Never justified	155	65.8 (57.0,74.6)	1437	80.7 (78.1,83.4)	138	68.9 (60.0,77.7)
Sometimes justified	35	16.3 (9.3,23.3)	173	11.8 (9.6,14.1)	23	15.2 (8.0,22.5)
Usually or always justified	30	17.7 (10.7,24.8)	99	6.6 (5.0,8.3)	16	7.3 (2.9,11.6)
aPD (95% CI; q-value)	6.7 (-0.8,14.1; 0.33)		-0.6 (-2.6,1.5; 0.77)		-1.8 (-7.0,3.3; 0.75)	
To support the right to life						
Never justified	137	61.7 (52.9,70.6)	1325	75.4 (72.5,78.2)	124	61.4 (52.3,70.5)
Sometimes justified	45	19.8 (12.8,26.7)	247	15.0 (12.7,17.3)	31	18.1 (10.7,25.4)
Usually or always justified	38	18.4 (11.2,25.6)	135	8.8 (6.9,10.7)	21	10.6 (5.2,16.1)
aPD (95% CI; q-value)	8.5 (1.2,15.9; 0.09)		3.1 (0.9,5.3; 0.03)		2.3 (-4.2,8.9; 0.65)	
To protect the environment or stop climate change						
Never justified	169	70.3 (61.7,79.0)	1461	82.4 (79.8,84.9)	141	71.5 (63.1,80.0)
Sometimes justified	26	17.3 (9.6,25.0)	181	11.8 (9.6,14.0)	24	12.0 (6.4,17.5)
Usually or always justified	25	12.2 (6.6,17.8)	67	5.2 (3.7,6.7)	12	6.9 (2.0,11.8)
aPD (95% CI; q-value)	1.6 (-4.1,7.2; 0.77)		-0.8 (-2.7,1.1; 0.66)		-0.7 (-6.7,5.3; 0.94)	
To protect the rights of animals						
Never justified	136	62.4 (53.6,71.3)	1236	70.0 (67.1,73.0)	116	60.7 (51.6,69.7)
Sometimes justified	48	19.1 (12.4,25.7)	348	20.3 (17.7,22.9)	30	16.0 (9.0,23.0)
Usually or always justified	36	18.4 (10.9,25.8)	126	9.0 (7.0,11.0)	32	14.3 (8.5,20.0)
aPD (95% CI; q-value)	5.8 (-1.5,13.2; 0.41)		1.6 (-0.7,3.9; 0.45)		4.3 (-2.2,10.8; 0.45)	

Adjusted prevalence differences (aPDs) are absolute percentage point (pp) differences for "usually or always justified" responses and are adjusted for age, race and ethnicity, gender, education, income, Census division, rurality, military history, number of non-traffic arrests, and total number of drinks per week. Q-values, also known as FDR-adjusted (or FDR-corrected) p-values, represent the probability that the given difference would be a false discovery; they represent the expected proportion of "false positives" that would be seen among the collection of all differences whose q-values were at or below the given q-value. Item non-responses are not reported in the tables but are included in the prevalence calculations.

Table S5. MAGA affiliation and personal willingness to commit political violence, by target of violence

In a situation where you think force or violence is justified to advance an important political objective…How willing would you personally be to use force or violence against a person because they are…	Non-MAGA, Non-Republican		MAGA Republican		MAGA Supporter, Republican	
	Unweighted n	Weighted % (95% CI)	Unweighted n	Weighted % (95% CI)	Unweighted n	Weighted % (95% CI)
An elected federal or state government official						
Not asked the question	1423	33.0 (31.1,34.8)	128	11.6 (9.2,14.1)	65	12.4 (8.6,16.1)
Not willing	3068	60.2 (58.3,62.1)	1091	78.0 (74.8,81.2)	525	79.5 (75.1,83.9)
Somewhat willing	148	4.3 (3.4,5.2)	91	7.2 (5.1,9.3)	43	5.4 (3.4,7.4)
Very or completely willing	68	1.9 (1.3,2.5)	25	2.0 (0.7,3.2)	8	1.3 (0.1,2.6)
aPD (95% CI; q-value)	Referent		0.4 (-0.6,1.3; 0.56)		0.4 (-1.0,1.9; 0.61)	
An elected local government official						
Not asked the question	1423	33.0 (31.1,34.8)	128	11.6 (9.2,14.1)	65	12.4 (8.6,16.1)
Not willing	3089	61.0 (59.1,62.9)	1091	78.1 (74.9,81.3)	536	80.4 (76.0,84.9)
Somewhat willing	139	3.8 (3.0,4.7)	86	6.7 (4.7,8.8)	37	5.1 (3.1,7.1)
Very or completely willing	58	1.8 (1.2,2.3)	25	2.2 (0.8,3.5)	4	0.7 (-0.3,1.8)
aPD (95% CI; q-value)	Referent		0.6 (-0.5,1.6; 0.59)		-0.3 (-1.6,1.0; 0.75)	
An election worker, such as a poll worker or vote counter						
Not asked the question	1423	33.0 (31.1,34.8)	128	11.6 (9.2,14.1)	65	12.4 (8.6,16.1)
Not willing	3162	62.4 (60.6,64.3)	1132	80.2 (77.1,83.3)	564	84.8 (80.7,88.9)
Somewhat willing	70	2.1 (1.5,2.8)	50	4.3 (2.8,5.9)	13	1.4 (0.5,2.3)
Very or completely willing	53	1.8 (1.2,2.4)	25	2.5 (1.0,3.9)	1	0.1 (-0.1,0.3)
aPD (95% CI; q-value)	Referent		1.0 (-0.2,2.2; 0.23)		-0.8 (-1.5,-0.1; 0.17)	
A public health official						
Not asked the question	1423	33.0 (31.1,34.8)	128	11.6 (9.2,14.1)	65	12.4 (8.6,16.1)
Not willing	3151	62.1 (60.2,64.0)	1103	78.0 (74.7,81.3)	539	80.7 (76.2,85.1)
Somewhat willing	80	2.7 (1.9,3.4)	72	5.6 (3.9,7.4)	31	3.9 (2.3,5.5)
Very or completely willing	48	1.5 (1.0,2.0)	25	3.2 (1.2,5.2)	7	1.8 (0.1,3.4)
aPD (95% CI; q-value)	Referent		1.9 (-0.0,3.9; 0.25)		1.1 (-0.8,3.0; 0.60)	
A member of the military or National Guard						
Not asked the question	1423	33.0 (31.1,34.8)	128	11.6 (9.2,14.1)	65	12.4 (8.6,16.1)
Not willing	3095	61.0 (59.1,62.9)	1118	78.5 (75.3,81.8)	558	83.3 (79.0,87.6)
Somewhat willing	130	3.7 (2.9,4.5)	56	5.3 (3.5,7.0)	17	2.7 (1.0,4.5)
Very or completely willing	58	1.7 (1.2,2.3)	26	2.9 (1.1,4.7)	2	0.2 (-0.1,0.5)
aPD (95% CI; q-value)	Referent		1.2 (-0.4,2.9; 0.46)		-0.8 (-1.6,-0.1; 0.26)	

Table S5, continued.

In a situation where you think force or violence is justified to advance an important political objective…How willing would <u>you personally</u> be to use force or violence against a person <u>because</u> they are…	Non-MAGA, Non-Republican		MAGA Republican		MAGA Supporter, Republican	
	**Unweighted n**	**Weighted % (95% CI)**	**Unweighted n**	**Weighted % (95% CI)**	**Unweighted n**	**Weighted % (95% CI)**
A police officer						
Not asked the question	1423	33.0 (31.1,34.8)	128	11.6 (9.2,14.1)	65	12.4 (8.6,16.1)
Not willing	3012	59.0 (57.1,60.9)	1132	79.5 (76.2,82.8)	552	82.7 (78.3,87.0)
Somewhat willing	198	5.2 (4.3,6.2)	51	5.2 (3.2,7.1)	21	2.9 (1.3,4.5)
Very or completely willing	80	2.4 (1.7,3.0)	22	2.4 (0.7,4.1)	4	0.7 (-0.3,1.8)
aPD (95% CI; q-value)	Referent		0.3 (-1.2,1.8; 0.83)		-0.8 (-2.1,0.6; 0.46)	
A person who does not share your race or ethnicity						
Not asked the question	1423	33.0 (31.1,34.8)	128	11.6 (9.2,14.1)	65	12.4 (8.6,16.1)
Not willing	3155	62.3 (60.5,64.2)	1135	80.1 (76.9,83.3)	560	83.7 (79.5,88.0)
Somewhat willing	89	2.7 (2.0,3.5)	51	4.2 (2.7,5.7)	16	2.0 (0.9,3.1)
Very or completely willing	42	1.4 (0.9,1.9)	18	2.8 (0.9,4.7)	1	0.6 (-0.5,1.7)
aPD (95% CI; q-value)	Referent		1.5 (-0.2,3.1; 0.35)		-0.3 (-1.7,1.1; 0.95)	
A person who does not share your religion						
Not asked the question	1423	33.0 (31.1,34.8)	128	11.6 (9.2,14.1)	65	12.4 (8.6,16.1)
Not willing	3162	62.4 (60.5,64.3)	1139	80.1 (76.9,83.3)	560	84.0 (79.8,88.2)
Somewhat willing	76	2.4 (1.7,3.1)	44	4.7 (2.7,6.6)	13	1.5 (0.5,2.5)
Very or completely willing	48	1.7 (1.1,2.2)	21	2.2 (0.8,3.6)	3	0.7 (-0.4,1.8)
aPD (95% CI; q-value)	Referent		0.6 (-0.6,1.8; 0.65)		-0.3 (-1.7,1.1; 0.92)	
A person who does not share your political beliefs						
Not asked the question	1423	33.0 (31.1,34.8)	128	11.6 (9.2,14.1)	65	12.4 (8.6,16.1)
Not willing	3095	60.6 (58.6,62.5)	1130	80.0 (76.9,83.1)	549	82.0 (77.6,86.4)
Somewhat willing	129	4.0 (3.1,4.9)	56	5.1 (3.3,6.8)	24	3.0 (1.6,4.4)
Very or completely willing	54	1.8 (1.2,2.3)	18	1.9 (0.6,3.2)	4	1.3 (-0.2,2.8)
aPD (95% CI; q-value)	Referent		0.8 (-0.7,2.3; 0.74)		0.3 (-1.4,2.1; 0.84)	

Table S5, continued.

**In a situation where you think force or violence is justified to advance an important political objective…How willing would you personally be to use force or violence against a person because they are…**	**MAGA Supporter, Non-Republican**		**Non-MAGA, Republican**		**Unclassified**	
	**Unweighted n**	**Weighted % (95% CI)**	**Unweighted n**	**Weighted % (95% CI)**	**Unweighted n**	**Weighted % (95% CI)**
An elected federal or state government official						
Not asked the question	42	25.0 (16.7,33.3)	325	23.2 (20.4,26.0)	88	47.2 (38.7,55.7)
Not willing	150	58.3 (49.0,67.6)	1304	70.8 (67.7,73.8)	128	45.4 (37.1,53.7)
Somewhat willing	13	6.8 (1.4,12.3)	57	3.7 (2.3,5.2)	4	3.0 (-0.7,6.7)
Very or completely willing	14	8.1 (2.8,13.4)	27	1.9 (0.9,2.9)	1	0.5 (-0.5,1.4)
aPD (95% CI; q-value)	4.8 (-0.6,10.1; 0.27)		0.8 (-0.3,1.9; 0.36)		-1.9 (-3.3,-0.5; 0.08)	
An elected local government official						
Not asked the question	42	25.0 (16.7,33.3)	325	23.2 (20.4,26.0)	88	47.2 (38.7,55.7)
Not willing	151	58.5 (49.2,67.8)	1309	71.0 (68.0,74.1)	126	44.7 (36.4,53.0)
Somewhat willing	10	7.6 (1.4,13.7)	57	3.7 (2.3,5.1)	4	2.9 (-0.8,6.5)
Very or completely willing	16	7.1 (2.8,11.4)	21	1.6 (0.7,2.6)	3	1.3 (-0.2,2.9)
aPD (95% CI; q-value)	4.1 (-0.2,8.3; 0.39)		0.5 (-0.6,1.6; 0.59)		-0.7 (-2.7,1.3; 0.66)	
An election worker, such as a poll worker or vote counter						
Not asked the question	42	25.0 (16.7,33.3)	325	23.2 (20.4,26.0)	88	47.2 (38.7,55.7)
Not willing	151	57.6 (48.2,66.9)	1333	72.5 (69.5,75.5)	130	47.9 (39.5,56.3)
Somewhat willing	11	5.3 (1.7,8.9)	40	2.9 (1.6,4.1)	.	. (.,.)
Very or completely willing	15	10.3 (3.5,17.1)	11	1.0 (0.3,1.6)	2	0.9 (-0.4,2.1)
aPD (95% CI; q-value)	7.1 (0.3,13.9; 0.17)		0.0 (-0.9,0.9; 0.99)		-1.1 (-2.8,0.5; 0.37)	
A public health official						
Not asked the question	42	25.0 (16.7,33.3)	325	23.2 (20.4,26.0)	88	47.2 (38.7,55.7)
Not willing	151	57.7 (48.4,67.1)	1327	71.9 (69.0,74.9)	127	45.3 (37.0,53.6)
Somewhat willing	10	6.8 (1.2,12.5)	41	2.7 (1.6,3.8)	3	2.7 (-0.9,6.3)
Very or completely willing	16	8.6 (3.3,13.9)	19	1.7 (0.7,2.7)	2	0.9 (-0.4,2.1)
aPD (95% CI; q-value)	5.9 (0.4,11.3; 0.22)		0.9 (-0.3,2.0; 0.47)		-1.0 (-2.6,0.6; 0.60)	
A member of the military or National Guard						
Not asked the question	42	25.0 (16.7,33.3)	325	23.2 (20.4,26.0)	88	47.2 (38.7,55.7)
Not willing	151	59.7 (50.4,68.9)	1312	71.1 (68.1,74.1)	127	47.5 (39.1,55.9)
Somewhat willing	7	3.2 (0.5,5.9)	53	3.8 (2.4,5.1)	4	0.9 (-0.2,2.0)
Very or completely willing	19	10.3 (4.0,16.7)	19	1.5 (0.5,2.4)	2	0.9 (-0.4,2.1)
aPD (95% CI; q-value)	7.2 (0.9,13.5; 0.26)		0.5 (-0.6,1.6; 0.79)		-1.2 (-2.9,0.4; 0.46)	

Table S5, continued.

In a situation where you think force or violence is justified to advance an important political objective…How willing would you personally be to use force or violence against a person because they are…	MAGA Supporter, Non-Republican		Non-MAGA, Republican		Unclassified	
	Unweighted n	Weighted % (95% CI)	Unweighted n	Weighted % (95% CI)	Unweighted n	Weighted % (95% CI)
A police officer						
Not asked the question	42	25.0 (16.7,33.3)	325	23.2 (20.4,26.0)	88	47.2 (38.7,55.7)
Not willing	153	58.8 (49.4,68.1)	1311	70.6 (67.6,73.7)	125	46.4 (38.0,54.8)
Somewhat willing	7	6.7 (0.6,12.9)	56	4.2 (2.7,5.6)	5	1.5 (-0.0,2.9)
Very or completely willing	16	6.6 (2.5,10.7)	20	1.6 (0.6,2.5)	1	0.5 (-0.5,1.6)
aPD (95% CI; q-value)	2.8 (-1.5,7.0; 0.44)		0.0 (-1.1,1.2; 0.97)		-2.1 (-3.6,-0.6; 0.08)	
A person who does not share your race or ethnicity						
Not asked the question	42	25.0 (16.7,33.3)	325	23.2 (20.4,26.0)	88	47.2 (38.7,55.7)
Not willing	152	60.1 (50.9,69.4)	1323	72.2 (69.2,75.2)	129	47.2 (38.8,55.6)
Somewhat willing	8	4.2 (-0.8,9.1)	45	2.8 (1.6,3.9)	1	0.4 (-0.4,1.2)
Very or completely willing	16	8.3 (3.5,13.1)	17	1.3 (0.5,2.2)	3	1.3 (-0.2,2.9)
aPD (95% CI; q-value)	5.3 (0.5,10.1; 0.17)		0.5 (-0.4,1.5; 0.68)		-0.4 (-2.4,1.5; 0.95)	
A person who does not share your religion						
Not asked the question	42	25.0 (16.7,33.3)	325	23.2 (20.4,26.0)	88	47.2 (38.7,55.7)
Not willing	156	62.5 (53.3,71.7)	1333	72.5 (69.6,75.5)	129	47.7 (39.3,56.1)
Somewhat willing	6	1.5 (0.1,3.0)	37	2.5 (1.4,3.6)	1	0.5 (-0.5,1.6)
Very or completely willing	15	9.1 (3.0,15.3)	14	1.3 (0.4,2.1)	2	0.8 (-0.3,1.9)
aPD (95% CI; q-value)	6.0 (-0.2,12.2; 0.27)		0.2 (-0.9,1.2; 0.92)		-1.4 (-3.0,0.2; 0.30)	
A person who does not share your political beliefs						
Not asked the question	42	25.0 (16.7,33.3)	325	23.2 (20.4,26.0)	88	47.2 (38.7,55.7)
Not willing	153	59.0 (49.7,68.4)	1329	72.2 (69.2,75.2)	127	47.0 (38.6,55.3)
Somewhat willing	11	9.0 (2.3,15.6)	42	2.6 (1.5,3.7)	3	0.6 (-0.1,1.3)
Very or completely willing	13	5.2 (1.6,8.8)	15	1.5 (0.5,2.5)	3	1.3 (-0.2,2.9)
aPD (95% CI; q-value)	1.8 (-1.8,5.4; 0.74)		0.5 (-0.7,1.6; 0.75)		-0.8 (-2.8,1.2; 0.75)	

Adjusted prevalence differences (aPDs) are absolute percentage point (pp) differences for “very or completely willing” responses and are adjusted for age, race and ethnicity, gender, education, income, Census division, rurality, military history, number of non-traffic arrests, and total number of drinks per week. Q-values, also known as FDR-adjusted (or FDR-corrected) p-values, represent the probability that the given difference would be a false discovery; they represent the expected proportion of “false positives” that would be seen among the collection of all differences whose q-values were at or below the given q-value. Item non-responses are not reported in the tables but are included in the prevalence calculations.

Table S6. MAGA affiliation and personal willingness to commit political violence, by social context of violence

You agreed that the use of force or violence could be justified to advance (one/some) of the political objectives we just discussed. In (that/those) (situation/situations), how willing would you personally be to…	Non-MAGA, Non-Republican		MAGA Republican		MAGA Supporter, Republican	
	Unweighted n	Weighted % (95% CI)	Unweighted n	Weighted % (95% CI)	Unweighted n	Weighted % (95% CI)
Use force or violence as part of a group of people who share your beliefs						
Not asked the question	1423	33.0 (31.1,34.8)	128	11.6 (9.2,14.1)	65	12.4 (8.6,16.1)
Not willing	2798	54.8 (52.9,56.8)	896	64.9 (61.3,68.4)	435	66.3 (61.1,71.5)
Sometimes willing	419	9.5 (8.3,10.7)	261	18.7 (15.8,21.7)	130	17.5 (13.5,21.6)
Very or completely willing	68	2.0 (1.4,2.7)	49	3.4 (2.2,4.5)	11	2.4 (0.7,4.2)
aPD (95% CI; q-value)	Referent		1.9 (0.5,3.3; 0.07)		1.0 (-1.0,3.1; 0.68)	
Use force or violence on your own, as an individual						
Not asked the question	1423	33.0 (31.1,34.8)	128	11.6 (9.2,14.1)	65	12.4 (8.6,16.1)
Not willing	2647	52.8 (50.9,54.7)	836	60.2 (56.6,63.8)	410	61.6 (56.3,66.9)
Sometimes willing	527	10.8 (9.5,12.0)	285	19.9 (17.0,22.8)	142	20.5 (16.0,24.9)
Very or completely willing	110	2.8 (2.1,3.5)	86	6.9 (4.8,9.0)	26	4.3 (2.2,6.3)
aPD (95% CI; q-value)	Referent		5.7 (3.4,8.1; <0.001)		2.8 (0.5,5.1; 0.08)	
Organize a group of people who share your beliefs to use force or violence						
Not asked the question	1423	33.0 (31.1,34.8)	128	11.6 (9.2,14.1)	65	12.4 (8.6,16.1)
Not willing	2988	59.1 (57.2,61.0)	1012	71.4 (68.0,74.9)	495	73.1 (67.7,78.4)
Sometimes willing	247	5.5 (4.6,6.5)	156	12.4 (9.7,15.1)	72	10.9 (6.8,15.0)
Very or completely willing	55	1.8 (1.2,2.4)	39	3.1 (1.9,4.4)	10	2.4 (0.4,4.3)
aPD (95% CI; q-value)	Referent		2.2 (0.7,3.6; 0.05)		1.3 (-0.9,3.5; 0.55)	

Table S6, continued.

**You agreed that the use of force or violence could be justified to advance (one/some) of the political objectives we just discussed. In (that/those) (situation/situations), how willing would you personally be to…**	**MAGA Supporter, Non-Republican**		**Non-MAGA, Republican**		**Unclassified**	
	**Unweighted n**	**Weighted % (95% CI)**	**Unweighted n**	**Weighted % (95% CI)**	**Unweighted n**	**Weighted % (95% CI)**
Use force or violence as part of a group of people who share your beliefs						
Not asked the question	42	25.0 (16.7,33.3)	325	23.2 (20.4,26.0)	88	47.2 (38.7,55.7)
Not willing	128	52.3 (43.0,61.5)	1171	64.2 (61.0,67.3)	120	45.2 (36.9,53.6)
Sometimes willing	33	14.5 (7.5,21.4)	191	10.0 (8.0,11.9)	8	2.5 (0.3,4.7)
Very or completely willing	16	6.2 (2.2,10.2)	30	2.5 (1.3,3.7)	4	1.1 (-0.1,2.4)
aPD (95% CI; q-value)	2.8 (-1.2,6.8; 0.57)		0.8 (-0.5,2.1; 0.61)		-1.0 (-2.7,0.7; 0.61)	
Use force or violence on your own, as an individual						
Not asked the question	42	25.0 (16.7,33.3)	325	23.2 (20.4,26.0)	88	47.2 (38.7,55.7)
Not willing	116	46.0 (37.0,55.1)	1076	59.9 (56.8,63.1)	114	42.5 (34.2,50.7)
Sometimes willing	35	13.0 (7.3,18.8)	259	12.8 (10.7,14.8)	14	4.3 (1.5,7.0)
Very or completely willing	26	13.9 (6.3,21.5)	55	3.5 (2.1,4.9)	5	2.7 (0.2,5.2)
aPD (95% CI; q-value)	9.8 (2.7,17.0; 0.05)		2.1 (0.4,3.7; 0.08)		-0.0 (-2.9,2.9; 0.99)	
Organize a group of people who share your beliefs to use force or violence						
Not asked the question	42	25.0 (16.7,33.3)	325	23.2 (20.4,26.0)	88	47.2 (38.7,55.7)
Not willing	141	57.1 (48.0,66.3)	1273	68.2 (65.1,71.3)	125	45.1 (36.8,53.4)
Sometimes willing	21	7.3 (3.4,11.2)	98	6.4 (4.7,8.2)	7	4.1 (0.6,7.6)
Very or completely willing	15	8.5 (3.2,13.8)	22	2.0 (0.9,3.2)	2	0.8 (-0.3,1.9)
aPD (95% CI; q-value)	5.4 (-0.1,10.9; 0.31)		0.7 (-0.6,2.0; 0.55)		-1.1 (-2.6,0.5; 0.51)	

Adjusted prevalence differences (aPDs) are absolute percentage point (pp) differences for "very or completely willing" responses and are adjusted for age, race and ethnicity, gender, education, income, Census division, rurality, military history, number of non-traffic arrests, and total number of drinks per week. Q-values, also known as FDR-adjusted (or FDR-corrected) p-values, represent the probability that the given difference would be a false discovery; they represent the expected proportion of "false positives" that would be seen among the collection of all differences whose q-values were at or below the given q-value. Item non-responses are not reported in the tables but are included in the prevalence calculations.

Table S7. MAGA affiliation and personal willingness to commit election-year violence

**This is an election year. How willing would you personally be to use force or violence to make sure your preferred candidate for President takes office in each of the following situations?**	**Non-MAGA, Non-Republican**		**MAGA Republican**		**MAGA Supporter, Republican**	
	**Unweighted n**	**Weighted % (95% CI)**	**Unweighted n**	**Weighted % (95% CI)**	**Unweighted n**	**Weighted % (95% CI)**
Another candidate is declared the winner, and you believe the election is being stolen						
Not asked the question	1423	33.0 (31.1,34.8)	128	11.6 (9.2,14.1)	65	12.4 (8.6,16.1)
Not willing	3073	60.8 (58.9,62.7)	968	70.7 (67.4,74.1)	491	73.4 (68.3,78.4)
Sometimes willing	166	4.2 (3.4,5.1)	181	11.8 (9.7,14.0)	78	11.6 (7.9,15.4)
Very or completely willing	48	1.3 (0.9,1.8)	55	4.2 (2.5,5.9)	8	1.5 (0.2,2.8)
aPD (95% CI; q-value)	Referent		3.9 (2.1,5.7; 0.001)		1.0 (-0.6,2.6; 0.46)	
Another candidate wins legitimately						
Not asked the question	1423	33.0 (31.1,34.8)	128	11.6 (9.2,14.1)	65	12.4 (8.6,16.1)
Not willing	3132	62.0 (60.1,63.8)	1146	80.6 (77.4,83.8)	563	84.2 (79.9,88.4)
Sometimes willing	91	2.6 (1.9,3.3)	36	3.9 (2.0,5.7)	8	1.6 (0.1,3.1)
Very or completely willing	63	1.9 (1.3,2.5)	23	2.4 (1.0,3.8)	7	0.7 (0.1,1.4)
aPD (95% CI; q-value)	Referent		0.6 (-0.7,1.9; 0.58)		-0.3 (-1.2,0.6; 0.66)	

Table S7, continued.

**This is an election year. How willing would <u>you personally</u> be to use force or violence to make sure your preferred candidate for President takes office in each of the following situations?**	**MAGA Supporter, Non-Republican**		**Non-MAGA, Republican**		**Unclassified**	
	**Unweighted n**	**Weighted % (95% CI)**	**Unweighted n**	**Weighted % (95% CI)**	**Unweighted n**	**Weighted % (95% CI)**
Another candidate is declared the winner, and you believe the election is being stolen						
Not asked the question	42	25.0 (16.7,33.3)	325	23.2 (20.4,26.0)	88	47.2 (38.7,55.7)
Not willing	139	53.3 (44.0,62.6)	1295	71.0 (68.0,74.0)	127	47.4 (39.0,55.8)
Sometimes willing	18	7.8 (2.2,13.4)	84	4.2 (3.0,5.5)	4	1.0 (-0.2,2.1)
Very or completely willing	18	11.6 (4.6,18.6)	13	1.4 (0.5,2.3)	2	0.7 (-0.3,1.6)
aPD (95% CI; q-value)	9.1 (2.2,16.1; 0.06)		0.7 (-0.3,1.8; 0.42)		-0.9 (-2.2,0.4; 0.42)	
Another candidate wins legitimately						
Not asked the question	42	25.0 (16.7,33.3)	325	23.2 (20.4,26.0)	88	47.2 (38.7,55.7)
Not willing	153	59.9 (50.6,69.2)	1343	72.6 (69.6,75.6)	127	46.5 (38.2,54.9)
Sometimes willing	8	5.1 (-0.2,10.3)	29	2.5 (1.3,3.7)	6	1.7 (0.1,3.3)
Very or completely willing	14	7.7 (2.5,12.9)	19	1.4 (0.6,2.2)	2	1.5 (-0.9,4.0)
aPD (95% CI; q-value)	4.1 (-1.2,9.5; 0.30)		0.4 (-0.6,1.4; 0.66)		-0.8 (-3.7,2.1; 0.69)	

Adjusted prevalence differences (aPDs) are absolute percentage point (pp) differences for “very or completely willing” responses and are adjusted for age, race and ethnicity, gender, education, income, Census division, rurality, military history, number of non-traffic arrests, and total number of drinks per week. Q-values, also known as FDR-adjusted (or FDR-corrected) p-values, represent the probability that the given difference would be a false discovery; they represent the expected proportion of “false positives” that would be seen among the collection of all differences whose q-values were at or below the given q-value. Item non-responses are not reported in the tables but are included in the prevalence calculations.

Table S8. MAGA affiliation and beliefs concerning democracy and authoritarianism

Statement or Query	Non-MAGA, Non-Republican		MAGA Republican		MAGA Supporter, Republican	
	Unweighted n	Weighted % (95% CI)	Unweighted n	Weighted % (95% CI)	Unweighted n	Weighted % (95% CI)
When thinking about democracy in the United States these days, do you believe…						
There is a serious threat to our democracy.	3579	68.3 (66.4,70.1)	1035	74.1 (70.6,77.5)	427	61.8 (56.4,67.1)
There may be a threat to our democracy, but it is not serious.	907	24.2 (22.5,26.0)	207	18.8 (15.6,22.1)	158	28.6 (23.4,33.7)
There is no threat to our democracy.	208	6.4 (5.4,7.5)	99	6.6 (4.8,8.4)	56	9.2 (6.2,12.1)
aPD (95% CI; q-value)	Referent		-0.5 (-4.5,3.5; 0.84)		-12.4 (-18.0,-6.9; <0.001)	
How much is each of the following a threat to democracy in the United States?						
The influence of big money in elections						
A serious threat	3574	71.4 (69.6,73.2)	968	68.8 (65.3,72.3)	447	68.6 (63.7,73.6)
A threat, but not a serious threat	997	23.1 (21.4,24.7)	319	25.1 (21.9,28.4)	166	25.8 (21.1,30.4)
Not a threat	122	4.2 (3.2,5.1)	55	5.7 (3.7,7.6)	27	4.2 (2.2,6.1)
aPD (95% CI; q-value)	Referent		-6.6 (-10.6,-2.5; 0.02)		-5.0 (-10.4,0.3; 0.18)	
The influence of foreign governments in elections						
A serious threat	3054	58.4 (56.4,60.3)	809	58.7 (55.1,62.3)	316	49.4 (44.0,54.7)
A threat, but not a serious threat	1378	31.9 (30.1,33.8)	435	32.4 (29.0,35.8)	259	38.7 (33.6,43.9)
Not a threat	265	8.3 (7.1,9.5)	98	8.6 (6.3,10.8)	66	10.6 (7.5,13.7)
aPD (95% CI; q-value)	Referent		-4.5 (-8.8,-0.3; 0.23)		-12.5 (-18.4,-6.7; <0.001)	
The possibility of political violence						
A serious threat	3120	62.1 (60.2,64.0)	549	39.8 (36.3,43.3)	204	28.5 (23.8,33.1)
A threat, but not a serious threat	1397	31.2 (29.4,33.0)	630	48.0 (44.4,51.6)	360	59.2 (54.1,64.4)
Not a threat	176	5.3 (4.3,6.2)	161	11.5 (9.2,13.8)	79	11.2 (8.3,14.2)
aPD (95% CI; q-value)	Referent		-23.6 (-27.8,-19.5; <0.001)		-33.8 (-38.9,-28.6; <0.001)	
The influence of white nationalist groups						
A serious threat	3168	63.4 (61.5,65.3)	219	16.7 (14.0,19.4)	85	12.9 (9.5,16.2)
A threat, but not a serious threat	1208	27.1 (25.4,28.8)	448	34.9 (31.4,38.5)	233	37.5 (32.1,42.9)
Not a threat	316	8.0 (6.9,9.2)	672	47.8 (44.2,51.4)	322	47.5 (42.2,52.8)
aPD (95% CI; q-value)	Referent		-46.3 (-49.9,-42.8; <0.001)		-49.4 (-53.5,-45.3; <0.001)	
The influence of groups like Black Lives Matter						
A serious threat	459	11.0 (9.7,12.3)	814	56.2 (52.5,59.8)	313	47.2 (41.8,52.5)
A threat, but not a serious threat	1298	27.4 (25.7,29.2)	400	31.7 (28.2,35.1)	258	39.5 (34.4,44.7)
Not a threat	2935	60.1 (58.2,62.0)	128	11.8 (9.1,14.5)	71	12.1 (8.8,15.4)
aPD (95% CI; q-value)	Referent		43.0 (39.0,47.1; <0.001)		36.0 (30.6,41.5; <0.001)	
Efforts to overturn the results of elections						
A serious threat	3665	71.1 (69.3,72.9)	563	43.4 (39.8,47.0)	189	30.9 (25.7,36.1)
A threat, but not a serious threat	802	20.5 (18.9,22.1)	443	33.7 (30.1,37.2)	255	37.5 (32.5,42.6)
Not a threat	232	7.1 (5.9,8.2)	329	21.8 (19.0,24.6)	198	29.7 (25.0,34.4)
aPD (95% CI; q-value)	Referent		-30.2 (-34.4,-26.0; <0.001)		-42.2 (-47.9,-36.5; <0.001)	

Table S8, continued.

Statement or Query	Non-MAGA, Non-Republican		MAGA Republican		MAGA Supporter, Republican	
	Unweighted n	Weighted % (95% CI)	Unweighted n	Weighted % (95% CI)	Unweighted n	Weighted % (95% CI)
How important do you think it is for the United States to remain a democracy?						
Not important	85	3.4 (2.5,4.3)	42	3.7 (2.1,5.2)	16	3.4 (1.4,5.5)
Somewhat important	210	7.6 (6.4,8.8)	62	7.5 (5.2,9.8)	17	3.7 (1.6,5.8)
Very or extremely important	4414	88.3 (86.9,89.8)	1230	87.4 (84.7,90.2)	606	91.9 (88.9,94.8)
aPD (95% CI; q-value)	Referent		-1.4 (-3.4,0.7; 0.50)		-1.2 (-3.6,1.3; 0.56)	
Democracy is the best form of government.						
Do not agree	192	6.2 (5.1,7.2)	117	9.7 (7.4,12.0)	49	8.9 (5.9,11.8)
Somewhat agree	814	22.3 (20.5,24.0)	197	18.5 (15.4,21.6)	88	18.9 (14.0,23.7)
Strongly or very strongly agree	3680	70.0 (68.1,71.9)	1018	70.4 (66.9,73.9)	506	72.0 (66.8,77.2)
aPD (95% CI; q-value)	Referent		-6.0 (-9.9,-2.1; 0.007)		-6.8 (-12.2,-1.5; 0.02)	
These days, American democracy only serves the interests of the wealthy and powerful.						
Do not agree	1682	30.0 (28.3,31.6)	517	34.1 (30.8,37.5)	262	37.4 (32.4,42.4)
Somewhat agree	1831	39.6 (37.7,41.5)	413	33.2 (29.7,36.7)	225	34.4 (29.3,39.5)
Strongly or very strongly agree	1183	29.4 (27.5,31.2)	408	31.8 (28.4,35.2)	156	27.7 (22.6,32.8)
aPD (95% CI; q-value)	Referent		5.6 (1.7,9.5; 0.02)		2.5 (-2.7,7.8; 0.56)	
Having a strong leader for America is more important than having a democracy.						
Do not agree	3619	69.6 (67.8,71.5)	699	45.7 (42.1,49.3)	428	62.9 (57.5,68.2)
Somewhat agree	599	16.9 (15.3,18.5)	275	24.9 (21.6,28.3)	108	18.4 (14.0,22.9)
Strongly or very strongly agree	461	11.9 (10.6,13.2)	358	28.1 (24.8,31.4)	105	18.2 (13.7,22.7)
aPD (95% CI; q-value)	Referent		16.8 (13.1,20.4; <0.001)		8.7 (4.0,13.3; 0.002)	
We should suspend Congress for a few years so a strong leader can clean up the mess made by politicians in Washington.						
Do not agree	3641	70.1 (68.2,71.9)	789	52.9 (49.3,56.6)	456	62.6 (57.1,68.1)
Somewhat agree	633	17.3 (15.7,18.9)	237	20.1 (17.1,23.2)	100	19.3 (14.6,23.9)
Strongly or very strongly agree	413	11.4 (10.0,12.7)	313	26.0 (22.7,29.2)	84	16.8 (12.4,21.3)
aPD (95% CI; q-value)	Referent		15.7 (12.2,19.3; <0.001)		9.1 (4.4,13.9; <0.001)	
I can trust the American people as a whole when it comes to making judgments under our democratic system about the issues facing our country.						
Do not agree	899	21.7 (20.1,23.4)	284	22.9 (19.7,26.1)	134	22.8 (18.0,27.5)
Somewhat agree	2528	54.2 (52.3,56.1)	628	47.0 (43.3,50.6)	318	51.0 (45.6,56.3)
Strongly or very strongly agree	1274	23.1 (21.6,24.7)	425	29.0 (25.8,32.2)	190	25.2 (21.0,29.4)
aPD (95% CI; q-value)	Referent		2.8 (-0.9,6.6; 0.58)		-0.6 (-5.0,3.8; 0.90)	

Table S8, continued.

Statement or Query	MAGA Supporter, Non-Republican		Non-MAGA, Republican		Unclassified	
	Unweighted n	Weighted % (95% CI)	Unweighted n	Weighted % (95% CI)	Unweighted n	Weighted % (95% CI)
When thinking about democracy in the United States these days, do you believe…						
There is a serious threat to our democracy.	138	55.9 (46.7,65.2)	963	54.9 (51.7,58.1)	114	37.9 (30.0,45.7)
There may be a threat to our democracy, but it is not serious.	53	28.6 (20.0,37.2)	582	36.0 (32.8,39.1)	46	19.5 (13.1,25.9)
There is no threat to our democracy.	31	15.5 (8.6,22.4)	168	8.7 (7.0,10.5)	11	7.4 (1.9,12.9)
aPD (95% CI; q-value)	-10.1 (-20.0,-0.3; 0.18)		-17.0 (-20.7,-13.3; <0.001)		-10.4 (-20.8,0.1; 0.19)	
How much is each of the following a threat to democracy in the United States?						
The influence of big money in elections						
A serious threat	154	72.7 (64.7,80.8)	1101	63.6 (60.5,66.7)	125	45.9 (37.5,54.2)
A threat, but not a serious threat	53	18.8 (12.0,25.6)	528	30.9 (27.9,33.9)	45	19.6 (13.0,26.2)
Not a threat	13	7.2 (2.0,12.4)	85	5.0 (3.7,6.4)	3	1.1 (-0.2,2.4)
aPD (95% CI; q-value)	4.7 (-3.8,13.3; 0.50)		-10.7 (-14.4,-7.1; <0.001)		-5.2 (-14.9,4.6; 0.50)	
The influence of foreign governments in elections						
A serious threat	114	50.8 (41.6,60.0)	914	51.4 (48.2,54.6)	107	35.0 (27.5,42.5)
A threat, but not a serious threat	77	35.3 (26.5,44.0)	664	38.9 (35.8,42.1)	52	23.5 (16.2,30.8)
Not a threat	27	12.0 (6.3,17.7)	135	8.7 (6.9,10.6)	13	7.7 (2.4,13.0)
aPD (95% CI; q-value)	-6.1 (-15.8,3.6; 0.55)		-8.9 (-12.7,-5.1; <0.001)		-9.9 (-20.0,0.2; 0.25)	
The possibility of political violence						
A serious threat	88	43.5 (34.2,52.7)	724	42.6 (39.4,45.8)	95	36.4 (28.3,44.4)
A threat, but not a serious threat	97	41.5 (32.5,50.5)	814	47.6 (44.4,50.8)	65	25.7 (18.6,32.9)
Not a threat	35	14.2 (8.0,20.4)	173	9.1 (7.3,10.8)	11	3.9 (0.9,6.8)
aPD (95% CI; q-value)	-16.6 (-26.2,-7.1; 0.004)		-20.0 (-23.8,-16.3; <0.001)		-11.2 (-21.9,-0.5; 0.15)	
The influence of white nationalist groups						
A serious threat	63	36.2 (27.1,45.3)	471	27.1 (24.3,29.9)	66	25.7 (18.4,32.9)
A threat, but not a serious threat	68	31.6 (22.8,40.4)	757	44.7 (41.5,47.8)	69	25.9 (18.9,32.9)
Not a threat	91	32.2 (24.1,40.2)	482	27.3 (24.4,30.2)	29	11.6 (6.1,17.1)
aPD (95% CI; q-value)	-25.6 (-35.2,-16.1; <0.001)		-35.4 (-38.9,-32.0; <0.001)		-26.7 (-37.6,-15.8; <0.001)	
The influence of groups like Black Lives Matter						
A serious threat	81	33.1 (24.3,41.8)	576	32.6 (29.6,35.5)	49	15.3 (9.9,20.7)
A threat, but not a serious threat	91	39.5 (30.5,48.4)	756	42.7 (39.5,45.8)	70	28.8 (21.3,36.3)
Not a threat	49	27.1 (18.9,35.2)	379	23.8 (21.1,26.6)	51	22.7 (15.6,29.8)
aPD (95% CI; q-value)	20.8 (12.0,29.6; <0.001)		21.9 (18.5,25.2; <0.001)		6.0 (-1.7,13.7; 0.29)	
Efforts to overturn the results of elections						
A serious threat	91	47.4 (38.2,56.7)	776	47.1 (43.9,50.3)	107	39.8 (31.8,47.9)
A threat, but not a serious threat	76	31.4 (23.0,39.8)	658	37.1 (34.0,40.2)	53	23.4 (16.0,30.8)
Not a threat	53	20.4 (13.3,27.6)	276	14.8 (12.6,17.1)	8	1.6 (0.3,3.0)
aPD (95% CI; q-value)	-21.1 (-30.9,-11.3; <0.001)		-25.3 (-29.1,-21.5; <0.001)		-13.3 (-24.0,-2.6; 0.08)	

Table S8, continued.

Statement or Query	MAGA Supporter, Non-Republican		Non-MAGA, Republican		Unclassified	
	Unweighted n	Weighted % (95% CI)	Unweighted n	Weighted % (95% CI)	Unweighted n	Weighted % (95% CI)
How important do you think it is for the United States to remain a democracy?						
Not important	11	5.7 (1.8,9.7)	28	2.7 (1.5,3.9)	11	6.3 (1.8,10.8)
Somewhat important	22	18.3 (10.0,26.6)	81	6.6 (4.8,8.4)	19	11.5 (5.8,17.2)
Very or extremely important	185	74.5 (65.7,83.2)	1608	90.6 (88.5,92.7)	165	60.5 (52.0,69.1)
aPD (95% CI; q-value)	-2.5 (-8.2,3.1; 0.56)		0.1 (-1.4,1.7; 0.98)		-4.6 (-11.4,2.2; 0.50)	
Democracy is the best form of government.						
Do not agree	26	15.2 (7.6,22.7)	86	6.3 (4.6,8.0)	23	11.3 (5.9,16.6)
Somewhat agree	57	33.9 (25.0,42.9)	306	22.2 (19.3,25.0)	45	23.8 (16.2,31.3)
Strongly or very strongly agree	137	50.6 (41.3,59.8)	1320	70.8 (67.7,73.9)	114	38.2 (30.4,46.0)
aPD (95% CI; q-value)	-15.8 (-25.0,-6.6; 0.003)		-5.0 (-8.5,-1.6; 0.01)		-14.5 (-24.8,-4.2; 0.01)	
These days, American democracy only serves the interests of the wealthy and powerful.						
Do not agree	72	28.6 (20.3,36.9)	716	37.7 (34.7,40.8)	52	18.8 (13.0,24.7)
Somewhat agree	62	25.8 (18.2,33.4)	624	35.8 (32.8,38.9)	84	31.7 (24.0,39.3)
Strongly or very strongly agree	88	45.6 (36.3,54.8)	374	26.0 (23.1,28.9)	50	24.1 (16.6,31.6)
aPD (95% CI; q-value)	13.0 (3.6,22.4; 0.03)		-0.3 (-3.8,3.1; 0.90)		1.6 (-8.2,11.5; 0.84)	
Having a strong leader for America is more important than having a democracy.						
Do not agree	118	47.8 (38.6,57.0)	1125	61.5 (58.3,64.6)	87	30.0 (22.7,37.3)
Somewhat agree	48	23.5 (15.9,31.1)	322	18.8 (16.3,21.3)	51	25.1 (17.9,32.4)
Strongly or very strongly agree	54	28.2 (19.6,36.8)	265	18.9 (16.2,21.6)	37	14.9 (8.7,21.1)
aPD (95% CI; q-value)	13.7 (4.5,22.8; 0.02)		9.3 (6.3,12.3; <0.001)		7.3 (-2.0,16.6; 0.18)	
We should suspend Congress for a few years so a strong leader can clean up the mess made by politicians in Washington.						
Do not agree	117	47.3 (38.2,56.5)	1190	63.6 (60.5,66.8)	87	30.2 (23.0,37.5)
Somewhat agree	40	19.4 (12.4,26.3)	295	19.3 (16.7,21.8)	61	32.0 (23.9,40.1)
Strongly or very strongly agree	63	32.9 (23.9,42.0)	231	16.6 (14.1,19.1)	28	9.4 (5.1,13.7)
aPD (95% CI; q-value)	18.9 (9.7,28.1; <0.001)		7.8 (5.0,10.6; <0.001)		-0.9 (-7.7,6.0; 0.87)	
I can trust the American people as a whole when it comes to making judgments under our democratic system about the issues facing our country.						
Do not agree	49	26.1 (17.9,34.2)	375	23.2 (20.5,25.9)	40	19.6 (12.9,26.4)
Somewhat agree	100	45.3 (36.2,54.5)	886	53.5 (50.3,56.6)	86	36.1 (28.0,44.2)
Strongly or very strongly agree	72	28.1 (19.8,36.4)	456	22.9 (20.3,25.5)	51	16.4 (10.8,22.1)
aPD (95% CI; q-value)	3.5 (-4.6,11.6; 0.73)		-1.4 (-4.5,1.7; 0.72)		0.4 (-7.7,8.5; 0.95)	

Adjusted prevalence (aPDs) are absolute percentage point (pp) differences for the following responses: items 1-7, “A serious threat”; item 8, “Very or extremely important”; items 9-14, “strongly or very strongly agree.” They are adjusted for age, race and ethnicity, gender, education, income, Census division, rurality, military history, number of non-traffic arrests, and total number of drinks per week. Q-values, also known as FDR-adjusted (or FDR-corrected) p-values, represent the probability that the given difference would be a false discovery; they represent the expected proportion of “false positives” that would be seen among the collection of all differences whose q-values were at or below the given q-value. Item non-responses are not reported in the tables but are included in the prevalence calculations.

Table S9. MAGA affiliation and beliefs concerning elections and partisanship

Statement or Query	Non-MAGA, Non-Republican		MAGA Republican		MAGA Supporter, Republican	
	Unweighted n	Weighted % (95% CI)	Unweighted n	Weighted % (95% CI)	Unweighted n	Weighted % (95% CI)
Which is more important to you?						
Having election outcomes determined democratically	3508	65.5 (63.6,67.5)	748	51.7 (48.1,55.3)	392	58.3 (52.9,63.7)
OR						
Having political leaders I can trust to look out for my values and interests	1071	29.1 (27.3,31.0)	575	46.3 (42.6,49.9)	242	40.3 (35.0,45.7)
aPD (95% CI; q-value)	Referent		-19.2 (-23.5,-14.9; <0.001)		-15.4 (-21.2,-9.6; <0.001)	
Which is more important to you?						
Allowing people the personal freedom to do as they please	704	16.0 (14.6,17.5)	456	33.4 (30.0,36.9)	267	41.6 (36.4,46.9)
OR						
Having people work together for a better country	3880	79.1 (77.5,80.8)	866	64.5 (61.0,68.0)	366	56.9 (51.6,62.2)
aPD (95% CI; q-value)	Referent		-19.3 (-23.2,-15.3; <0.001)		-24.5 (-30.0,-19.0; <0.001)	
Which is more important to you?						
Making sure that everyone who wants to vote can do so	3277	69.4 (67.6,71.1)	71	9.4 (6.8,12.1)	39	8.4 (5.0,11.7)
OR						
Making sure that no one votes who is not eligible to vote	1255	23.9 (22.3,25.5)	1264	89.4 (86.7,92.2)	601	89.7 (85.5,93.8)
aPD (95% CI; q-value)	Referent		-57.7 (-61.1,-54.2; <0.001)		-60.0 (-63.9,-56.0; <0.001)	
When you think about [the other political party], do you think about them as…						
Political opposition – that is, if they win, you just won't get the policies you want	2135	59.2 (57.0,61.3)	548	43.6 (40.0,47.2)	368	59.5 (54.2,64.7)
Enemies--that is, if they win, your life or your entire way of life may be threatened	1459	40.0 (37.8,42.1)	789	55.9 (52.2,59.5)	273	39.6 (34.4,44.9)
aPD (95% CI; q-value)	Referent		15.5 (11.0,19.9; <0.001)		0.4 (-5.3,6.1; 0.98)	
The 2020 election was stolen from Donald Trump, and Joe Biden is an illegitimate president.						
Do not agree	4353	88.4 (86.9,89.8)	123	8.3 (6.3,10.2)	169	25.8 (21.3,30.3)
Somewhat agree	189	6.0 (4.9,7.1)	394	28.4 (25.1,31.7)	262	37.9 (32.8,43.0)
Strongly or very strongly agree	151	4.7 (3.7,5.6)	822	62.8 (59.3,66.3)	209	35.8 (30.4,41.1)
aPD (95% CI; q-value)	Referent		57.8 (54.0,61.6; <0.001)		31.9 (26.5,37.2; <0.001)	
Armed citizens should patrol polling places at election time.						
Do not agree	4383	88.6 (87.2,90.0)	929	65.5 (62.0,69.0)	540	81.9 (77.5,86.4)
Somewhat agree	203	6.4 (5.3,7.5)	208	15.7 (13.2,18.2)	74	10.7 (7.7,13.6)
Strongly or very strongly agree	109	3.9 (3.0,4.7)	194	17.4 (14.3,20.4)	29	6.3 (2.9,9.7)
aPD (95% CI; q-value)	Referent		15.3 (12.0,18.6; <0.001)		4.6 (1.2,8.0; 0.04)	

Table S9, continued.

Statement or Query	MAGA Supporter, Non-Republican		Non-MAGA, Republican		Unclassified	
	Unweighted n	Weighted % (95% CI)	Unweighted n	Weighted % (95% CI)	Unweighted n	Weighted % (95% CI)
Which is more important to you?						
Having election outcomes determined democratically	105	37.7 (29.0,46.4)	1040	55.8 (52.6,59.0)	79	23.8 (17.5,30.1)
OR						
Having political leaders I can trust to look out for my values and interests	104	56.5 (47.6,65.5)	635	39.4 (36.2,42.5)	95	46.9 (38.5,55.4)
aPD (95% CI; q-value)	-26.5 (-35.1,-17.9; <0.001)		-13.9 (-17.6,-10.1; <0.001)		-28.3 (-37.2,-19.4; <0.001)	
Which is more important to you?						
Allowing people the personal freedom to do as they please	79	39.3 (30.1,48.5)	451	27.2 (24.4,30.1)	28	13.9 (7.5,20.3)
OR						
Having people work together for a better country	133	54.0 (44.7,63.3)	1231	69.6 (66.6,72.5)	156	60.3 (51.8,68.9)
aPD (95% CI; q-value)	-21.8 (-30.7,-12.9; <0.001)		-11.5 (-14.9,-8.2; <0.001)		-4.8 (-13.6,4.0; 0.53)	
Which is more important to you?						
Making sure that everyone who wants to vote can do so	58	36.1 (27.1,45.2)	419	29.4 (26.3,32.5)	70	34.8 (26.5,43.1)
OR						
Making sure that no one votes who is not eligible to vote	153	55.1 (45.7,64.5)	1273	67.7 (64.5,70.9)	115	41.5 (33.3,49.6)
aPD (95% CI; q-value)	-34.0 (-42.8,-25.2; <0.001)		-40.6 (-44.3,-37.0; <0.001)		-25.6 (-35.4,-15.7; <0.001)	
When you think about [the other political party], do you think about them as…						
Political opposition – that is, if they win, you just won't get the policies you want	65	66.8 (52.1,81.6)	1332	77.6 (75.0,80.3)	63	49.2 (37.2,61.1)
Enemies--that is, if they win, your life or your entire way of life may be threatened	15	30.8 (16.1,45.6)	363	20.0 (17.5,22.5)	42	39.5 (27.6,51.3)
aPD (95% CI; q-value)	-11.6 (-27.1,3.9; 0.35)		-18.2 (-21.7,-14.7; <0.001)		2.2 (-10.7,15.0; 0.98)	
The 2020 election was stolen from Donald Trump, and Joe Biden is an illegitimate president.						
Do not agree	72	40.3 (31.0,49.6)	1027	55.9 (52.7,59.1)	99	36.5 (28.7,44.3)
Somewhat agree	64	22.7 (15.3,30.1)	397	23.8 (21.1,26.5)	32	16.4 (9.6,23.2)
Strongly or very strongly agree	84	35.9 (27.3,44.5)	280	19.2 (16.6,21.8)	34	15.7 (9.5,21.9)
aPD (95% CI; q-value)	29.5 (20.5,38.5; <0.001)		15.1 (12.3,17.8; <0.001)		17.2 (8.1,26.3; <0.001)	
Armed citizens should patrol polling places at election time.						
Do not agree	158	60.5 (51.0,69.9)	1513	85.7 (83.4,88.0)	175	69.5 (61.3,77.6)
Somewhat agree	27	18.8 (10.6,27.0)	130	8.9 (7.0,10.7)	18	11.2 (4.9,17.4)
Strongly or very strongly agree	32	17.8 (10.3,25.4)	66	4.8 (3.4,6.2)	10	5.6 (1.6,9.5)
aPD (95% CI; q-value)	10.8 (3.5,18.1; 0.02)		2.4 (0.7,4.1; 0.03)		0.7 (-4.2,5.5; 0.88)	

Adjusted prevalence (aPDs) are absolute percentage point (pp) differences for the following responses: item 1, “Having election outcomes determined democratically”; item 2, “Having people work together for a better country”; item 3, “Making sure that everyone who wants to vote can do so”; item 4, “Enemies--that is, if they win, your life or your entire way of life may be threatened”; items 5-6, “strongly or very strongly agree.” They are adjusted for age, race and ethnicity, gender, education, income, Census division, rurality, military history, number of non-traffic arrests, and total number of drinks per week. Q-values, also known as FDR-adjusted (or FDR-corrected) p-values, represent the probability that the given difference would be a false discovery; they represent the expected proportion of “false positives” that would be seen among the collection of all differences whose q-values were at or below the given q-value. Item non-responses are not reported in the tables but are included in the prevalence calculations.

Table S10. MAGA affiliation and specified forms of hatred, fear, and enmity toward others

Condition	Non-MAGA, Non-Republican		MAGA Republican		MAGA Supporter, Republican	
	Unweighted n	Weighted % (95% CI)	Unweighted n	Weighted % (95% CI)	Unweighted n	Weighted % (95% CI)
Homonegativity						
Non-agreement	1110	22.2 (20.7,23.8)	2	0.2 (-0.2,0.6)	3	0.8 (-0.7,2.3)
Weak agreement	2116	44.4 (42.4,46.3)	73	9.1 (6.5,11.6)	49	10.8 (6.9,14.7)
Moderate agreement	936	19.6 (18.0,21.2)	282	20.8 (17.8,23.7)	189	29.4 (24.5,34.3)
Strong agreement	568	13.8 (12.4,15.2)	989	69.9 (66.5,73.4)	405	59.1 (53.7,64.4)
aPD (95% CI; q-value)	Referent		55.8 (51.8,59.8; <0.001)		46.0 (40.5,51.5; <0.001)	
Racism						
Non-agreement	1490	31.2 (29.4,32.9)	3	0.4 (-0.1,1.0)	2	0.2 (-1.1,1.5)
Weak agreement	1720	35.1 (33.3,37.0)	23	2.9 (1.3,4.4)	18	3.2 (1.0,5.4)
Moderate agreement	1243	28.2 (26.4,30.0)	374	32.7 (29.1,36.4)	257	39.2 (33.9,44.5)
Strong agreement	278	5.5 (4.6,6.4)	946	64.0 (60.3,67.7)	370	57.3 (52.1,62.6)
aPD (95% CI; q-value)	Referent		52.7 (48.7,56.7; <0.001)		47.3 (41.9,52.7; <0.001)	
Transphobia						
Non-agreement	833	19.8 (18.2,21.4)	7	1.5 (0.0,2.9)	4	0.6 (0.0,1.2)
Weak agreement	2436	46.7 (44.8,48.7)	138	12.1 (9.7,14.5)	110	16.5 (12.2,20.8)
Moderate agreement	1069	23.3 (21.6,25.0)	575	42.0 (38.4,45.5)	317	48.3 (42.7,54.0)
Strong agreement	393	10.2 (8.9,11.4)	626	44.5 (40.9,48.1)	215	34.6 (29.4,39.7)
aPD (95% CI; q-value)	Referent		35.0 (31.0,39.0; <0.001)		25.3 (20.0,30.6; <0.001)	
Xenophobia						
Non-agreement	1644	34.1 (32.3,36.0)	24	2.6 (1.1,4.1)	14	3.2 (0.7,5.7)
Weak agreement	2243	45.7 (43.8,47.6)	220	18.7 (15.7,21.7)	186	31.1 (25.7,36.5)
Moderate agreement	623	14.3 (12.9,15.7)	557	39.2 (35.7,42.8)	291	45.2 (39.8,50.7)
Strong agreement	221	5.9 (4.9,6.9)	546	39.5 (35.9,43.0)	154	20.5 (16.5,24.4)
aPD (95% CI; q-value)	Referent		31.9 (28.1,35.7; <0.001)		14.6 (10.3,18.9; <0.001)	

Table S10, continued.

Condition	Non-MAGA, Non-Republican		MAGA Republican		MAGA Supporter, Republican	
	Unweighted n	Weighted % (95% CI)	Unweighted n	Weighted % (95% CI)	Unweighted n	Weighted % (95% CI)
Hostile Sexism						
Non-agreement	2407	50.2 (48.3,52.1)	171	13.9 (11.3,16.5)	89	14.5 (10.7,18.3)
Weak agreement	1679	32.4 (30.7,34.2)	622	43.6 (40.0,47.2)	326	48.1 (42.7,53.5)
Moderate agreement	470	12.1 (10.7,13.5)	360	26.2 (23.1,29.4)	176	27.4 (22.6,32.2)
Strong agreement	175	5.3 (4.3,6.3)	193	16.3 (13.3,19.2)	55	10.1 (6.3,13.8)
aPD (95% CI; q-value)	Referent		12.8 (9.5,16.1; <0.001)		7.1 (3.4,10.8; 0.001)	
Islamophobia						
Non-agreement	2614	56.8 (54.9,58.7)	268	22.5 (19.3,25.7)	166	27.3 (22.4,32.1)
Weak agreement	1575	29.7 (27.9,31.4)	403	29.5 (26.1,32.8)	232	35.7 (30.6,40.9)
Moderate agreement	406	9.7 (8.5,11.0)	401	29.2 (25.8,32.6)	169	26.6 (21.6,31.6)
Strong agreement	136	3.8 (3.0,4.6)	273	18.8 (16.1,21.6)	80	10.4 (7.3,13.5)
aPD (95% CI; q-value)	Referent		14.8 (11.8,17.7; <0.001)		6.8 (3.4,10.2; 0.001)	
Antisemitism						
Non-agreement	2668	49.9 (48.0,51.9)	723	48.6 (45.0,52.3)	380	52.8 (47.2,58.3)
Weak agreement	1379	29.1 (27.3,30.9)	441	33.2 (29.8,36.7)	196	31.9 (26.7,37.0)
Moderate agreement	543	17.2 (15.6,18.9)	139	13.5 (10.6,16.5)	47	11.2 (6.8,15.6)
Strong agreement	142	3.7 (2.9,4.5)	44	4.6 (2.8,6.4)	23	4.2 (2.0,6.3)
aPD (95% CI; q-value)	Referent		2.2 (0.2,4.2; 0.10)		2.3 (-0.1,4.7; 0.14)	

Table S10, continued.

Condition	MAGA Supporter, Non-Republican		Non-MAGA, Republican		Unclassified	
	Unweighted n	Weighted % (95% CI)	Unweighted n	Weighted % (95% CI)	Unweighted n	Weighted % (95% CI)
Homonegativity						
Non-agreement	12	5.8 (1.9,9.6)	53	3.2 (2.1,4.4)	20	12.0 (3.8,20.1)
Weak agreement	39	24.1 (15.7,32.5)	341	21.8 (19.1,24.4)	71	34.2 (24.8,43.6)
Moderate agreement	60	28.5 (20.0,37.0)	554	33.0 (29.9,36.0)	60	27.4 (18.3,36.5)
Strong agreement	111	41.6 (32.6,50.6)	774	42.0 (38.9,45.2)	78	26.5 (18.7,34.3)
aPD (95% CI; q-value)	26.1 (16.9,35.3; <0.001)		29.7 (26.2,33.1; <0.001)		10.9 (2.6,19.2; 0.02)	
Racism						
Non-agreement	11	5.6 (1.6,9.7)	42	2.6 (1.6,3.6)	24	15.2 (6.7,23.6)
Weak agreement	19	8.5 (3.9,13.1)	255	16.3 (13.8,18.8)	54	26.0 (18.0,34.0)
Moderate agreement	92	48.7 (39.4,58.0)	891	52.0 (48.8,55.2)	91	35.4 (26.9,43.9)
Strong agreement	100	37.2 (28.4,46.0)	533	29.0 (26.2,31.9)	60	23.4 (16.1,30.7)
aPD (95% CI; q-value)	31.6 (22.7,40.4; <0.001)		20.9 (17.9,23.8; <0.001)		18.5 (11.2,25.9; <0.001)	
Transphobia						
Non-agreement	18	13.2 (6.5,19.9)	47	3.8 (2.3,5.3)	17	11.4 (4.3,18.5)
Weak agreement	44	19.4 (12.7,26.2)	474	26.1 (23.3,28.8)	96	42.9 (32.6,53.2)
Moderate agreement	98	39.1 (30.0,48.2)	791	44.2 (41.0,47.5)	75	31.3 (20.8,41.9)
Strong agreement	62	28.2 (20.1,36.4)	410	25.9 (23.1,28.7)	41	14.4 (8.6,20.2)
aPD (95% CI; q-value)	17.5 (8.8,26.2; <0.001)		17.1 (13.9,20.2; <0.001)		3.4 (-3.3,10.0; 0.56)	
Xenophobia						
Non-agreement	22	11.9 (6.2,17.7)	135	8.9 (7.0,10.7)	44	23.9 (12.6,35.2)
Weak agreement	45	22.2 (14.1,30.3)	706	42.2 (38.9,45.5)	92	42.7 (30.7,54.7)
Moderate agreement	84	33.8 (25.5,42.0)	588	32.1 (29.1,35.0)	63	22.5 (14.4,30.5)
Strong agreement	71	32.1 (23.1,41.1)	293	16.8 (14.5,19.2)	30	11.0 (6.1,15.8)
aPD (95% CI; q-value)	25.1 (16.0,34.2; <0.001)		11.4 (8.8,14.0; <0.001)		1.9 (-3.2,7.1; 0.77)	

Table S10, continued.

Condition	MAGA Supporter, Non-Republican		Non-MAGA, Republican		Unclassified	
	Unweighted n	Weighted % (95% CI)	Unweighted n	Weighted % (95% CI)	Unweighted n	Weighted % (95% CI)
Hostile Sexism						
Non-agreement	50	19.4 (12.6,26.1)	385	23.4 (20.7,26.1)	81	36.2 (27.5,44.9)
Weak agreement	75	32.8 (23.9,41.7)	882	48.3 (45.1,51.5)	103	40.9 (31.6,50.3)
Moderate agreement	59	26.2 (18.3,34.2)	350	20.8 (18.1,23.5)	34	18.1 (11.1,25.1)
Strong agreement	38	21.6 (13.6,29.5)	105	7.6 (5.8,9.4)	11	4.8 (1.1,8.4)
aPD (95% CI; q-value)	15.5 (7.2,23.7; 0.001)		4.5 (2.4,6.7; <0.001)		-1.2 (-5.4,3.1; 0.75)	
Islamophobia						
Non-agreement	63	33.5 (24.7,42.3)	598	38.1 (34.8,41.3)	111	54.7 (45.8,63.5)
Weak agreement	74	22.5 (15.8,29.2)	649	35.1 (31.9,38.3)	79	29.3 (21.2,37.4)
Moderate agreement	47	24.6 (16.1,33.0)	336	18.6 (16.1,21.1)	27	11.6 (5.9,17.3)
Strong agreement	38	19.4 (11.9,26.9)	138	8.2 (6.5,10.0)	12	4.3 (1.3,7.3)
aPD (95% CI; q-value)	14.0 (6.3,21.6; 0.003)		5.1 (3.1,7.1; <0.001)		-0.8 (-4.3,2.7; 0.76)	
Antisemitism						
Non-agreement	94	26.7 (19.8,33.7)	875	46.6 (43.4,49.8)	120	48.8 (37.3,60.3)
Weak agreement	60	29.1 (20.2,37.9)	601	33.6 (30.6,36.6)	75	32.8 (19.6,46.0)
Moderate agreement	49	30.1 (21.6,38.6)	187	15.7 (13.0,18.3)	29	15.4 (6.9,23.9)
Strong agreement	19	14.1 (6.7,21.5)	60	4.2 (2.9,5.5)	5	3.0 (-0.4,6.4)
aPD (95% CI; q-value)	10.8 (3.3,18.3; 0.06)		2.0 (0.4,3.6; 0.07)		-1.4 (-5.3,2.5; 0.66)	

Adjusted prevalence differences (aPDs) are absolute percentage point (pp) differences for the “strong agreement” category and are adjusted for age, race and ethnicity, gender, education, income, Census division, rurality, military history, number of non-traffic arrests, and total number of drinks per week. Q-values, also known as FDR-adjusted (or FDR-corrected) p-values, represent the probability that the given difference would be a false discovery; they represent the expected proportion of “false positives” that would be seen among the collection of all differences whose q-values were at or below the given q-value.

Table S11. MAGA affiliation and endorsement of QAnon and Christian nationalism

Statement	Non-MAGA, Non-Republican		MAGA Republican		MAGA Supporter, Republican	
	Unweighted n	Weighted % (95% CI)	Unweighted n	Weighted % (95% CI)	Unweighted n	Weighted % (95% CI)
The government, media, and financial worlds in the U.S. are controlled by a group of Satan-worshipping pedophiles who run a global child sex trafficking operation.						
Do not agree	4102	80.2 (78.4,81.9)	731	48.2 (44.6,51.8)	458	66.7 (61.5,71.9)
Somewhat agree	377	11.4 (10.0,12.8)	349	30.4 (26.8,33.9)	122	21.5 (16.9,26.2)
Strongly or very strongly agree	199	6.8 (5.6,7.9)	250	20.1 (17.1,23.2)	56	9.0 (6.2,11.8)
aPD (95% CI; q-value)	Referent		15.3 (12.0,18.6; <0.001)		5.7 (2.5,9.0; 0.003)	
There is a storm coming soon that will sweep away the elites in power and restore the rightful leaders.						
Do not agree	3884	76.7 (74.9,78.4)	540	40.4 (36.8,44.0)	405	58.7 (53.3,64.1)
Somewhat agree	586	15.4 (13.9,16.9)	501	34.8 (31.4,38.1)	192	31.3 (26.2,36.5)
Strongly or very strongly agree	207	6.3 (5.3,7.4)	282	22.5 (19.3,25.7)	41	7.3 (4.4,10.2)
aPD (95% CI; q-value)	Referent		17.6 (14.3,21.0; <0.001)		3.4 (0.1,6.7; 0.15)	
The chaos in America today is evidence that we are living in what the Bible calls 'the end times.'						
Do not agree	3464	67.3 (65.4,69.2)	465	31.6 (28.3,34.9)	346	52.5 (47.1,57.9)
Somewhat agree	743	17.5 (16.0,19.1)	496	38.1 (34.6,41.6)	181	27.6 (22.5,32.7)
Strongly or very strongly agree	487	14.0 (12.6,15.5)	374	29.2 (25.9,32.6)	110	17.2 (13.4,21.1)
aPD (95% CI; q-value)	Referent		19.7 (15.9,23.4; <0.001)		9.8 (5.4,14.3; <0.001)	
The U.S. government should declare America a Christian nation.						
Do not agree	3913	79.0 (77.3,80.6)	438	32.0 (28.6,35.5)	322	52.0 (46.6,57.4)
Somewhat agree	405	9.9 (8.7,11.1)	290	21.8 (18.8,24.8)	158	22.0 (17.6,26.4)
Strongly or very strongly agree	338	9.0 (7.8,10.2)	604	44.8 (41.3,48.4)	154	24.2 (19.5,28.9)
aPD (95% CI; q-value)	Referent		35.6 (31.7,39.5; <0.001)		16.8 (11.8,21.7; <0.001)	

Table S11, continued.

Statement	Non-MAGA, Non-Republican		MAGA Republican		MAGA Supporter, Republican	
	Unweighted n	Weighted % (95% CI)	Unweighted n	Weighted % (95% CI)	Unweighted n	Weighted % (95% CI)
U.S. laws should be based on Christian values.						
Do not agree	3299	68.6 (66.8,70.4)	169	15.9 (13.1,18.8)	125	23.0 (18.5,27.5)
Somewhat agree	870	17.7 (16.2,19.2)	381	27.8 (24.6,31.0)	238	35.6 (30.4,40.8)
Strongly or very strongly agree	500	12.0 (10.7,13.3)	783	54.9 (51.3,58.5)	275	39.7 (34.5,44.9)
aPD (95% CI; q-value)	Referent		44.2 (40.2,48.2; <0.001)		29.6 (24.4,34.9; <0.001)	
If the U.S. moves away from our Christian foundations, we will not have a country anymore.						
Do not agree	3561	71.9 (70.1,73.7)	160	14.5 (11.8,17.3)	132	23.8 (19.1,28.5)
Somewhat agree	637	14.8 (13.4,16.3)	366	26.5 (23.3,29.7)	238	32.2 (27.5,36.9)
Strongly or very strongly agree	474	11.6 (10.3,12.9)	806	57.7 (54.0,61.3)	270	43.5 (38.1,48.8)
aPD (95% CI; q-value)	Referent		45.9 (42.1,49.8; <0.001)		33.1 (27.6,38.6; <0.001)	
Being Christian is an important part of being truly American.						
Do not agree	3696	75.3 (73.5,77.0)	380	28.4 (25.1,31.7)	304	49.2 (43.9,54.6)
Somewhat agree	527	12.1 (10.8,13.4)	323	22.9 (19.9,25.9)	162	25.1 (20.2,30.1)
Strongly or very strongly agree	447	10.9 (9.6,12.2)	628	46.8 (43.1,50.4)	174	24.2 (19.9,28.4)
aPD (95% CI; q-value)	Referent		35.9 (32.0,39.8; <0.001)		15.3 (10.6,20.0; <0.001)	
God has called Christians to exercise dominion over all areas of American society.						
Do not agree	4076	82.6 (81.1,84.2)	727	49.7 (46.1,53.4)	478	70.7 (65.3,76.0)
Somewhat agree	296	7.1 (6.0,8.1)	276	21.3 (18.3,24.4)	86	16.7 (11.8,21.6)
Strongly or very strongly agree	273	7.9 (6.7,9.1)	317	26.1 (22.8,29.4)	72	9.9 (7.0,12.8)
aPD (95% CI; q-value)	Referent		20.1 (16.5,23.7; <0.001)		5.0 (1.5,8.4; 0.03)	

Table S11, continued.

Statement	MAGA Supporter, Non-Republican		Non-MAGA, Republican		Unclassified	
	Unweighted n	Weighted % (95% CI)	Unweighted n	Weighted % (95% CI)	Unweighted n	Weighted % (95% CI)
The government, media, and financial worlds in the U.S. are controlled by a group of Satan-worshipping pedophiles who run a global child sex trafficking operation.						
Do not agree	123	50.1 (40.9,59.3)	1373	73.5 (70.5,76.5)	100	33.9 (26.3,41.4)
Somewhat agree	50	22.9 (15.0,30.8)	220	15.4 (13.0,17.9)	35	17.5 (10.9,24.1)
Strongly or very strongly agree	49	27.0 (18.6,35.3)	106	9.1 (6.9,11.2)	10	4.3 (1.3,7.4)
aPD (95% CI; q-value)	17.6 (9.5,25.6; <0.001)		5.0 (2.6,7.5; <0.001)		-0.0 (-6.0,5.9; 0.99)	
There is a storm coming soon that will sweep away the elites in power and restore the rightful leaders.						
Do not agree	110	50.3 (41.1,59.5)	1286	71.3 (68.3,74.2)	91	33.3 (25.7,41.0)
Somewhat agree	69	26.9 (19.2,34.6)	326	20.7 (18.0,23.4)	43	16.9 (11.0,22.7)
Strongly or very strongly agree	40	20.5 (12.3,28.8)	86	5.9 (4.2,7.6)	13	6.5 (2.1,10.9)
aPD (95% CI; q-value)	12.0 (4.1,19.9; 0.02)		2.0 (-0.1,4.0; 0.16)		4.2 (-3.6,12.0; 0.40)	
The chaos in America today is evidence that we are living in what the Bible calls 'the end times.'						
Do not agree	109	46.3 (37.2,55.4)	974	52.9 (49.7,56.1)	65	23.5 (16.9,30.1)
Somewhat agree	63	25.0 (17.2,32.9)	469	27.3 (24.5,30.2)	54	19.2 (13.0,25.4)
Strongly or very strongly agree	50	28.7 (19.8,37.6)	263	18.7 (16.0,21.3)	48	22.4 (15.2,29.6)
aPD (95% CI; q-value)	12.0 (3.1,20.9; 0.02)		10.0 (7.0,12.9; <0.001)		17.0 (7.6,26.4; 0.002)	
The U.S. government should declare America a Christian nation.						
Do not agree	110	52.2 (43.0,61.5)	1021	57.6 (54.4,60.7)	84	36.2 (28.2,44.2)
Somewhat agree	50	21.0 (13.4,28.6)	334	19.3 (16.7,21.8)	33	12.1 (6.9,17.3)
Strongly or very strongly agree	59	24.8 (16.7,32.9)	343	21.4 (18.7,24.1)	36	10.8 (6.5,15.0)
aPD (95% CI; q-value)	14.7 (6.3,23.1; 0.002)		14.1 (11.2,17.1; <0.001)		5.4 (-1.8,12.7; 0.22)	

Table S11, continued.

Statement	MAGA Supporter, Non-Republican		Non-MAGA, Republican		Unclassified	
	Unweighted n	Weighted % (95% CI)	Unweighted n	Weighted % (95% CI)	Unweighted n	Weighted % (95% CI)
U.S. laws should be based on Christian values.						
Do not agree	83	44.1 (34.9,53.2)	575	35.4 (32.4,38.5)	58	28.5 (20.8,36.1)
Somewhat agree	63	22.3 (15.4,29.3)	577	31.6 (28.7,34.5)	51	18.6 (12.4,24.7)
Strongly or very strongly agree	74	32.5 (23.6,41.5)	552	31.6 (28.6,34.5)	55	17.4 (11.6,23.2)
aPD (95% CI; q-value)	20.3 (11.0,29.5; <0.001)		21.8 (18.5,25.0; <0.001)		12.5 (3.7,21.3; 0.02)	
If the U.S. moves away from our Christian foundations, we will not have a country anymore.						
Do not agree	78	44.2 (35.0,53.4)	701	42.2 (39.0,45.3)	60	24.9 (17.8,32.0)
Somewhat agree	60	19.1 (11.8,26.3)	525	27.9 (25.2,30.7)	52	22.0 (15.0,28.9)
Strongly or very strongly agree	82	35.9 (27.1,44.6)	477	28.4 (25.5,31.3)	51	16.7 (11.1,22.3)
aPD (95% CI; q-value)	23.1 (14.0,32.2; <0.001)		18.7 (15.5,21.9; <0.001)		11.3 (2.6,20.0; 0.02)	
Being Christian is an important part of being truly American.						
Do not agree	104	48.2 (39.1,57.4)	899	50.8 (47.6,54.0)	73	31.6 (23.8,39.4)
Somewhat agree	52	25.3 (16.6,34.0)	400	22.9 (20.2,25.6)	44	18.5 (12.1,24.9)
Strongly or very strongly agree	64	25.3 (17.8,32.9)	403	24.7 (21.9,27.4)	43	13.3 (8.5,18.1)
aPD (95% CI; q-value)	13.0 (5.0,21.1; 0.005)		16.1 (13.0,19.2; <0.001)		5.4 (-2.1,13.0; 0.22)	
God has called Christians to exercise dominion over all areas of American society.						
Do not agree	144	60.9 (51.6,70.1)	1281	70.4 (67.4,73.5)	103	38.9 (30.9,46.9)
Somewhat agree	38	20.8 (13.0,28.5)	229	14.0 (11.8,16.2)	21	7.6 (3.3,11.9)
Strongly or very strongly agree	37	17.5 (9.9,25.0)	179	12.8 (10.5,15.2)	20	6.6 (2.8,10.3)
aPD (95% CI; q-value)	7.3 (-0.3,14.9; 0.16)		7.6 (4.9,10.3; <0.001)		1.8 (-5.5,9.0; 0.75)	

Adjusted prevalence differences (aPDs) are absolute percentage point (pp) differences for “strongly or very strongly agree” responses and are adjusted for age, race and ethnicity, gender, education, income, Census division, rurality, military history, number of non-traffic arrests, and total number of drinks per week. Q-values, also known as FDR-adjusted (or FDR-corrected) p-values, represent the probability that the given difference would be a false discovery; they represent the expected proportion of “false positives” that would be seen among the collection of all differences whose q-values were at or below the given q-value. Item non-responses are not reported in the tables but are included in the prevalence calculations.

Table S12. MAGA affiliation and conspiracism

There is often debate about whether the public is told the whole truth about various important issues. How much do you agree or disagree with the following statements?	Non-MAGA, Non-Republican		MAGA Republican		MAGA Supporter, Republican	
	Unweighted n	Weighted % (95% CI)	Unweighted n	Weighted % (95% CI)	Unweighted n	Weighted % (95% CI)
The government is involved in the murder of innocent citizens and/or well-known public figures and keeps this a secret.						
Do not agree	3037	62.6 (60.6,64.6)	407	30.5 (27.2,33.9)	286	47.1 (41.4,52.8)
Somewhat agree	885	24.3 (22.5,26.0)	456	36.1 (32.5,39.7)	198	31.7 (26.7,36.7)
Strongly or very strongly agree	358	11.0 (9.6,12.4)	363	30.9 (27.3,34.5)	103	19.3 (14.6,23.9)
aPD (95% CI; q-value)	Referent		24.5 (20.5,28.4; <0.001)		12.7 (8.0,17.5; <0.001)	
The spread of certain viruses and/or diseases is the result of the deliberate, concealed efforts of some organization.						
Do not agree	3188	66.3 (64.3,68.3)	266	20.0 (17.1,23.0)	226	38.5 (33.1,44.0)
Somewhat agree	800	22.5 (20.7,24.3)	489	38.5 (34.9,42.2)	237	39.7 (34.1,45.4)
Strongly or very strongly agree	312	9.6 (8.3,10.9)	478	40.2 (36.4,43.9)	122	19.8 (15.7,23.9)
aPD (95% CI; q-value)	Referent		33.2 (29.2,37.3; <0.001)		14.1 (9.5,18.6; <0.001)	
The government permits or perpetrates acts of terrorism on its own soil, disguising its involvement.						
Do not agree	2916	59.6 (57.6,61.6)	271	23.1 (19.9,26.3)	208	34.6 (29.3,39.9)
Somewhat agree	1027	28.2 (26.3,30.0)	535	42.5 (38.7,46.2)	242	40.3 (34.7,45.9)
Strongly or very strongly agree	350	10.6 (9.2,11.9)	428	33.3 (29.8,36.8)	137	23.5 (18.8,28.1)
aPD (95% CI; q-value)	Referent		25.8 (21.8,29.8; <0.001)		17.1 (12.1,22.1; <0.001)	
Technology with mind-control capacities is used on people without their knowledge.						
Do not agree	3206	68.9 (67.0,70.8)	553	42.7 (39.0,46.3)	355	58.7 (53.0,64.4)
Somewhat agree	818	21.5 (19.7,23.2)	419	31.3 (27.9,34.8)	183	29.7 (24.3,35.2)
Strongly or very strongly agree	274	8.0 (6.8,9.2)	257	24.5 (21.0,28.1)	47	9.3 (6.0,12.5)
aPD (95% CI; q-value)	Referent		19.7 (15.8,23.6; <0.001)		5.4 (1.6,9.2; 0.02)	
The government falsely blames innocent people to hide its involvement in criminal activity.						
Do not agree	2644	52.7 (50.7,54.7)	168	14.9 (12.2,17.6)	121	20.0 (15.6,24.5)
Somewhat agree	1214	32.4 (30.5,34.4)	476	38.3 (34.6,42.0)	279	44.9 (39.4,50.5)
Strongly or very strongly agree	433	13.0 (11.5,14.5)	591	45.7 (41.9,49.4)	187	33.1 (27.5,38.7)
aPD (95% CI; q-value)	Referent		35.9 (31.7,40.1; <0.001)		24.5 (18.8,30.1; <0.001)	
Experiments involving new drugs or technologies are routinely carried out on the public without their knowledge or consent.						
Do not agree	2939	60.6 (58.5,62.6)	405	30.5 (27.2,33.9)	268	41.1 (35.7,46.5)
Somewhat agree	995	27.0 (25.1,28.9)	486	38.6 (34.9,42.3)	236	41.2 (35.5,46.9)
Strongly or very strongly agree	358	10.7 (9.3,12.0)	340	29.3 (25.7,32.8)	81	15.4 (11.3,19.5)
aPD (95% CI; q-value)	Referent		22.7 (18.8,26.6; <0.001)		10.0 (5.4,14.5; <0.001)	

Table S12, continued.

There is often debate about whether the public is told the whole truth about various important issues. How much do you agree or disagree with the following statements?	MAGA Supporter, Non-Republican		Non-MAGA, Republican		Unclassified	
	Unweighted n	Weighted % (95% CI)	Unweighted n	Weighted % (95% CI)	Unweighted n	Weighted % (95% CI)
The government is involved in the murder of innocent citizens and/or well-known public figures and keeps this a secret.						
Do not agree	76	32.7 (24.2,41.2)	961	56.7 (53.4,60.0)	65	26.6 (19.0,34.3)
Somewhat agree	60	26.9 (18.5,35.2)	440	29.9 (26.9,32.9)	50	26.6 (18.1,35.1)
Strongly or very strongly agree	69	36.4 (27.0,45.8)	164	12.1 (9.8,14.3)	24	10.9 (5.7,16.2)
aPD (95% CI; q-value)	24.3 (15.1,33.6; <0.001)		4.7 (2.1,7.4; 0.003)		4.8 (-3.3,13.0; 0.41)	
The spread of certain viruses and/or diseases is the result of the deliberate, concealed efforts of some organization.						
Do not agree	63	29.5 (21.0,38.0)	801	47.5 (44.2,50.8)	55	21.0 (14.2,27.8)
Somewhat agree	71	32.9 (24.0,41.8)	535	33.5 (30.4,36.6)	62	30.4 (21.8,39.1)
Strongly or very strongly agree	70	33.5 (24.6,42.4)	228	16.9 (14.3,19.5)	28	14.7 (8.4,20.9)
aPD (95% CI; q-value)	22.2 (12.4,31.9; <0.001)		10.9 (8.0,13.8; <0.001)		9.5 (0.2,18.7; 0.11)	
The government permits or perpetrates acts of terrorism on its own soil, disguising its involvement.						
Do not agree	60	30.9 (22.1,39.6)	857	51.3 (48.0,54.6)	59	21.6 (14.7,28.5)
Somewhat agree	73	32.4 (23.6,41.3)	508	33.5 (30.3,36.6)	54	28.5 (20.0,36.9)
Strongly or very strongly agree	72	32.2 (23.4,41.0)	196	13.5 (11.2,15.9)	27	12.3 (6.5,18.1)
aPD (95% CI; q-value)	20.9 (11.5,30.4; <0.001)		6.3 (3.6,9.0; <0.001)		8.3 (-0.8,17.4; 0.17)	
Technology with mind-control capacities is used on people without their knowledge.						
Do not agree	92	37.1 (28.3,46.0)	1013	61.3 (58.1,64.6)	71	28.3 (20.6,36.1)
Somewhat agree	63	30.6 (21.6,39.5)	410	26.6 (23.7,29.6)	59	28.3 (20.0,36.7)
Strongly or very strongly agree	51	28.7 (19.9,37.5)	139	10.6 (8.4,12.8)	20	11.0 (5.2,16.8)
aPD (95% CI; q-value)	21.0 (12.2,29.9; <0.001)		5.8 (3.3,8.3; <0.001)		6.2 (-2.3,14.8; 0.29)	
The government falsely blames innocent people to hide its involvement in criminal activity.						
Do not agree	47	24.9 (16.7,33.1)	702	42.6 (39.3,45.8)	57	21.3 (14.6,27.9)
Somewhat agree	77	33.1 (24.5,41.7)	613	38.8 (35.6,42.0)	61	31.2 (22.5,39.8)
Strongly or very strongly agree	81	38.0 (28.6,47.3)	253	17.4 (14.9,20.0)	27	12.3 (6.5,18.2)
aPD (95% CI; q-value)	25.2 (15.5,34.9; <0.001)		7.4 (4.5,10.4; <0.001)		4.2 (-5.0,13.3; 0.55)	
Experiments involving new drugs or technologies are routinely carried out on the public without their knowledge or consent.						
Do not agree	75	37.4 (28.1,46.8)	890	51.7 (48.4,55.0)	62	23.5 (16.3,30.7)
Somewhat agree	74	31.1 (22.9,39.4)	477	31.4 (28.3,34.5)	60	28.3 (20.1,36.5)
Strongly or very strongly agree	56	27.8 (19.2,36.4)	197	15.7 (13.1,18.3)	26	15.8 (8.8,22.8)
aPD (95% CI; q-value)	15.7 (6.5,25.0; 0.003)		9.1 (6.2,12.0; <0.001)		11.1 (1.0,21.2; 0.07)	

Adjusted prevalence differences (aPDs) are absolute percentage point (pp) differences for “strongly or very strongly agree” responses and are adjusted for age, race and ethnicity, gender, education, income, Census division, rurality, military history, number of non-traffic arrests, and total number of drinks per week. Q-values, also known as FDR-adjusted (or FDR-corrected) p-values, represent the probability that the given difference would be a false discovery; they represent the expected proportion of “false positives” that would be seen among the collection of all differences whose q-values were at or below the given q-value. Item non-responses are not reported in the tables but are included in the prevalence calculations.

Table S13. MAGA affiliation and trait aggression

Sometimes disagreements and conflicts occur in our lives. The next items are about ways you might respond to some of those things. How much do you agree or disagree with each of the following statements?	Non-MAGA, Non-Republican		MAGA Republican		MAGA Supporter, Republican	
	Unweighted n	Weighted % (95% CI)	Unweighted n	Weighted % (95% CI)	Unweighted n	Weighted % (95% CI)
Given enough provocation, I may hit another person.						
Do not agree	2896	66.2 (64.3,68.2)	728	59.5 (55.8,63.3)	356	60.3 (54.7,65.9)
Somewhat agree	1097	23.5 (21.8,25.2)	347	27.2 (23.7,30.7)	177	28.1 (23.0,33.3)
Strongly or very strongly agree	315	8.7 (7.5,10.0)	159	12.2 (9.9,14.6)	55	10.2 (6.9,13.5)
aPD (95% CI; q-value)	Referent		5.7 (2.9,8.5; <0.001)		2.8 (-0.9,6.4; 0.49)	
There are people who pushed me so far that we came to blows.						
Do not agree	3703	82.2 (80.5,83.8)	1006	76.7 (73.1,80.2)	510	82.6 (78.1,87.2)
Somewhat agree	414	10.3 (9.0,11.6)	152	14.7 (11.6,17.8)	60	11.7 (8.1,15.4)
Strongly or very strongly agree	188	5.8 (4.7,6.9)	76	7.6 (5.4,9.8)	18	3.1 (1.3,4.8)
aPD (95% CI; q-value)	Referent		3.4 (0.9,5.8; 0.03)		-1.0 (-3.1,1.2; 0.45)	
I have threatened people I know.						
Do not agree	3993	88.7 (87.2,90.1)	1135	86.5 (83.2,89.7)	555	90.1 (86.0,94.2)
Somewhat agree	231	6.7 (5.6,7.8)	67	7.8 (5.2,10.3)	30	7.2 (3.9,10.4)
Strongly or very strongly agree	83	3.1 (2.3,3.9)	33	4.8 (2.6,7.1)	4	0.6 (-0.1,1.3)
aPD (95% CI; q-value)	Referent		3.3 (0.8,5.8; 0.11)		-0.9 (-2.2,0.3; 0.36)	
At times I feel I have gotten a raw deal out of life.						
Do not agree	2991	63.1 (61.2,65.1)	876	64.7 (60.9,68.6)	426	66.9 (61.2,72.5)
Somewhat agree	1007	25.9 (24.1,27.7)	255	23.5 (20.0,27.0)	126	22.6 (18.0,27.3)
Strongly or very strongly agree	302	8.9 (7.7,10.1)	104	10.7 (8.0,13.3)	36	7.4 (3.8,11.0)
aPD (95% CI; q-value)	Referent		2.9 (-0.2,5.9; 0.19)		0.5 (-3.2,4.2; 0.86)	
Other people always seem to get the breaks.						
Do not agree	3053	63.2 (61.2,65.2)	832	60.2 (56.3,64.1)	433	64.5 (58.6,70.3)
Somewhat agree	1008	27.3 (25.4,29.1)	306	28.7 (25.1,32.3)	124	26.2 (21.0,31.4)
Strongly or very strongly agree	239	7.7 (6.5,8.9)	94	9.9 (7.2,12.5)	33	8.1 (3.8,12.3)
aPD (95% CI; q-value)	Referent		3.9 (0.7,7.0; 0.11)		2.9 (-1.3,7.0; 0.40)	
I wonder why sometimes I feel so bitter about things.						
Do not agree	2991	65.1 (63.2,67.1)	866	65.3 (61.5,69.1)	430	69.7 (64.2,75.1)
Somewhat agree	1073	26.0 (24.3,27.8)	282	24.4 (20.9,28.0)	134	23.3 (18.4,28.3)
Strongly or very strongly agree	241	7.1 (6.0,8.2)	86	8.9 (6.5,11.3)	24	4.7 (2.5,6.9)
aPD (95% CI; q-value)	Referent		2.7 (-0.1,5.6; 0.21)		-1.1 (-3.7,1.6; 0.80)	

Table S13, continued.

Sometimes disagreements and conflicts occur in our lives. The next items are about ways you might respond to some of those things. How much do you agree or disagree with each of the following statements?	MAGA Supporter, Non-Republican		Non-MAGA, Republican		Unclassified	
	Unweighted n	Weighted % (95% CI)	Unweighted n	Weighted % (95% CI)	Unweighted n	Weighted % (95% CI)
Given enough provocation, I may hit another person.						
Do not agree	122	60.1 (50.8,69.4)	1050	67.1 (63.9,70.2)	117	50.1 (41.0,59.3)
Somewhat agree	47	19.3 (11.8,26.8)	388	23.4 (20.6,26.2)	26	12.5 (6.4,18.5)
Strongly or very strongly agree	35	14.6 (8.2,20.9)	132	8.4 (6.5,10.2)	11	6.0 (1.5,10.6)
aPD (95% CI; q-value)	5.2 (-2.1,12.4; 0.49)		1.5 (-0.7,3.8; 0.49)		0.6 (-6.5,7.7; 0.94)	
There are people who pushed me so far that we came to blows.						
Do not agree	152	69.7 (60.6,78.7)	1341	84.6 (82.2,86.9)	130	51.9 (42.6,61.1)
Somewhat agree	26	10.7 (5.5,15.9)	153	9.0 (7.2,10.7)	22	13.7 (7.2,20.2)
Strongly or very strongly agree	27	15.3 (7.6,22.9)	73	5.1 (3.6,6.6)	6	3.7 (0.1,7.3)
aPD (95% CI; q-value)	8.0 (0.2,15.8; 0.11)		1.0 (-0.8,2.9; 0.36)		-0.5 (-6.0,5.1; 0.87)	
I have threatened people I know.						
Do not agree	169	75.3 (66.4,84.2)	1468	91.2 (89.1,93.3)	146	62.7 (53.4,72.0)
Somewhat agree	19	6.8 (3.0,10.7)	72	5.4 (3.7,7.0)	11	6.9 (2.1,11.7)
Strongly or very strongly agree	16	11.8 (4.4,19.3)	31	2.3 (1.2,3.4)	4	1.1 (-0.1,2.3)
aPD (95% CI; q-value)	8.5 (0.9,16.0; 0.18)		0.6 (-0.7,2.0; 0.54)		-2.3 (-4.4,-0.1; 0.20)	
At times I feel I have gotten a raw deal out of life.						
Do not agree	135	55.9 (46.4,65.4)	1122	68.4 (65.3,71.5)	98	36.8 (28.5,45.1)
Somewhat agree	41	19.8 (12.8,26.8)	335	21.7 (19.0,24.4)	43	20.7 (13.3,28.1)
Strongly or very strongly agree	30	20.4 (12.0,28.8)	112	8.8 (6.7,10.8)	17	10.9 (5.2,16.5)
aPD (95% CI; q-value)	9.7 (1.3,18.1; 0.09)		1.5 (-0.9,3.9; 0.43)		5.3 (-2.8,13.5; 0.43)	
Other people always seem to get the breaks.						
Do not agree	129	47.9 (38.5,57.2)	1087	65.2 (62.0,68.4)	99	38.7 (30.2,47.1)
Somewhat agree	48	25.0 (16.4,33.5)	385	25.7 (22.8,28.6)	40	17.9 (11.1,24.7)
Strongly or very strongly agree	28	21.6 (12.8,30.3)	92	7.7 (5.8,9.6)	19	12.6 (6.2,19.0)
aPD (95% CI; q-value)	12.9 (4.1,21.7; 0.03)		1.7 (-0.5,3.9; 0.37)		9.8 (1.0,18.5; 0.15)	
I wonder why sometimes I feel so bitter about things.						
Do not agree	139	56.3 (46.6,65.9)	1131	69.4 (66.3,72.4)	105	39.1 (30.5,47.6)
Somewhat agree	42	19.0 (11.4,26.5)	348	22.2 (19.5,24.9)	38	20.1 (12.9,27.3)
Strongly or very strongly agree	23	19.0 (10.5,27.4)	88	7.2 (5.3,9.1)	17	11.5 (5.4,17.6)
aPD (95% CI; q-value)	11.1 (2.5,19.7; 0.08)		0.9 (-1.4,3.2; 0.80)		8.5 (0.6,16.4; 0.18)	

Adjusted prevalence differences (aPDs) are absolute percentage point (pp) differences for “strongly or very strongly agree” responses and are adjusted for age, race and ethnicity, gender, education, income, Census division, rurality, military history, number of non-traffic arrests, and total number of drinks per week. Q-values, also known as FDR-adjusted (or FDR-corrected) p-values, represent the probability that the given difference would be a false discovery; they represent the expected proportion of “false positives” that would be seen among the collection of all differences whose q-values were at or below the given q-value. Item non-responses are not reported in the tables but are included in the prevalence calculations.

Table S14. MAGA affiliation and justification for violence in non-political circumstances

**What do you think about the use of force or violence in the following situations?**	**Non-MAGA, Non-Republican**		**MAGA Republican**		**MAGA Supporter, Republican**	
	**Unweighted n**	**Weighted % (95% CI)**	**Unweighted n**	**Weighted % (95% CI)**	**Unweighted n**	**Weighted % (95% CI)**
In self-defense						
Never justified	85	2.2 (1.6,2.8)	5	0.5 (0.0,1.0)	1	0.2 (-0.2,0.7)
Sometimes justified	1227	28.1 (26.3,29.9)	109	11.0 (8.4,13.7)	46	8.4 (5.1,11.7)
Usually or always justified	3398	68.9 (67.1,70.7)	1227	88.1 (85.3,90.8)	597	91.0 (87.7,94.4)
aPD (95% CI; q-value)	Referent		16.1 (12.8,19.4; <0.001)		18.0 (13.9,22.0; <0.001)	
To prevent someone from injuring or killing another person						
Never justified	88	2.9 (2.1,3.6)	6	0.5 (0.1,0.9)	1	0.2 (-0.2,0.7)
Sometimes justified	1046	24.9 (23.2,26.7)	83	8.9 (6.3,11.4)	46	9.9 (6.3,13.5)
Usually or always justified	3557	71.0 (69.2,72.8)	1246	90.0 (87.4,92.6)	596	89.5 (85.8,93.1)
aPD (95% CI; q-value)	Referent		14.2 (10.9,17.5; <0.001)		12.3 (8.1,16.6; <0.001)	
To prevent someone from injuring or killing themselves						
Never justified	311	7.6 (6.5,8.7)	59	4.8 (3.1,6.5)	22	2.5 (1.3,3.7)
Sometimes justified	1753	38.6 (36.8,40.5)	351	25.4 (22.3,28.5)	211	34.8 (29.5,40.2)
Usually or always justified	2639	52.9 (50.9,54.8)	930	69.4 (66.0,72.7)	409	61.3 (55.9,66.7)
aPD (95% CI; q-value)	Referent		13.7 (9.7,17.7; <0.001)		7.6 (2.0,13.3; 0.04)	
To prevent harm or damage to property						
Never justified	966	20.3 (18.8,21.8)	83	7.6 (5.6,9.6)	39	5.4 (3.3,7.4)
Sometimes justified	2526	52.4 (50.4,54.3)	513	37.8 (34.3,41.3)	307	47.7 (42.4,53.1)
Usually or always justified	1211	26.5 (24.8,28.2)	744	54.1 (50.5,57.7)	296	46.3 (40.9,51.7)
aPD (95% CI; q-value)	Referent		26.5 (22.3,30.6; <0.001)		21.0 (15.2,26.9; <0.001)	

Table S14, continued.

What do you think about the use of force or violence in the following situations?	Non-MAGA, Non-Republican		MAGA Republican		MAGA Supporter, Republican	
	Unweighted n	Weighted % (95% CI)	Unweighted n	Weighted % (95% CI)	Unweighted n	Weighted % (95% CI)
To win an argument						
Never justified	4264	86.6 (85.1,88.0)	1183	84.4 (81.6,87.3)	590	90.9 (87.7,94.1)
Sometimes justified	323	8.8 (7.6,10.0)	124	11.7 (9.2,14.3)	45	7.2 (4.4,10.0)
Usually or always justified	114	3.7 (2.8,4.5)	35	3.5 (2.0,5.1)	7	1.4 (-0.2,2.9)
aPD (95% CI; q-value)	Referent		1.1 (-0.7,2.9; 0.58)		-0.2 (-2.2,1.7; 1.00)	
In response to an insult						
Never justified	4094	81.6 (80.0,83.2)	1149	80.5 (77.2,83.7)	584	89.4 (86.0,92.9)
Sometimes justified	475	13.6 (12.1,15.1)	141	13.9 (11.1,16.8)	50	8.0 (5.0,10.9)
Usually or always justified	133	3.9 (3.1,4.7)	48	4.9 (3.0,6.8)	9	2.1 (0.3,3.9)
aPD (95% CI; q-value)	Referent		2.1 (-0.0,4.3; 0.25)		0.1 (-2.0,2.2; 0.99)	
To get respect						
Never justified	4286	86.9 (85.5,88.4)	1222	86.6 (83.7,89.5)	607	93.6 (90.7,96.5)
Sometimes justified	281	7.9 (6.7,9.0)	83	8.1 (5.8,10.3)	26	3.6 (1.4,5.7)
Usually or always justified	138	4.3 (3.4,5.2)	36	4.9 (2.9,7.0)	10	2.3 (0.3,4.3)
aPD (95% CI; q-value)	Referent		1.9 (-0.4,4.2; 0.52)		0.1 (-2.3,2.4; 0.96)	

Table S14, continued.

**What do you think about the use of force or violence in the following situations?**	**MAGA Supporter, Non-Republican**		**Non-MAGA, Republican**		**Unclassified**	
	**Unweighted n**	**Weighted % (95% CI)**	**Unweighted n**	**Weighted % (95% CI)**	**Unweighted n**	**Weighted % (95% CI)**
In self-defense						
Never justified	4	4.4 (-1.3,10.0)	15	2.0 (0.8,3.2)	5	1.3 (0.0,2.5)
Sometimes justified	38	19.1 (11.7,26.5)	236	16.4 (13.9,18.8)	50	25.7 (18.1,33.3)
Usually or always justified	179	75.0 (66.2,83.7)	1468	81.3 (78.6,84.0)	159	63.4 (55.1,71.6)
aPD (95% CI; q-value)	6.4 (-2.2,15.1; 0.32)		9.7 (6.4,13.0; <0.001)		1.1 (-7.6,9.7; 0.91)	
To prevent someone from injuring or killing another person						
Never justified	7	5.6 (-0.2,11.5)	8	1.0 (0.2,1.8)	6	1.9 (-0.0,3.9)
Sometimes justified	40	20.9 (13.3,28.5)	208	15.9 (13.4,18.4)	35	18.1 (11.5,24.7)
Usually or always justified	174	71.9 (63.0,80.8)	1499	82.6 (80.0,85.2)	170	68.7 (60.7,76.7)
aPD (95% CI; q-value)	2.1 (-6.3,10.5; 0.84)		7.4 (4.1,10.6; <0.001)		6.6 (-1.5,14.7; 0.23)	
To prevent someone from injuring or killing themselves						
Never justified	16	8.1 (2.1,14.1)	62	3.8 (2.5,5.0)	12	4.8 (1.2,8.4)
Sometimes justified	72	31.9 (23.4,40.4)	564	34.0 (30.9,37.1)	59	25.4 (18.0,32.7)
Usually or always justified	132	58.3 (49.1,67.5)	1089	61.8 (58.7,65.0)	137	58.2 (49.8,66.6)
aPD (95% CI; q-value)	8.2 (-0.8,17.2; 0.17)		7.1 (3.4,10.9; 0.002)		12.5 (3.5,21.5; 0.04)	
To prevent harm or damage to property						
Never justified	34	21.3 (13.1,29.5)	151	9.8 (7.8,11.8)	27	10.4 (5.8,14.9)
Sometimes justified	90	39.5 (30.4,48.6)	873	51.8 (48.6,55.0)	94	44.9 (36.4,53.4)
Usually or always justified	97	37.7 (29.2,46.1)	694	38.0 (34.9,41.0)	89	33.1 (25.3,40.8)
aPD (95% CI; q-value)	10.3 (1.7,19.0; 0.05)		12.5 (8.9,16.1; <0.001)		6.6 (-2.5,15.7; 0.29)	

Table S14, continued.

**What do you think about the use of force or violence in the following situations?**	**MAGA Supporter, Non-Republican**		**Non-MAGA, Republican**		**Unclassified**	
	**Unweighted n**	**Weighted % (95% CI)**	**Unweighted n**	**Weighted % (95% CI)**	**Unweighted n**	**Weighted % (95% CI)**
To win an argument						
Never justified	191	81.9 (74.1,89.6)	1556	87.6 (85.3,89.9)	170	68.1 (59.8,76.3)
Sometimes justified	19	10.3 (4.0,16.7)	127	8.5 (6.7,10.4)	33	16.1 (9.8,22.5)
Usually or always justified	11	6.3 (1.8,10.7)	34	3.4 (1.9,4.9)	9	4.9 (0.4,9.4)
aPD (95% CI; q-value)	0.4 (-3.9,4.7; 1.00)		1.4 (-0.4,3.2; 0.37)		0.7 (-4.8,6.2; 1.00)	
In response to an insult						
Never justified	179	74.4 (66.0,82.7)	1535	86.7 (84.4,89.1)	169	67.0 (58.7,75.3)
Sometimes justified	29	15.1 (8.9,21.2)	140	9.3 (7.3,11.3)	31	16.5 (10.0,23.0)
Usually or always justified	13	9.1 (2.7,15.4)	41	3.5 (2.1,4.8)	11	5.5 (0.9,10.1)
aPD (95% CI; q-value)	4.0 (-2.1,10.1; 0.54)		1.2 (-0.4,2.8; 0.47)		1.5 (-4.0,7.1; 0.76)	
To get respect						
Never justified	184	78.1 (70.1,86.0)	1582	89.7 (87.5,91.9)	177	71.0 (63.0,79.1)
Sometimes justified	25	11.7 (6.4,16.9)	95	6.5 (4.7,8.3)	26	12.6 (7.0,18.1)
Usually or always justified	11	8.2 (2.0,14.3)	39	3.3 (2.0,4.7)	7	5.3 (0.4,10.2)
aPD (95% CI; q-value)	2.4 (-3.7,8.4; 0.74)		0.9 (-0.8,2.6; 0.64)		0.4 (-5.6,6.4; 0.96)	

Adjusted prevalence differences (aPDs) are absolute percentage point (pp) differences for “usually or always justified” responses and are adjusted for age, race and ethnicity, gender, education, income, Census division, rurality, military history, number of non-traffic arrests, and total number of drinks per week. Q-values, also known as FDR-adjusted (or FDR-corrected) p-values, represent the probability that the given difference would be a false discovery; they represent the expected proportion of “false positives” that would be seen among the collection of all differences whose q-values were at or below the given q-value. Item non-responses are not reported in the tables but are included in the prevalence calculations.

Table S15. MAGA affiliation and justification for intimate partner violence

How much do you agree or disagree with the following reasons why it might be understandable that someone could hit a partner?	Non-MAGA, Non-Republican		MAGA Republican		MAGA Supporter, Republican	
	Unweighted n	Weighted % (95% CI)	Unweighted n	Weighted % (95% CI)	Unweighted n	Weighted % (95% CI)
A partner hurts your child, either physically or emotionally.						
Do not agree	2049	43.8 (41.9,45.7)	507	37.7 (34.2,41.3)	248	36.7 (31.7,41.7)
Somewhat agree	1381	27.0 (25.3,28.6)	322	22.2 (19.3,25.0)	188	27.3 (22.6,32.1)
Strongly or very strongly agree	1229	27.4 (25.7,29.1)	500	39.0 (35.5,42.6)	201	33.2 (28.0,38.3)
aPD (95% CI; q-value)	Referent		10.9 (6.8,15.1; <0.001)		6.1 (0.6,11.6; 0.10)	
A partner hits you first.						
Do not agree	2337	50.0 (48.0,51.9)	660	47.0 (43.4,50.6)	308	43.3 (38.1,48.5)
Somewhat agree	1484	28.8 (27.1,30.5)	367	26.0 (23.0,29.1)	190	29.7 (24.8,34.6)
Strongly or very strongly agree	840	19.5 (17.9,21.0)	301	25.8 (22.4,29.2)	137	23.7 (18.9,28.5)
aPD (95% CI; q-value)	Referent		8.9 (5.0,12.8; <0.001)		6.6 (1.5,11.7; 0.06)	
A partner cheats on you.						
Do not agree	4124	84.6 (83.1,86.1)	1111	82.1 (79.3,85.0)	557	81.3 (76.2,86.4)
Somewhat agree	364	9.0 (7.8,10.2)	136	9.3 (7.3,11.3)	65	12.4 (8.4,16.4)
Strongly or very strongly agree	181	4.7 (3.8,5.6)	85	7.6 (5.3,9.8)	16	3.6 (0.7,6.5)
aPD (95% CI; q-value)	Referent		3.9 (1.4,6.5; 0.03)		0.4 (-2.6,3.3; 0.89)	
A partner steals from you.						
Do not agree	4186	86.9 (85.6,88.3)	1109	82.2 (79.4,85.1)	563	84.9 (80.1,89.7)
Somewhat agree	315	7.0 (6.0,8.0)	129	8.7 (6.9,10.5)	64	11.2 (7.0,15.5)
Strongly or very strongly agree	168	4.4 (3.5,5.2)	89	8.0 (5.6,10.3)	11	1.1 (0.4,1.8)
aPD (95% CI; q-value)	Referent		4.7 (2.0,7.4; 0.01)		-2.0 (-3.1,-0.8; 0.02)	
A partner is drunk or using drugs.						
Do not agree	3893	81.7 (80.1,83.2)	1011	75.2 (72.1,78.3)	524	80.9 (76.6,85.2)
Somewhat agree	537	11.2 (10.0,12.4)	202	14.0 (11.7,16.3)	85	13.1 (9.7,16.6)
Strongly or very strongly agree	240	5.5 (4.6,6.4)	116	9.7 (7.3,12.1)	27	3.0 (1.3,4.7)
aPD (95% CI; q-value)	Referent		4.8 (2.0,7.7; 0.01)		-1.5 (-3.6,0.7; 0.51)	

Table S15, continued.

How much do you agree or disagree with the following reasons why it might be understandable that someone could hit a partner?	Non-MAGA, Non-Republican		MAGA Republican		MAGA Supporter, Republican	
	Unweighted n	Weighted % (95% CI)	Unweighted n	Weighted % (95% CI)	Unweighted n	Weighted % (95% CI)
A partner embarrassed or belittled you in front of others.						
Do not agree	4315	89.1 (87.8,90.4)	1165	86.9 (84.3,89.4)	600	89.3 (84.7,93.9)
Somewhat agree	233	5.8 (4.8,6.8)	109	7.5 (5.5,9.5)	32	5.3 (2.4,8.3)
Strongly or very strongly agree	126	3.5 (2.8,4.3)	58	4.8 (3.1,6.4)	8	2.8 (-0.1,5.7)
aPD (95% CI; q-value)	Referent		2.2 (0.2,4.2; 0.10)		0.8 (-2.2,3.8; 0.82)	
A partner continually nags you.						
Do not agree	4357	89.9 (88.6,91.1)	1206	89.8 (87.6,92.0)	611	93.4 (90.3,96.5)
Somewhat agree	216	5.5 (4.5,6.4)	81	5.6 (4.0,7.1)	22	3.2 (1.5,4.9)
Strongly or very strongly agree	100	3.0 (2.3,3.8)	44	3.6 (2.0,5.1)	5	0.6 (0.0,1.2)
aPD (95% CI; q-value)	Referent		1.7 (-0.1,3.5; 0.33)		-1.2 (-2.2,-0.1; 0.20)	
A partner threatened to hit you.						
Do not agree	3769	79.5 (77.9,81.0)	1020	75.3 (72.2,78.5)	521	78.1 (73.2,83.1)
Somewhat agree	621	12.4 (11.2,13.7)	192	13.0 (10.7,15.3)	90	14.8 (10.6,19.0)
Strongly or very strongly agree	273	6.4 (5.4,7.4)	116	10.5 (8.0,12.9)	27	4.1 (2.0,6.3)
aPD (95% CI; q-value)	Referent		4.9 (2.1,7.8; 0.01)		-0.9 (-3.4,1.6; 0.70)	
A partner tried to keep you from doing something.						
Do not agree	4051	83.9 (82.4,85.3)	1126	83.2 (80.4,86.0)	572	86.1 (81.4,90.8)
Somewhat agree	461	10.4 (9.2,11.6)	142	10.1 (8.0,12.1)	56	10.0 (5.9,14.1)
Strongly or very strongly agree	154	3.9 (3.1,4.7)	61	5.6 (3.6,7.6)	11	1.1 (0.4,1.9)
aPD (95% CI; q-value)	Referent		2.7 (0.3,5.1; 0.16)		-1.6 (-2.8,-0.3; 0.10)	
A partner forces you to have sex with him or her.						
Do not agree	1923	41.2 (39.3,43.1)	597	43.2 (39.6,46.8)	267	35.8 (30.9,40.7)
Somewhat agree	1089	20.4 (18.9,21.8)	274	20.2 (17.4,23.1)	151	25.0 (20.2,29.8)
Strongly or very strongly agree	1658	36.6 (34.8,38.5)	452	35.2 (31.7,38.6)	219	36.3 (31.1,41.6)
aPD (95% CI; q-value)	Referent		-0.3 (-4.3,3.7; 0.94)		-0.6 (-6.2,5.0; 0.94)	

Table S15, continued.

How much do you agree or disagree with the following reasons why it might be understandable that someone could hit a partner?	MAGA Supporter, Non-Republican		Non-MAGA, Republican		Unclassified	
	Unweighted n	Weighted % (95% CI)	Unweighted n	Weighted % (95% CI)	Unweighted n	Weighted % (95% CI)
A partner hurts your child, either physically or emotionally.						
Do not agree	95	40.7 (31.7,49.7)	724	41.7 (38.6,44.9)	87	38.8 (30.4,47.1)
Somewhat agree	47	18.7 (12.0,25.4)	495	27.3 (24.5,30.1)	36	13.8 (7.9,19.7)
Strongly or very strongly agree	77	39.9 (30.7,49.2)	479	28.9 (26.1,31.8)	47	15.9 (10.4,21.3)
aPD (95% CI; q-value)	11.0 (1.9,20.2; 0.08)		0.7 (-2.9,4.2; 0.77)		-6.1 (-14.4,2.2; 0.33)	
A partner hits you first.						
Do not agree	113	50.5 (41.3,59.7)	878	50.1 (46.9,53.3)	99	42.6 (34.2,51.0)
Somewhat agree	49	22.3 (14.4,30.3)	519	29.3 (26.5,32.1)	36	11.4 (6.6,16.2)
Strongly or very strongly agree	58	26.8 (18.8,34.8)	301	18.8 (16.3,21.3)	37	14.1 (8.5,19.7)
aPD (95% CI; q-value)	5.6 (-3.1,14.3; 0.33)		0.5 (-2.6,3.6; 0.89)		-0.4 (-8.7,8.0; 0.96)	
A partner cheats on you.						
Do not agree	169	74.2 (65.8,82.5)	1452	81.5 (78.8,84.1)	147	59.0 (50.6,67.5)
Somewhat agree	26	13.9 (6.9,21.0)	164	10.2 (8.2,12.3)	17	7.1 (2.9,11.3)
Strongly or very strongly agree	25	11.5 (5.8,17.2)	83	6.2 (4.5,7.8)	9	4.0 (0.4,7.6)
aPD (95% CI; q-value)	5.3 (-0.6,11.2; 0.19)		2.5 (0.6,4.5; 0.07)		-0.3 (-5.8,5.1; 0.93)	
A partner steals from you.						
Do not agree	168	74.6 (66.3,83.0)	1486	85.9 (83.6,88.2)	145	59.0 (50.6,67.4)
Somewhat agree	27	13.9 (7.3,20.4)	151	8.6 (6.7,10.4)	17	8.1 (3.2,13.0)
Strongly or very strongly agree	23	10.6 (4.3,16.9)	66	4.1 (2.9,5.4)	7	1.4 (0.2,2.7)
aPD (95% CI; q-value)	5.4 (-1.0,11.8; 0.21)		0.9 (-0.7,2.5; 0.41)		-3.4 (-5.7,-1.0; 0.03)	
A partner is drunk or using drugs.						
Do not agree	163	72.4 (63.8,81.0)	1345	77.1 (74.3,79.9)	147	58.7 (50.2,67.2)
Somewhat agree	29	16.8 (9.0,24.5)	245	14.3 (12.0,16.6)	19	8.4 (3.4,13.4)
Strongly or very strongly agree	27	10.1 (4.8,15.5)	113	6.9 (5.2,8.7)	6	2.1 (-0.5,4.7)
aPD (95% CI; q-value)	3.9 (-1.5,9.3; 0.48)		2.3 (0.2,4.4; 0.24)		-3.4 (-7.4,0.7; 0.37)	

Table S15, continued.

How much do you agree or disagree with the following reasons why it might be understandable that someone could hit a partner?	MAGA Supporter, Non-Republican		Non-MAGA, Republican		Unclassified	
	Unweighted n	Weighted % (95% CI)	Unweighted n	Weighted % (95% CI)	Unweighted n	Weighted % (95% CI)
A partner embarrassed or belittled you in front of others.						
Do not agree	176	75.4 (66.9,83.9)	1559	88.6 (86.4,90.8)	158	63.8 (55.5,72.1)
Somewhat agree	17	9.4 (3.9,15.0)	98	6.4 (4.7,8.1)	9	4.0 (0.5,7.6)
Strongly or very strongly agree	26	14.5 (7.1,21.9)	45	3.2 (2.1,4.3)	7	2.4 (-0.2,5.0)
aPD (95% CI; q-value)	9.4 (1.8,16.9; 0.09)		0.9 (-0.5,2.3; 0.42)		-1.3 (-5.3,2.7; 0.78)	
A partner continually nags you.						
Do not agree	188	81.5 (73.8,89.2)	1573	89.4 (87.2,91.5)	163	64.4 (56.1,72.7)
Somewhat agree	10	4.0 (0.8,7.2)	98	6.8 (5.1,8.6)	7	4.6 (0.7,8.4)
Strongly or very strongly agree	21	13.7 (6.5,21.0)	29	2.0 (1.1,2.9)	5	2.0 (-0.5,4.5)
aPD (95% CI; q-value)	9.3 (1.8,16.8; 0.20)		0.1 (-1.2,1.4; 0.99)		-0.6 (-4.4,3.2; 0.90)	
A partner threatened to hit you.						
Do not agree	155	67.2 (58.4,76.1)	1363	78.3 (75.6,81.0)	133	52.5 (44.0,61.0)
Somewhat agree	33	15.9 (9.3,22.5)	237	13.7 (11.4,15.9)	29	14.5 (7.9,21.0)
Strongly or very strongly agree	30	15.8 (8.3,23.3)	101	6.2 (4.7,7.7)	8	1.8 (0.2,3.4)
aPD (95% CI; q-value)	8.0 (0.0,15.9; 0.18)		0.9 (-1.0,2.8; 0.66)		-5.1 (-8.0,-2.3; 0.01)	
A partner tried to keep you from doing something.						
Do not agree	167	73.9 (65.7,82.1)	1512	85.5 (83.1,87.9)	152	61.7 (53.3,70.1)
Somewhat agree	28	13.7 (7.5,19.9)	140	9.3 (7.3,11.3)	15	7.0 (2.4,11.5)
Strongly or very strongly agree	24	11.7 (5.2,18.2)	46	3.2 (2.0,4.4)	3	0.5 (-0.1,1.1)
aPD (95% CI; q-value)	6.1 (-0.4,12.7; 0.20)		0.3 (-1.2,1.8; 0.83)		-4.0 (-5.5,-2.5; <0.001)	
A partner forces you to have sex with him or her.						
Do not agree	103	44.9 (35.8,54.0)	738	41.7 (38.6,44.9)	80	32.0 (24.2,39.9)
Somewhat agree	45	19.1 (11.7,26.6)	378	22.1 (19.4,24.8)	33	15.4 (9.2,21.6)
Strongly or very strongly agree	72	35.6 (26.8,44.5)	577	33.8 (30.8,36.8)	56	21.2 (14.7,27.8)
aPD (95% CI; q-value)	-1.4 (-10.4,7.7; 0.92)		-4.7 (-8.4,-1.1; 0.05)		-4.6 (-14.1,4.9; 0.60)	

Adjusted prevalence differences (aPDs) are absolute percentage point (pp) differences for “strongly or very strongly agree” responses and are adjusted for age, race and ethnicity, gender, education, income, Census division, rurality, military history, number of non-traffic arrests, and total number of drinks per week. Q-values, also known as FDR-adjusted (or FDR-corrected) p-values, represent the probability that the given difference would be a false discovery; they represent the expected proportion of “false positives” that would be seen among the collection of all differences whose q-values were at or below the given q-value. Item non-responses are not reported in the tables but are included in the prevalence calculations.

Table S16. MAGA affiliation and firearm ownership

Condition	Non-MAGA, Non-Republican		MAGA Republican		MAGA Supporter, Republican	
	Unweighted n	Weighted % (95% CI)	Unweighted n	Weighted % (95% CI)	Unweighted n	Weighted % (95% CI)
Ownership						
Nonowner without firearms at home	2799	74.1 (72.6,75.7)	404	42.1 (38.4,45.8)	171	39.5 (34.1,45.0)
Owner	1601	17.1 (15.9,18.2)	826	44.1 (40.7,47.6)	416	45.7 (40.5,50.9)
Nonowner with firearms at home	307	8.2 (7.1,9.2)	73	10.7 (8.1,13.4)	36	10.3 (6.3,14.3)
aPD (95% CI; q-value)	Referent		22.4 (18.8,26.0; <0.001)		23.5 (18.3,28.7; <0.001)	
Recency of firearm purchase*						
Purchases 2020 or later	404	31.3 (28.0,34.6)	361	45.0 (40.6,49.3)	149	42.0 (35.6,48.4)
Purchases only 2019 or earlier	1197	68.7 (65.4,72.0)	465	55.0 (50.7,59.4)	267	58.0 (51.6,64.4)
aPD (95% CI; q-value)	Referent		18.4 (13.1,23.8; <0.001)		13.8 (6.9,20.7; <0.001)	

Table S16, continued.

Condition	MAGA Supporter, Non-Republican		Non-MAGA, Republican		Unclassified	
	Unweighted n	Weighted % (95% CI)	Unweighted n	Weighted % (95% CI)	Unweighted n	Weighted % (95% CI)
Ownership						
Nonowner without firearms at home	105	65.4 (57.2,73.6)	690	55.5 (52.4,58.6)	120	64.3 (56.5,72.2)
Owner	101	27.9 (20.3,35.5)	877	31.1 (28.4,33.7)	56	12.9 (8.2,17.5)
Nonowner with firearms at home	9	5.1 (1.3,8.8)	117	11.3 (9.2,13.5)	11	4.3 (1.7,6.9)
aPD (95% CI; q-value)	11.8 (4.0,19.6; 0.007)		10.5 (7.7,13.4; <0.001)		2.6 (-3.6,8.7; 0.48)	
Recency of firearm purchase*						
Purchases 2020 or later	46	48.8 (33.7,63.8)	308	36.5 (32.1,40.8)	11	27.2 (9.1,45.3)
Purchases only 2019 or earlier	55	51.2 (36.2,66.3)	569	63.5 (59.2,67.9)	44	70.6 (52.5,88.8)
aPD (95% CI; q-value)	16.7 (2.3,31.2; 0.09)		6.6 (1.3,12.0; 0.07)		-1.8 (-21.6,18.0; 0.93)	

* Asked of owners only

Adjusted prevalence differences (aPDs) are absolute percentage point (pp) differences for the following responses: item 1, “owner”; item 2, “purchases 2020 or later,” and are adjusted for age, race and ethnicity, gender, education, income, Census division, rurality, military history, number of non-traffic arrests, and total number of drinks per week. Q-values, also known as FDR-adjusted (or FDR-corrected) p-values, represent the probability that the given difference would be a false discovery; they represent the expected proportion of “false positives” that would be seen among the collection of all differences whose q-values were at or below the given q-value. Item non-responses are not reported in the tables but are included in the prevalence calculations.
